# Global Changes in *Staphylococcus aureus* Gene Expression in Human Blood

**DOI:** 10.1371/journal.pone.0018617

**Published:** 2011-04-15

**Authors:** Natalia Malachowa, Adeline R. Whitney, Scott D. Kobayashi, Daniel E. Sturdevant, Adam D. Kennedy, Kevin R. Braughton, Duncan W. Shabb, Binh An Diep, Henry F. Chambers, Michael Otto, Frank R. DeLeo

**Affiliations:** 1 Laboratory of Human Bacterial Pathogenesis, Rocky Mountain Laboratories, National Institute of Allergy and Infectious Diseases, National Institutes of Health, Hamilton, Montana, United States of America; 2 Genomics Unit, Research Technologies Section, Rocky Mountain Laboratories, National Institute of Allergy and Infectious Diseases, National Institutes of Health, Hamilton, Montana, United States of America; 3 Division of Infectious Diseases, Department of Medicine, University of California San Francisco, San Francisco, California, United States of America; 4 Laboratory of Human Bacterial Pathogenesis, National Institute of Allergy and Infectious Diseases, National Institutes of Health, Bethesda, Maryland, United States of America; Indian Institute of Science, India

## Abstract

*Staphylococcus aureus* is a leading cause of bloodstream infections worldwide. In the United States, many of these infections are caused by a strain known as USA300. Although progress has been made, our understanding of the *S. aureus* molecules that promote survival in human blood and ultimately facilitate metastases is incomplete. To that end, we analyzed the USA300 transcriptome during culture in human blood, human serum, and trypticase soy broth (TSB), a standard laboratory culture media. Notably, genes encoding several cytolytic toxins were up-regulated in human blood over time, and *hlgA*, *hlgB*, and *hlgC* (encoding gamma-hemolysin subunits HlgA, HlgB, and HlgC) were among the most highly up-regulated genes at all time points. Compared to culture supernatants from a wild-type USA300 strain (LAC), those derived from an isogenic *hlgABC*-deletion strain (LACΔ*hlgABC*) had significantly reduced capacity to form pores in human neutrophils and ultimately cause neutrophil lysis. Moreover, LACΔ*hlgABC* had modestly reduced ability to cause mortality in a mouse bacteremia model. On the other hand, wild-type and LACΔ*hlgABC* strains caused virtually identical abscesses in a mouse skin infection model, and bacterial survival and neutrophil lysis after phagocytosis *in vitro* was similar between these strains. Comparison of the cytolytic capacity of culture supernatants from wild-type and isogenic deletion strains lacking *hlgABC*, *lukS/F-PV* (encoding PVL), and/or *lukDE* revealed functional redundancy among two-component leukotoxins in vitro. These findings, along with a requirement of specific growth conditions for leukotoxin expression, may explain the apparent limited contribution of any single two-component leukotoxin to USA300 immune evasion and virulence.

## Introduction

*Staphylococcus aureus* is a leading cause of human bacterial infections worldwide, many of which are caused by methicillin-resistant *S. aureus* (MRSA) (reviewed in refs. [1], [2]). Bacteremia is one of the most abundant syndromes caused by MRSA, especially in healthcare settings [3]. For example, Klevens et al. reported that 75.2% of all invasive MRSA infections, including those that originate from community- or healthcare settings, are bacteremias [3]. A strain known as pulsed-field type USA300 is a leading cause of community-associated MRSA (CA-MRSA) infections and an abundant cause of healthcare-associated bloodstream infections in the United State and Canada [3]–[7]. The molecules that contribute to the ability of USA300 (and *S. aureus* in general) to survive in human blood and cause significant human disease remain incompletely defined.

To gain an enhanced understanding of the mechanisms used by USA300 to survive in blood and cause human disease, we analyzed the USA300 transcriptome during culture in human blood and serum in vitro. Although there were a limited number of genes encoding putative or proven virulence molecules up-regulated during culture in blood, transcripts encoding gamma-hemolysin (*hlgA*, *hlgB*, and *hlgC*) were among the most highly up-regulated USA300 genes over a 2-h culture period in human blood. Inasmuch as the relative contribution of gamma-hemolysin to USA300 virulence is not known, we compared the ability of USA300 wild-type and isogenic *hlgABC* deletion strains to circumvent killing by human polymorphonuclear leukocytes and ultimately cause host cell lysis. In addition, we compared the ability of these wild-type and mutant strains to cause abscesses and sepsis in mouse models of infection. Isogenic deletion of genes encoding multiple USA300 leukotoxins (*hlgABC*, *lukSF-PV*, and *lukDE*) singly and in combination indicated functional redundancy exists among these molecules.

## Results

### USA300 survival in human blood

**Figure 1:**
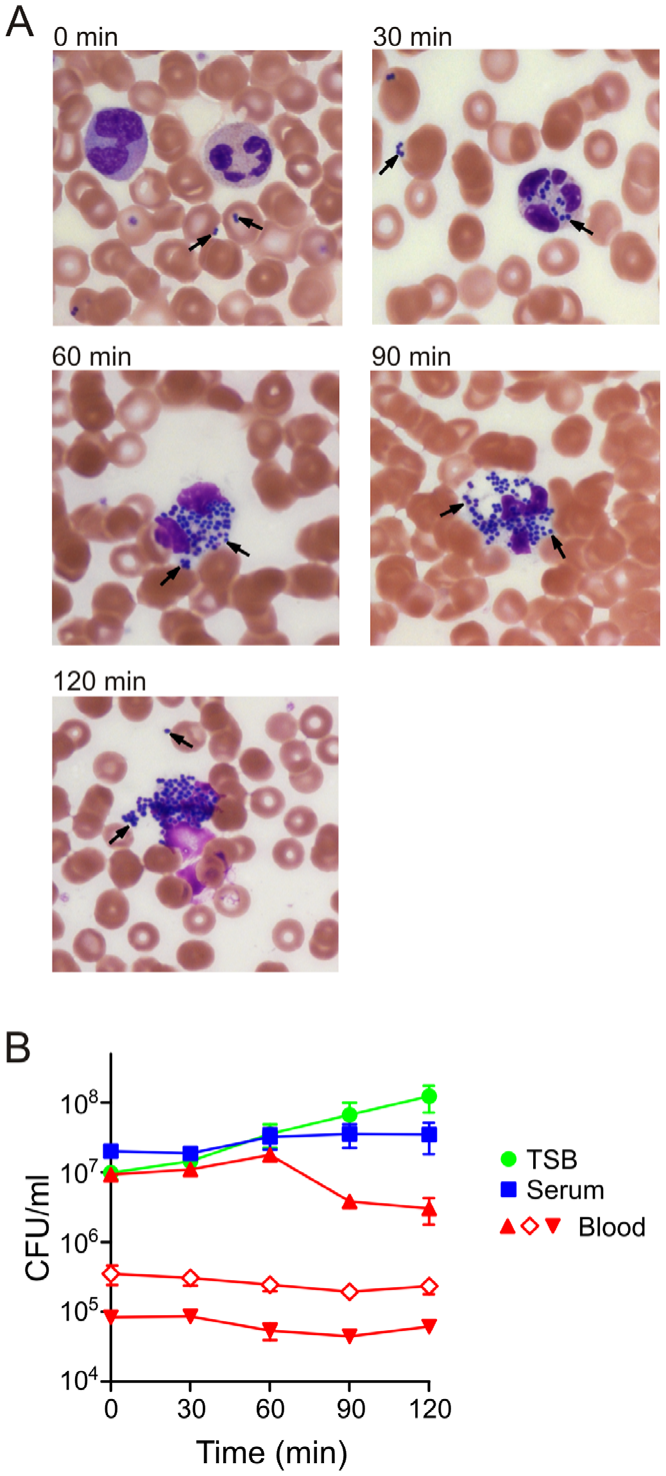
Survival of USA300 in human blood. (A) Interaction of USA300 with PMNs in blood. Blood smears from each time point were prepared and cells were stained with a modified Wright-Giemsa. Black arrows indicate selected *S. aureus*. (B) Growth/survival curves of USA300 cultured in human heparinized blood, human serum and TSB. Prior to inoculation, bacteria were grown in TSB to mid-exponential phase of growth. Cultures were incubated at 37°C with 5% CO_2_ for the indicated time points and plated for determination of CFUs as described in Methods.

As a step toward gaining an enhanced understanding of the ability of USA300 to cause disseminated disease, we investigated survival of the pathogen in human blood *ex vivo* (Fig. 1). Using light microscopy, we determined that the USA300 strain LAC was ingested rapidly by phagocytic leukocytes—largely neutrophils—in human blood (Fig. 1A). At 30 min, some bacteria remained free and not bound by host phagocytic cells, whereas by 60 min there were virtually no free bacteria and most neutrophils were loaded with bacteria (Fig. 1A). Within 90 min, some of the LAC-containing (or associated) neutrophils had lysed and by 120 min there were few remaining intact neutrophils (Fig. 1A). These findings are consistent with the known ability of USA300 to cause rapid neutrophil lysis after phagocytosis [8], [9].

Comparable colony forming units (CFUs) of USA300 were recovered after 60 min of culture in TSB, serum, and heparinized human blood (see cultures seeded at 10^7^ CFUs per ml, Fig. 1B). Although there was a decrease in LAC CFUs by 90 min and 120 min of culture in human blood at the highest inoculum used, this phenomenon was at least in part attributed to aggregation of blood cells and bacteria (discussed below). Taken together, these data indicate there was survival of USA300 in human blood after 120 min in culture (Fig. 1B).

### *S. aureus* transcriptome dynamics in human blood and serum

**Figure 2:**
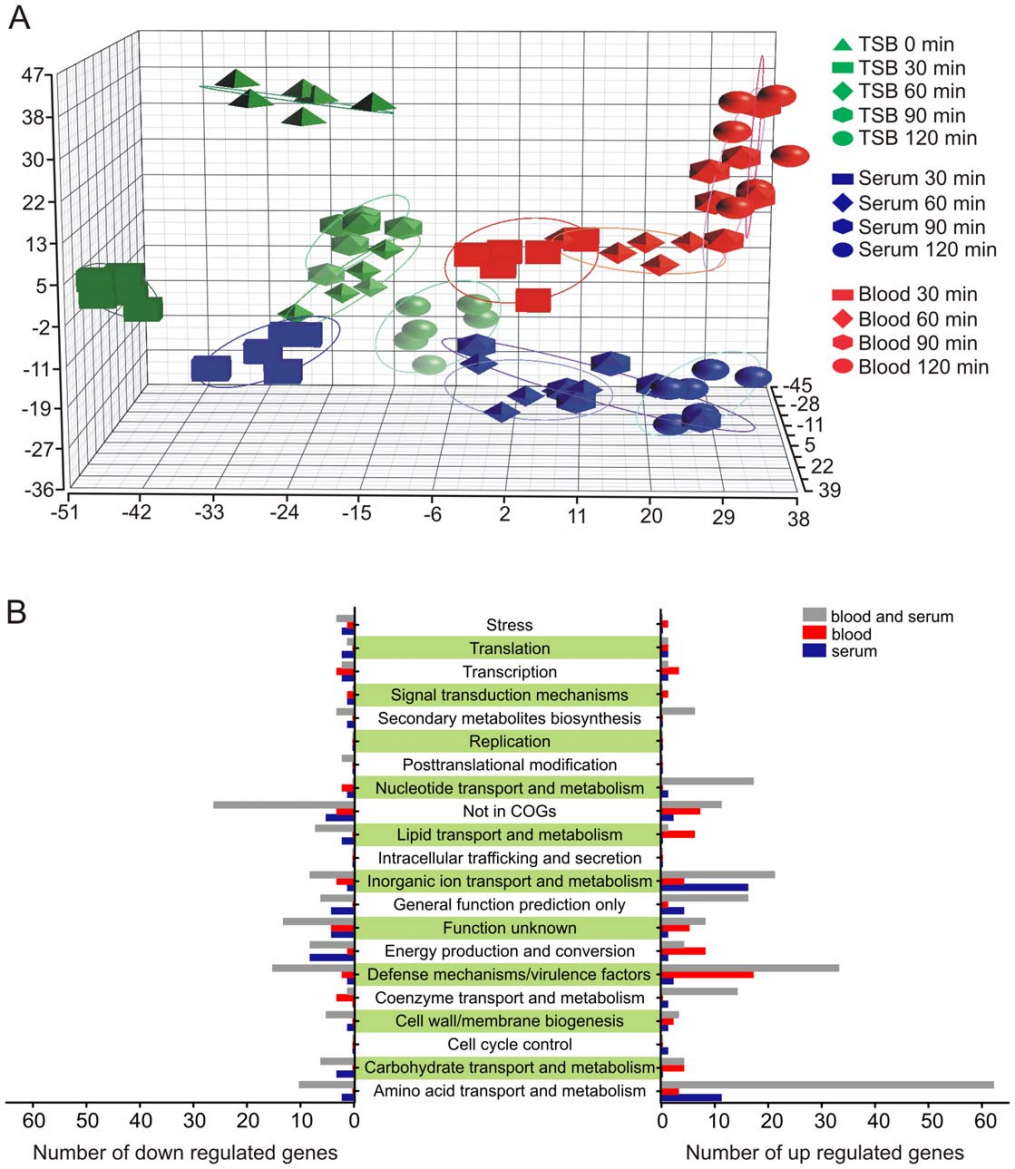
Transcriptome dynamics of USA300 in human blood and serum. (A) Principal component analysis (PCA) of the *S. aureus* transcriptome during culture in human blood, serum or TSB. Each shape represents an individual GeneChip (sample), with the plotted location based upon the correlation of each sample relative to the others. The total amount of variation within the data set is 51.1% and each axis represents a different principal component. (B) Numbers of differentially-regulated genes within each COG group.

To obtain a comprehensive view of the molecules that promote *S. aureus* survival in whole blood and thus facilitate bacteremia, we measured global changes in USA300 gene expression during culture in heparinized human blood and fresh serum using USA300-specific oligonucleotide microarrays (Fig. 2 and Table S1). Principal component analysis (PCA) indicated significant segregation of bacterial transcripts depending on culture conditions, i.e., the transcriptomes of USA300 cultured in TSB, serum, or blood were clearly different (Fig. 2A). Genes were classified into functional groups using clusters of orthologous groups of proteins (COGs) (http://www.ncbi.nlm.nih.gov/COG/) and subjected to further analyses (Fig. 2B). Compared with bacteria at the start of culture (t = 0), genes involved in amino acid transport and metabolism, defense mechanisms/virulence, and inorganic ion transport and metabolism were the most numerous up- or down-regulated molecules during culture in human blood and/or serum (Fig. 2B). Within these groups, genes encoding proteins and enzymes that facilitate biotin metabolism and valine, leucine and isoleucine biosynthesis pathways were the most numerous differentially-regulated molecules during culture in blood or serum.

**Figure 3:**
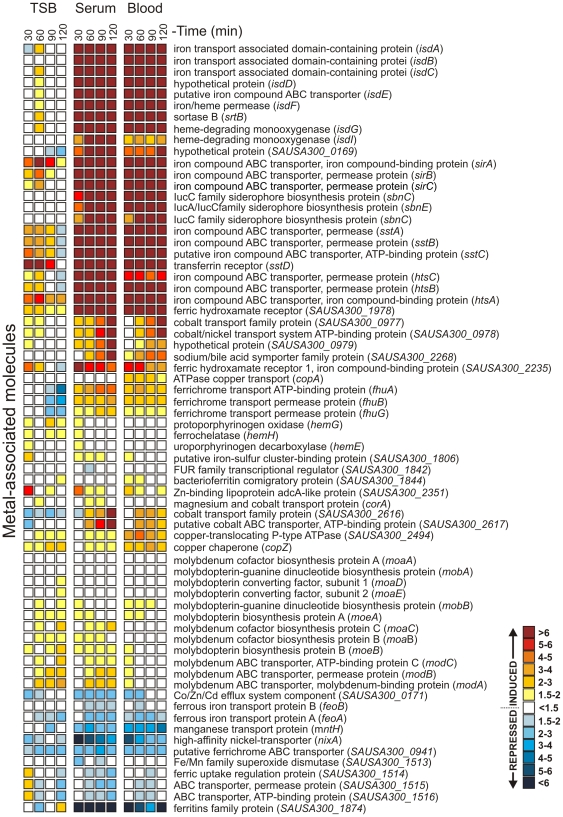
USA300 transcripts encoding metal-associated proteins are differentially expressed following culture in serum or human blood. Microarray results are presented as the mean fold-change of six separate experiments (blood and serum from six individuals) compared to time point zero (t = 0). All transcripts met criteria for differentially-regulated genes as indicated in the Materials and Methods section. Common name of the encoded protein is provided and gene names or ORF numbers are indicated in parentheses.

Transcripts encoding proteins involved in iron or heme uptake and/or binding, such as *isdA*, *isdB*, *isdC*, *isdD*, *isdF*, *srtB*, *isdG isdI*, *fhuA*, *fhuB*, *fhuG*, *sirA*, *sirB*, and *sirC*, were highly up-regulated by USA300 during culture in serum or blood compared with bacteria at the start of culture (t = 0) (Fig. 3 and Table S1). Iron-regulated surface determinants (Isd) of *S. aureus* are known to be critical for binding, uptake and utilization of heme from host proteins [10], [11]. The ferric hydroxamate uptake (Fhu) system [12]–[14] and staphylococcal iron-regulated proteins (Sir) A, B, and C (SirABC) [15], [16] promote ferrisiderophore import in *S. aureus* (reviewed by Beasley and Heinrichs [17]). Blood and serum contain low levels of free iron, thereby explaining the increased levels of transcripts that promote heme and iron uptake by USA300. We note that previous transcriptome studies with *Yersinia* spp. cultured in human plasma reported an increase in transcripts encoding proteins involved in iron uptake [18], [19].

**Figure 4:**
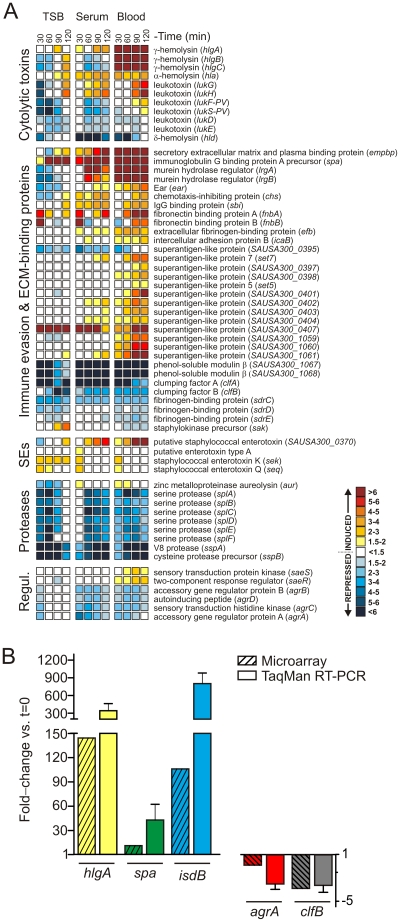
Changes in the expression of USA300 transcripts encoding virulence/defense and gene regulation factors during culture in blood. (A) Microarray data are presented as described in the legend of Fig. 3. (B) Verification of microarray data by TaqMan real-time reverse-transcriptase PCR. Results are expressed as the average relative fold-change in transcripts from bacteria cultured in blood at 90 min compared to TSB culture at t = 0. The relative quantification of *S. aureus* transcript was determined by the change in expression of target transcripts relative to *gyrB* as described in Methods.

A relatively limited number of genes encoding proven or putative *S. aureus* virulence factors were up-regulated uniquely in human blood or serum over the course of the 2-h culture period (versus t = 0) (Fig. 4A). For example, transcripts encoding 12 super-antigen like proteins, IgG-binding protein Sbi (*sbi*), Ear (*ear*), intercellular adhesion protein B (*icaB*), extracellular fibrinogen-binding protein (*efb*), chemotaxis-inhibiting protein (*chp*), murein hydrolase regulator A and B (*lrgA*, *lrgB*), leukotoxin GH (*lukG*, *lukH*) [20], alpha-hemolysin (*hla*), Panton-Valentine leukocidin (*lukS-PV*, *lukF-PV*) and gamma hemolysin subunits A, B, and C (*hlgA*, *hlgB*, *hlgC*) were up-regulated during culture in serum and/or human blood compared with growth in TSB (Fig. 4A). Notably, *hlgA*, *hlgB*, and *hlgC* were among the most highly up-regulated molecules in blood (e.g., *hlgA* was up-regulated 145-fold at 90 min). The increased levels of *hlgA*, *hlgB*, and *hlgC* were either specific to blood or most pronounced in blood, and correspondingly, most of the bacteria were rapidly ingested by PMNs. These findings are consistent with our previous studies that reported up-regulation of these molecules during PMN phagocytosis and in response to neutrophil granule protein extracts [9], [21].

To verify microarray results, five genes of interest were subjected to TaqMan real-time quantitative reverse-transcriptase PCR (Fig. 4B). For this purpose, we compared levels of *hlgA*, *isdB*, *spa*, *agrA*, and *clfB* transcript by microarray and TaqMan analyses after 90 min in culture with human blood (mRNA levels at 90 min were compared to those at the start of the assay, t = 0). There was a strong positive correlation between TaqMan and microarray data.

### Gamma-hemolysin (HlgABC) contributes to USA300-mediated lysis of human neutrophils

**Figure 5:**
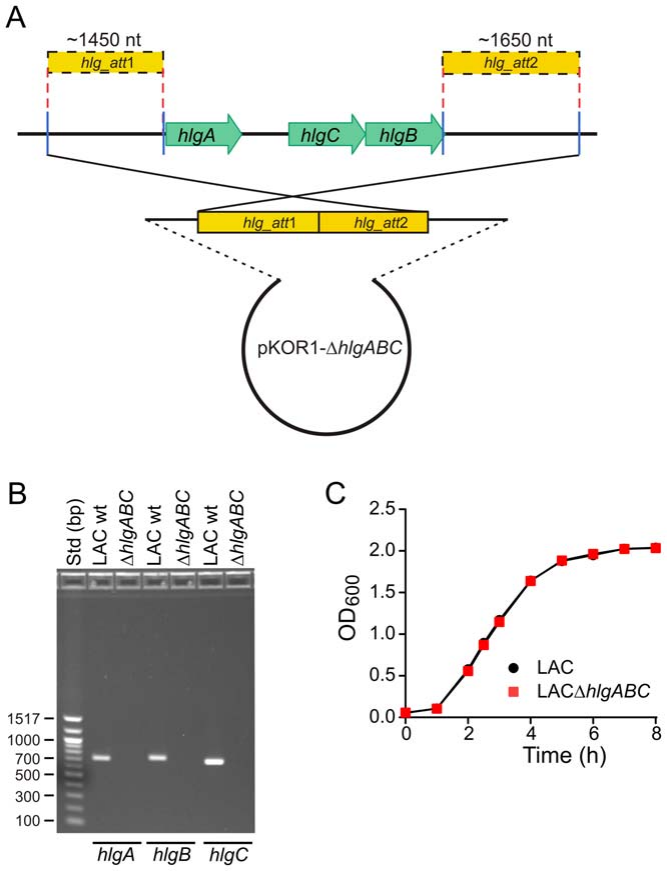
Construction and characterization of an isogenic *hlgABC* deletion strain in USA300. (A) Schematic for deletion *hlgABC* in strain LAC. (B) PCR confirmation of *hlgABC* deletion mutagenesis. (C) Growth curves of the LAC wild-type and isogenic *hlgABC* deletion strain (LACΔ*hlgABC*) cultured in TSB.

We next generated an isogenic *hlgABC* deletion strain of USA300 (LACΔ*hlgABC* or Δ*hlgABC*) to investigate the potential role of this toxin in USA300 virulence and immune evasion (Fig. 5A and B). Growth of the wild-type and Δ*hlgABC* strains was virtually identical in vitro (Fig. 5C). Because of the size of the *hlgABC* operon, we were unable to generate a complemented mutant strain for subsequent studies. However, direct comparison of wild-type and Δ*hlgABC* strains by USA300-specific expression microarrays indicated that the only difference between the two strains was the absence of *hlgABC* transcript in the mutant strain (these data are provided on the Gene Expression Omnibus at http://www.ncbi.nlm.nih.gov/geo, GSE25454).

**Figure 6:**
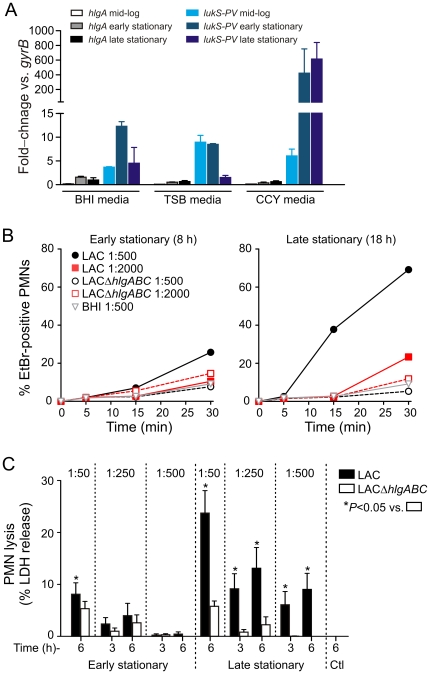
HlgABC causes PMN pore formation and cytolysis. (A) TaqMan real-time reverse transcriptase PCR analysis of *hlgA* and *lukS-PV* expression in BHI, TSB and CCY media at mid-exponential (mid-log), early- and late stationary phases of growth. The relative quantification of *S. aureus* genes was determined by the change in expression of target transcripts relative to that of *gyrB*. Membrane permeability (B) and lysis (C) of human PMNs exposed to USA300 (LAC) BHI culture supernatants. PMNs were incubated with fresh BHI or BHI culture supernatants from wild-type and Δ*hlgABC* strains as indicated. Early stationary or late stationary growth phase culture supernatants were diluted 1∶500 and 1∶2000 for pore formation assays or 1∶50, 1∶250, and 1∶500 for PMN lysis assays. *P≤0.05 vs. Δ*hlgABC*.

Compared with TSB or CCY culture media, brain-heart infusion media (BHI) supported optimum expression of *hlgA* transcript (Fig. 6A). Therefore, we compared BHI culture supernatants from wild-type and Δ*hlgABC* strains for their ability to induce formation of pores in the plasma membrane of human PMNs (Fig. 6B). Although BHI culture supernatants from early stationary phase of growth (7-h cultures) caused limited pore formation (25.8±15.2% at 30 min), those from wild-type strains cultured to late stationary phase of growth (17-h cultures) caused membrane pores in 69.2% of the PMNs (Fig. 6B). By comparison, Δ*hlgABC* supernatants had significantly decreased capacity to cause plasma membrane pores in human PMNs (5.3±2.7% for the mutant strain at 30 min, *P*<0.001 vs. wild-type) (Fig. 6B). Consistent with these findings, LACΔ*hlgABC* culture supernatants had significantly reduced capacity to cause ultimate PMN lysis (e.g., LDH release for a 1∶50 dilution of wild-type supernatants at 6 h was 23.7±10.6% versus 5.8±2.5% from that of the mutant strain) (Fig. 6C). Taken together, these data indicate HlgABC can contribute significantly to USA300-mediated lysis of human PMNs under specific culture conditions.

### Contribution of *hlgABC* to virulence in mouse infection models

**Figure 7:**
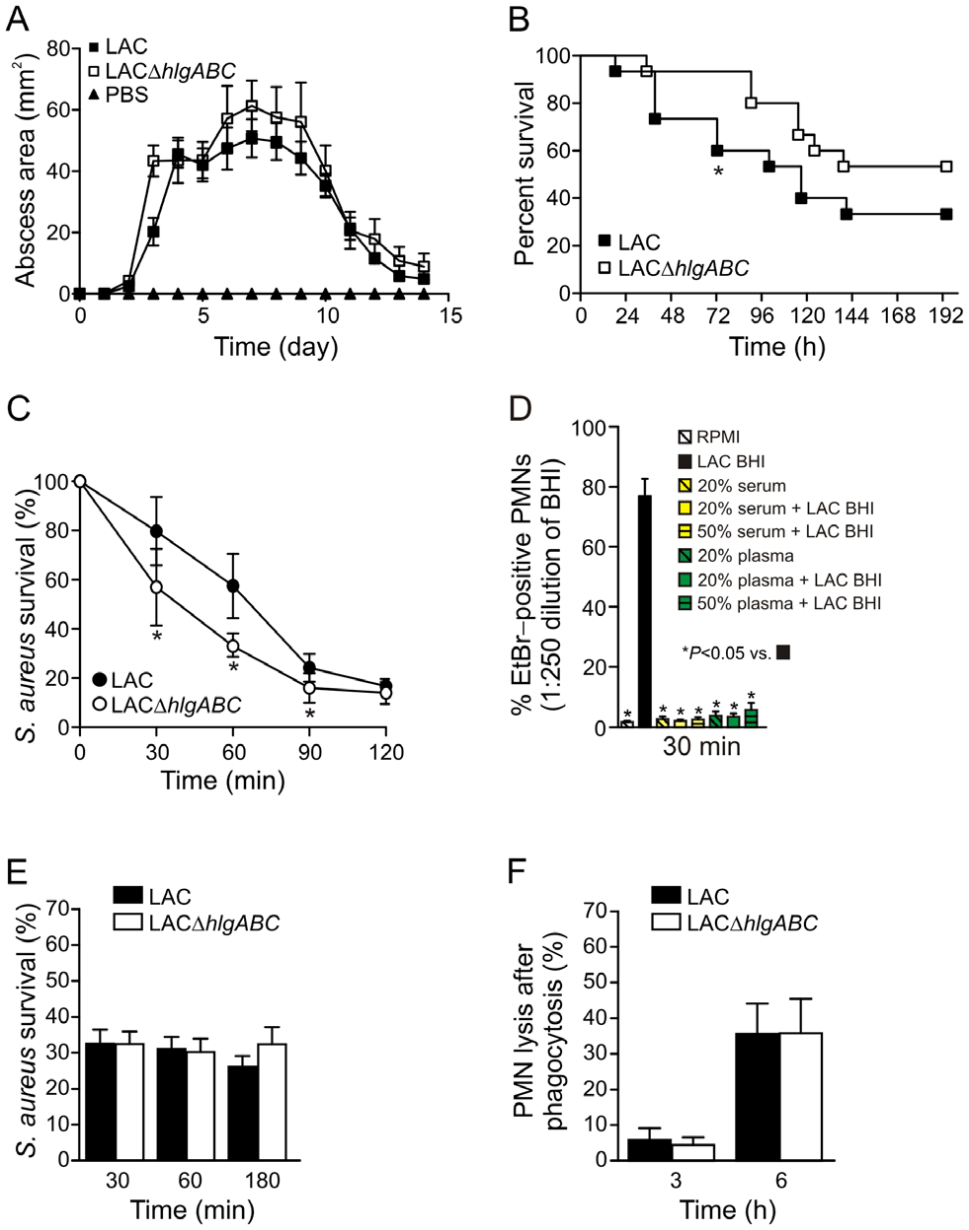
Contribution of *hlgABC* to USA300 virulence in mouse infection models and survival in human blood. (A) Abscess model. Mice (15 per group) were infected with 1×10^7^ CFUs or wild-type or Δ*hlgABC* strains by subcutaneous injection into the right flank. Each symbol represents average abscess area ± SE on the indicated day. (B) Mouse survival. Mice (15 per group) were infected with 5×10^7^ CFUs of wild-type or Δ*hlgABC* strains by tail vein injection and health was monitored for 14 d. *P<0.05 vs. Δ*hlgABC*. (C) Survival of wild-type and Δ*hlgABC* strains in heparinized human blood. There were 1.5×10^7^ LAC wild-type CFUs at 0 min and this number decreased to 2.4×10^6^ CFUs by 120 min. *P<0.05 vs. wild-type strain. Results are the mean ± SE of 4 assays (4 blood donors). (D) Membrane permeability of human PMNs exposed to USA300 (LAC) BHI culture supernatants in human serum or plasma. Supernatants from late stationary phase cultures of LAC were diluted 1∶250 in RPMI (LAC BHI), serum (serum + LAC BHI), or plasma (plasma + LAC BHI) as indicated and pore formation was measured as described in Methods. Control assays contained PMNs in RPMI (RPMI), serum (20%), or plasma (20%) alone. Results are the mean ± SE of 3 experiments. *P≤0.05 vs. LAC BHI. (E) Bacterial survival and PMN lysis (F) after phagocytic interaction with wild-type or Δ*hlgABC* strains. Results for panels D and E are presented as the mean ± SE for at least 6 independent experiments.

Based on the observation that *hlgABC* were highly up-regulated during culture of USA300 in human blood, and since HlgABC contributed to human PMN lysis *in vitro*, we compared virulence of USA300 wild-type and Δ*hlgABC* strains in mouse models of skin infection and bacteremia (Fig. 7A and B). Compared with the LAC wild-type strain, LACΔ*hlgABC* caused abscesses that were of comparable size, and the time required for resolution of abscesses was similar between the strains (Fig. 7A). These observations are in accordance with previous studies that evaluated the contribution of Panton-Valentine leukocidin (PVL)—another two-component leukotoxin—to virulence in the mouse skin infection model [22], [23]. On the other hand, the Δ*hlgABC* strain caused moderately reduced mortality compared with the wild-type strain in the mouse bacteremia model at all times 36 h post-infection (for up to 192 h), albeit the difference was significant only at 72 h (Fig. 7B). Consistent with data from the bacteremia model, survival of the wild-type strain in human blood was greater than that of Δ*hlgABC* through 90 min of culture (e.g., survival was 57.5±13.1% for the wild-type strain versus 33.0±5.2% for the Δ*hlgABC* strain at 60 min) (Fig. 7C). Collectively, these results suggest that if HlgABC plays a role in USA300 virulence, the role is relatively minor and/or the toxin is expressed only in specific host environments or disease conditions. Alternatively, it is possible that function of HlgABC is inhibited under certain conditions *in vivo*. Indeed, the capacity of LAC culture supernatants to cause formation of pores in the plasma membrane of human neutrophils was inhibited significantly by the presence of 20–50% human serum or plasma (Fig. 7D), a finding that merits further investigation.

It is also possible that functional redundancy with other two-component leukotoxins (e.g., PVL and LukGH) masks in part any potential contribution of HlgABC to disease in the mouse infection models. In accordance with this notion, there was comparable survival of the USA300 wild-type and Δ*hlgABC* strains after phagocytic interaction with human neutrophils, and correspondingly, lysis of PMNs after phagocytosis was essentially identical (Fig. 7E and F). The idea that there is functional redundancy among these molecules is tested in part below.

### Relative contribution of USA300 leukotoxins to PMN membrane pore formation in human PMNs

**Figure 8:**
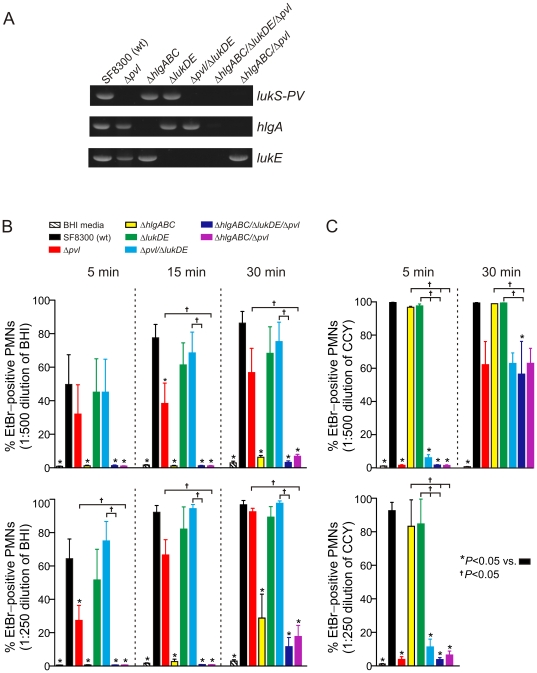
Permeability of PMNs exposed to USA300 (SF8300) culture supernatants. (A) Confirmation of isogenic Δ*pvl*, Δ*hlgABC*, Δ*lukDE*, Δ*pvl*/Δ*lukDE*, Δ*hlgABC*/*lukDE*/Δ*pvl* and Δ*hlgABC*/Δ*pvl* strains by PCR as described in Methods. For these experiments, we used a USA300 wild-type strain known as SF8300 (which by molecular typing methods is essentially identical to LAC) to generate isogenic deletion mutants. (B) PMNs were incubated with late stationary phase BHI culture supernatants at 1∶500 and 1∶250 dilutions. Results are the mean ± SE of at least 5 PMN donors. (C) PMNs were incubated with late stationary phase CCY culture supernatants at 1∶500 and 1∶250 dilutions. Results are the mean ± SE of 2–5 PMN donors. For panels B and C, *P<0.05 versus wild type or †*P*<0.05 as indicated using a repeated-measures ANOVA and Tukey's posttest.

To better understand the level of functional redundancy among USA300 two-component leukotoxins, we compared the ability of USA300 wild-type and Δ*pvl*, Δ*hlgABC*, Δ*lukDE*, Δ*hlgABC/Δpvl*, Δ*pvl/*Δ*lukDE*, or Δ*hlgABC/*Δ*lukDE/*Δ*pvl* isogenic deletion strains for their ability to cause plasma membrane pores in human PMNs (Fig. 8). All USA300 strains were cultured in BHI media to late stationary phase of growth, which promotes optimal expression of *hlgABC* (Fig. 6A). Compared with culture supernatants from the wild-type strain, those from Δ*hlgABC*, Δ*hlgABC/*Δ*pvl*, or Δ*hlgABC/*Δ*lukDE/*Δ*pvl* strains had significantly reduced ability to cause pore formation in human PMNs at all time points tested (Fig. 8B and C). By comparison, BHI culture supernatants from the Δ*pvl* strain had significantly reduced ability to cause PMN pore formation in only 2 of the 6 conditions tested (Fig. 8B). Unexpectedly, culture supernatants from neither the Δ*lukDE* nor Δ*pvl/*Δ*lukDE* strains caused PMN pore formation that was significantly decreased compared to that from the wild-type strain (Fig. 8B). The finding that PMN pore formation caused by culture supernatants from the Δ*lukDE* and Δ*pvl/*Δ*lukDE* strains (and in most assays the Δ*pvl* strain as well) were largely similar to that of the wild-type strain indicates that pore formation in these assays was primarily caused by HlgABC. It should be noted that *in vitro* assay conditions have a significant impact on leukotoxin expression (e.g., see ref. [24]) and similar assays performed with CCY culture media, in which PVL is highly expressed, demonstrated PVL masks any potential role of HlgABC (Fig. 8C). Conversely, longer incubation times using CCY culture supernatants reveal that molecules other than PVL cause PMN pore formation (e.g., see the limited pore formation caused by Δ*pvl* strains at 30 min in Fig. 8C). Taken together, these results provide strong support to the idea that there is functional redundancy among USA300 leukotoxins.

## Discussion

Previous studies used expression microarray approaches to investigate changes in the transcriptomes of group A *Streptococcus* [25], *Streptococcus agalactiae* [26], and *Enterococcus faecalis* during culture in human blood [27]. In addition to generating a comprehensive view of the pathogen response to components of human blood, these previous studies provide an important link between animal models of infection and human disease.

In the present study, we used a similar microarray-based approach to identify changes in the *S. aureus* transcriptome during short-term (up to 2 h) culture in human blood and serum. Our *in vitro* model system is intended to simulate the effects of the host environment imparted on *S. aureus* immediately after entering the bloodstream. We compared *S. aureus* transcriptomes during culture in whole blood or human serum separately as a means to differentiate global responses to host cells present in whole blood versus soluble factors present both in blood and serum. A limitation of our comparison between serum and whole blood is that serum lacks some of the soluble components present in plasma and whole blood (fibrinogen and clotting factors), and *S. aureus* is known to bind fibrinogen. Also, acute-phase proteins produced during human infection will be absent in serum or plasma from healthy individuals and these molecules could ultimately alter *S. aureus* gene expression.

Although USA300 CFUs decreased at later time points (90–120 min) during culture in blood, there was still significant survival of the pathogen (∼3×10^6^ CFUs or 32% after 120 min) (Fig. 1B). There are two possible explanations for these observations. First, these findings might be explained in part by the ability of PMNs to kill some of the ingested USA300, since we demonstrated previously that human PMNs kill about 50–70% of ingested USA300 in vitro [8], [9], [23]. Alternatively, we observed that USA300 caused significant aggregation and clumping of host cells and bacteria in blood at late time points (Fig. 1A) and this phenomenon would decrease the number of recovered CFUs, since it was not possible to completely disaggregate clumps of bacteria.

In addition to producing molecules directed to circumvent the host immune system, *S. aureus* must adapt to the relatively limited nutrient conditions in the bloodstream. A major growth limitation in tissue fluids and blood is very low levels of free iron (10^−18^ M), and bacteria require iron at 0.4 µM to 4.0 µM [11], [28]. Indeed, our USA300 transcriptome analysis revealed that iron and iron transport associated molecules are highly up-regulated during incubation with human blood or serum, even at the earliest time point measured (30 min) (Fig. 3 and Table S1). In addition to adjusting to iron deficiency in blood or serum, *S. aureus* alters expression of many genes encoding proteins involved in general metabolism, findings consistent with transcriptome remodeling by group A *Streptococcus*, *Streptococcus agalactiae*, and *Enterococcus faecalis* during culture in human blood [25]–[27]. To support this “metabolic reset”, up to 30% of all genes within a given COG category, including secondary metabolites biosynthesis, nucleotide transport and metabolism, amino acid transport and metabolism, inorganic ion transport and metabolism, and coenzyme transport and metabolism, were up-regulated by USA300 during culture in blood or serum (Fig. 2B). Interestingly, genes encoding the global regulators accessory gene regulator (Agr) and staphylococcal accessory regulator (Sar) were down regulated during culture in human blood or serum (but not in TSB cultures performed in parallel), whereas *saeR* and *saeS* (*saeRS*) transcripts were up-regulated (Fig. 4A and Table S1). The finding that genes encoding SaeRS are up-regulated in blood only is consistent with previous studies that indicate the genes are up-regulated during phagocytosis by human PMNs.

Expression of staphylococcal virulence and defense factors is largely controlled by two-component gene regulatory systems and other global regulators, and gamma-hemolysin is no exception. Yamazaki et al. [29] reported that expression of gamma-hemolysin is regulated by Sae, observations compatible with our data indicating that *saeRS* and *hlgABC* are up-regulated in blood. Gamma-hemolysin is a unique two-component leukotoxin in the sense that there are three genes encoded by the operon [30]–[32]. Two genes (*hlgA* and *hlgC*) encode LukS subunits and the other gene (*hlgB*) encodes a LukF subunit [30]–[32]. These transcripts, especially *hlgA*, are among the most up-regulated genes during culture of USA300 in whole blood (*hlgA* was up-regulated 145-fold at 90 min, *hlgB* and *hlgC* transcripts increased 34-fold compared to t = 0) (Table S1). These data suggest that gamma-hemolysin contributes to immune evasion and/or facilitates USA300 survival in blood.

Deletion of *hlgABC* did not affect survival of USA300 following phagocytosis by human PMNs nor did it alter the ability of the pathogen to cause PMNs lysis after phagocytosis (Fig. 7E and F). Nonetheless, mice inoculated with LACΔ*hlgABC* had on average 20% increased survival in the bacteremia model compared with mice infected with the LAC wild-type strain, and the mutant strain had reduced survival in human blood (Fig. 7B and C). The modest contribution of gamma-hemolysin to *S. aureus* virulence is consistent with observations made in previous studies of the role of gamma-hemolysin in animal infection models [33], [34]. These observations—i.e., lack of a role for *hlgABC* in USA300 survival after phagocytosis and modest contribution to virulence in the mouse—may seem at variance, but might be explained by differences in toxin production and accumulation. Alternatively, gamma-hemolysin could promote enhanced survival in blood by causing erythrocyte lysis and thereby facilitate iron acquisition by *S. aureus*. Gamma-hemolysin (HlgA + HlgB) was reported previously as the most potent *S. aureus* hemolysin in vitro [35] and iron acquisition is known to be a critical component of *S. aureus* survival in the host [36], [37]. The idea that hemolysins are required for iron acquisition during infection is supported by recent studies of Torres *et al.*, who demonstrated that there is coordinated expression of molecules involved in hemolysis/cytolysis and iron uptake in *S. aureus* [38]. These findings are in accordance with our microarray data (Fig. 3 and 4).

It is also possible that the role of individual USA300 leukotoxins in animal infection models is largely masked by their redundancy with the other two-component leukocidins or other cytolytic molecules such as phenol-soluble modulins (PSMs) [39]. Leukotoxin-specific phenotypes were revealed during culture of USA300 with media and conditions that selectively favor high production of either HlgABC or PVL (Fig. 8B and C). These data suggest that the importance and/or level of expression of a particular leukotoxin *in vivo* is influenced by the extracellular environment and/or surrounding tissue. We recently reported that PMN pore formation or lysis assays performed *in vitro* with *S. aureus* culture supernatants have limited ability to inform about the relative contribution of PVL to pathogenesis *in vivo* [24]. In accordance with this notion, BHI culture supernatants from the Δ*hlgABC* strains had dramatically reduced ability to cause PMN pore formation or lysis (Fig. 6 and Fig. 8), but the contribution of *hlgABC* to virulence was limited by comparison (Fig. 7A–C). Therefore, *in vitro* culture conditions for *S. aureus* and those in vivo are likely much different, making extrapolation of *in vitro* results with animal infections models less predictable. In any case, more work will be required to determine the specific role of this protein during *S. aureus* infection.

The global changes in USA300 gene expression reported here provide a comprehensive view of the molecules that potentially promote *S. aureus* immune evasion and survival in blood. We focused our efforts on one finding from the microarray data (changes in *hlgABC* expression), but investigation of the role of other differentially-regulated molecules is likely to provide new insights into the pathogenesis of *S. aureus* infection and may identify new therapeutic targets.

## Materials and Methods

### Human subjects and ethics statement

Heparinized venous blood or venous blood without anticoagulant was obtained from healthy human volunteers. The Institutional Review Board for Human Subjects, National Institute of Allergy and Infectious Diseases, National Institutes of Health, approved these studies (protocol 01-I-N055). Each person included in the study provided written consent prior to enrollment.

### Bacterial strains and culture

#### Culture conditions

Unless specified otherwise, bacteria were grown in trypticase soy broth (TSB; Becton Dickinson) in a flask-to-media volume ratio of 5∶1 at 225 rpm at 37°C. Microarray analyses were performed using a *S. aureus* USA300 strain known as the Los Angeles County clone (LAC) [9]. To determine the optimal culture conditions for *hlgABC* expression, LAC was cultured in TSB, brain heart infusion media (BHI; Becton Dickinson) and CCY media (3% yeast extract, 2% Bacto-casamino acids, 0.21 M sodium pyruvate, 44 mM dibasic sodium phosphate, 3 mM monobasic potassium phosphate, pH 6.7) to the desired phase of growth. A USA300 Δ*hlgABC* isogenic deletion strain was constructed as described below. For PMN assays, bacteria were cultured in BHI media to early stationary (7 h) or late stationary (17 h) phase of growth.

#### Construction of an isogenic hlgABC deletion strain (ΔhlgABC)

Isogenic *hlgABC* deletion in USA300 strain LAC strain (LACΔ*hlgABC* or Δ*hlgABC*) was generated by a published allelic replacement method [40]. Regions flanking the *hlgABC* locus were amplified by PCR using the following primers:

Upstream fragment (1450 bp)

hlg_att1-F, 5′-**GGGGACAAGTTTGTACAAAAAAGCAGGCT**GATAGAAGCCAACAAGTTTGGGTAG-3′
hlg_att1-R, 5′-CATAGAAATCACTTTCTTTCTATTTAATCTGCAGTTC-3′

Downstream fragment (1650 bp)

hlg_att2-F, 5′-GATACAAAAGAAACTGCAGACAATAAATAGCTAG-3′
hlg_att2-R, 5′-**GGGGACCACTTTGTACAAGAAAGCTGGGT**GCATGTGGTGCATACGGGGGTGT-3′

The *att* sequence is in bold-face type and restriction sites are underlined. PCR products were digested with *Pst*I and ligated and cloned into pKOR1. The resulting plasmid, pKOR1Δ*hlgABC*, was amplified in *E. coli* and electroporated into USA300 strain LAC and allelic replacement was performed as described [40]. Isogenic deletion of *hlgABC* in LACΔ*hlgABC* strains was verified by PCR. A USA300-specific Affymetrix oligonucleotide microarray was used to compare LAC and LACΔ*hlgABC* strains during growth in TSB (posted online at GSE25454). Using this approach, *hlgA*, *hlgB*, and *hlgC* were the only USA300 transcripts with a significant change in expression, thereby providing strong evidence that no unintended mutations were generated in the LACΔ*hlgABC* strain.

#### Construction of double- and triple mutant USA300 strains

A USA300 strain known as SF8300 [41], which is essentially identical to strain LAC by molecular typing methods, was used to construct single, double, and triple-deletion strains. The isogenic *lukS-PV* and *lukF-PV* deletion strain (SF8300Δ*pvl* or Δ*pvl*) was published previously [42] and was created as described by Voyich et al. [23]. Isogenic deletion of *hlgA*, *hlgB*, and *hlgC* (Δ*hlgABC*), *lukD* and *lukE* (Δ*lukDE*), Δ*hlgABC/*Δ*pvl*, Δ*pvl/*Δ*lukDE*, or Δ*hlgABC/*Δ*lukDE/*Δ*pvl* was performed in strain SF8300 or SF8300Δ*pvl* using a previously reported allelic replacement method [40], essentially as described above for the LACΔ*hlgABC* strain.

### Microarray experiments

*S. aureus* strain LAC was cultured to mid-exponential phase of growth (OD_600_∼0.75), washed once with Dulbecco's PBS (DPBS; Sigma-Aldrich) and added to fresh TSB media, 100% normal human serum, or human heparinized venous blood to a final concentration 10^7^ colony-forming units (CFU)/ml. Blood or serum from 6 individuals was used for these experiments. Bacteria were incubated with gentle rotation for up to 120 min at 37°C with 5% CO_2_. At selected time points (0, 30, 60, 90 and 120 min) 10-ml samples were collected for subsequent analyses. A 1-ml aliquot of each sample was centrifuged at 1500× g (4000 rpm) for 5 min to pellet all bacteria, washed once with 1 ml of DPBS, centrifuged again and resuspended in 1 ml of DPBS. 100 µl of the sample was plated on trypticase soy agar (TSA) to enumerate CFUs. A small fraction of the culture samples was used to prepare a sample smear that was subsequently stained with Wright-Giemsa and examined under a microscope (AxioSkop 2 Plus, Carl Zeiss). The remaining sample was used for isolation of bacterial RNA.

*S. aureus* RNA was isolated from samples using an RNeasy Mini Kit (Qiagen) combined with Bacterial-RNA Protect (Qiagen) as described by the manufacturer. Contaminating chromosomal DNA was removed by TurboDNase (Ambion/Applied Biosystems) and samples were treated to MICROB*Enrich*™ (Ambion) to remove eukaryotic RNA. Fragmented and biotin-dUTP-labeled amplified mRNA was generated from purified staphylococcal RNA using the MessageAmp™ II-Bacteria Kit (Ambion) according to the manufacturer's protocol. Samples were hybridized to a custom Affymetrix GeneChip (RMLchip7, Gene Expression Omnibus (GEO) platform GPL8069, http://www.ncbi.nlm.nih.gov/projects/geo/) that contains all open reading frames of the USA300 genome (100% coverage, 2560/2560 total ORFs). Samples were scanned using the Affymetrix 7Gplus GeneChip scanner according to standard GeneChip protocol with the image files converted using GeneChip Operating Software (GCOS v1.4). The TSB samples at time zero (t = 0)—i.e., bacteria cultured to mid-exponential phase of growth as described above and washed once but not incubated at 37°C—were used as controls for all other samples and time points. Thus, all expression microarray data are presented as the mean fold-change of 6 experiments relative to that at t = 0. The data were quantile-normalized and a 3-way ANOVA with multiple test correction using the false discovery rate (FDR, significance at 0.05) [43] was performed using Partek Genomics Suite software (Partek, inc. St. Louis, Mo., v6.5 6091110). These data were combined with fold change values, signal confidence (above background), and call consistency (as a percent) as calculated using custom Excel templates to generate final gene lists for each comparison (Table S1). A complete set of microarray data has been posted online at http://www.ncbi.nlm.nih.gov/projects/geo/ under series number GSE25454. All microarray data are MIAME compliant.

### TaqMan Real-Time Reverse Transcriptase PCR

RNA samples used in the microarray experiments were also analyzed by TaqMan real-time RT-PCR. Purified *S. aureus* RNA was subjected to One-Step TaqMan real-time PCR using an ABI 7500 thermocycler (Applied Biosystems). The relative quantification of *S. aureus* mRNA was determined by change in the expression level of the target transcripts relative to *gyrB* transcript (housekeeping gene) [9], in accordance with the manufacturer's protocol (Applied Biosystems Relative Quantification Manual). Data obtained are expressed as the mean fold-change in transcript during culture in blood for 90 min relative to the t = 0 control sample. Results are from three separate blood donors and samples were analyzed in triplicate.

Alternatively, RNA used for comparison of *hlgA* and *lukS-PV* transcripts was isolated from LAC cultured to mid-exponential-, early stationary-, or late stationary phase of growth in TSB, BHI, or CCY media as described above. TaqMan analysis was performed as described above.

### Human neutrophil assays

PMNs were isolated by standard dextran sedimentation coupled with Hypaque-Ficoll gradient centrifugation as described elsewhere [44]. PMN bactericidal activity toward serum-opsonized *S. aureus* and lysis of PMNs following phagocytosis of serum opsonized bacteria were determined using published methods [8], [9], [23]. Lysis of PMNs following exposure to filter-sterilized BHI culture supernatants was determined by release of lactate dehydrogenase (LDH) using a kit (Cytotoxicity Detection Kit, Roche Applied Sciences) as recommended by the manufacturer and as described [23], [24]. PMN plasma membrane permeability (pore formation) caused by USA300 culture supernatants was assessed by uptake of ethidium bromide (EtBr) as described [8], [23], [24].

### Mouse infection models

Female CD1 Swiss and Crl:SKH1-E hairless mice (each 6–8 weeks old) were used for bacteremia and abscess models, respectively. All studies conformed to guidelines set forth by the National Institutes of Health and were reviewed and approved by the Animal Use Committee at Rocky Mountain Laboratories, National Institute of Allergy and Infectious Diseases (protocol 2010–24). Fifteen mice were used for each strain group and five mice were used for the DPBS controls in each infection model. *S. aureus* strains were cultured to mid-exponential phase of growth (OD_600_∼0.75) and washed twice with sterile DPBS. For the skin abscess model, animals were inoculated subcutaneously with 0.05 ml of 1×10^7^ live *S. aureus* or sterile saline in the right flank [22], [23], [45]. Mice were monitored and weighed daily over the period of 14 days. Abscess area was calculated using formula [A = π(L/2)×W/2], where (L) is length and (W) is width of an abscess [46].

For the bacteremia model, animals received 5×10^7^ CFUs of bacteria in 0.1 ml sterile saline by intravenous inoculation via tail-vein injection [23]. Criteria for determining morbidity or sickness in mice include hunched posture, decreased activity, ruffled fur, and labored breathing. Following inoculation, animal health was monitored every 3 h for the first 72 h and then every 8 h thereafter for up to 194 h. Mice were euthanized if they were unable to eat or drink or if they became immobile. In our experience, mice do not recover from intravenous *S. aureus* infection if they meet criteria for euthanasia. Therefore, mice euthanized due to severe bacterial illness were scored as dead.

### Statistical analyses

Statistical analyses were performed using GraphPad Prism 5 (GraphPad Software, Inc., CA). Statistics for PMN pore formation assays were determined using a one-way repeated-measures analysis of variance (ANOVA) and Tukey's posttest. PMN lysis assays and assays for *S. aureus* survival in blood were analyzed using a one-tailed paired *t*-test. Mouse survival statistics were evaluated by two methods. First, survival curves were compared performed using a standard log-rank test. In addition, differences in mouse survival were assessed on each day using Fisher's exact test. Statistics for abscess area were performed using a one-way ANOVA and Bonferroni's posttest for multiple comparisons.

## Supporting Information

Table S1 **Changes in USA300 transcriptome, following culturing in TSB, human serum or human heparinized blood.** Microarray results are presented as the mean fold-change from six separate experiments. Only transcripts with significant fold-change (*p* value<0.05 and ≥2-fold change in transcriptome) are included. All conditions and time points were compared to the control – TSB time point “0” or t = 0.
(DOCX)
