# Supplementary material for: Global Changes in Staphylococcus aureus Gene Expression in Human Blood
Source: PLoS One. 2011 Apr 15;6(4):e18617. doi: 10.1371/journal.pone.0018617 (PMC3078114; doi:10.1371/journal.pone.0018617)
Supplement: Table S1 — Changes in USA300 transcriptome, following culturing in TSB, human serum or human heparinized blood. Microarray results are presented as the mean fold-change from six separate experiments. Only transcripts with significant fold-change (p value<0.05 and ≥2-fold change in transcriptome) are included. All conditions and time points were compared to the control – TSB time point “0” or t = 0. (DOCX) [file pone.0018617.s001.docx]

**Table S1.** Changes in the USA300 transcriptome during culture in human blood, serum and trypticase soy broth (TSB).

| **ORF** | **Gene** | **Description/function** | **TSB (min)** | | | | **Serum (min)** | | | | **Blood (min)** | | | |
| --- | --- | --- | --- | --- | --- | --- | --- | --- | --- | --- | --- | --- | --- | --- |
|  |  |  | (30) | (60) | (90) | (120) | (30) | (60) | (90) | (120) | (30) | (60) | (90) | (120) |
| SAUSA300_0007 | *-* | Sugar kinase | 2.1 |  |  |  |  |  |  |  |  |  |  |  |
| SAUSA300_0008 | *hutH* | Histidine ammonia-lyase (EC 4.3.1.3) |  |  | 2.3 | 2.3 |  | 2.5 |  | 2.0 |  | 2.4 | 3.5 | 2.7 |
| SAUSA300_0009 | *serS* | Seryl-tRNA synthetase (EC 6.1.1.11) | 2.7 | 4.6 | 3.6 | 2.3 | 2.4 | 2.5 | 2.2 | 3.2 |  |  | 3.1 | 2.4 |
| SAUSA300_0010 | *-* | Branched-chain amino acid transport protein AzlC |  |  | -3.3 |  |  | 2.4 | 3.0 | 6.1 |  |  | 2.1 |  |
| SAUSA300_0011 | *-* | Branched-chain amino acid transport protein AzlD |  |  | -2.9 |  |  | 2.2 | 3.0 | 5.9 |  |  | 2.2 |  |
| SAUSA300_0012 | *-* | Homoserine O-acetyltransferase (EC 2.3.1.31) |  |  | -3.2 |  |  |  | 2.6 | 4.8 |  |  |  |  |
| SAUSA300_0017 | *purA* | Adenylosuccinate synthetase (EC 6.3.4.4) | 8.0 | 2.3 | 3.6 |  | 18.0 | 14.2 | 4.6 |  | 13.1 | 8.5 | 3.6 | 3.0 |
| SAUSA300_0023 | *-* | Hypothetical protein |  |  |  |  |  |  |  |  |  |  |  | -2.1 |
| SAUSA300_0024 | *-* | Hypothetical protein |  |  |  | -2.0 |  |  |  |  | -2.3 | -2.1 |  |  |
| SAUSA300_0025 | *-* | 5'-nucleotidase (EC 3.1.3.5) |  |  | 3.4 | 4.8 | 2.9 | 5.5 | 4.4 | 3.6 | 3.0 | 2.3 |  |  |
| SAUSA300_0026 | *-* | Hypothetical protein |  |  | 2.4 |  |  |  |  |  |  |  |  |  |
| SAUSA300_0027 | *-* | Hypothetical protein |  |  | 2.0 |  |  |  |  |  |  |  |  |  |
| SAUSA300_0030 | *-* | Glycerophosphoryl diester phosphodiesterase (EC 3.1.4.46) |  |  |  | 2.1 |  |  |  |  |  |  |  |  |
| SAUSA300_0036 | *-* | Hypothetical protein |  |  |  |  |  |  |  |  |  |  |  | 2.3 |
| SAUSA300_0037 | *ccrB* | Site-specific recombinase |  |  | -2.0 |  |  |  |  | -2.0 |  |  |  |  |
| SAUSA300_0040 | *-* | Hypothetical protein |  |  | -2.0 |  |  |  |  |  |  |  |  |  |
| SAUSA300_0044 | *-* | Hydroxyacylglutathione hydrolase (EC 3.1.2.6) |  |  |  |  |  |  |  |  |  |  |  | 2.4 |
| SAUSA300_0045 | *-* | Hypothetical protein |  |  | -2.0 |  |  |  |  |  |  |  |  |  |
| SAUSA300_0053 | *speG* | Spermidine N1-acetyltransferase (EC 2.3.1.57) | 4.4 | 4.0 | 3.1 | 3.8 | 3.9 | 3.9 | 4.4 | 3.5 | 2.7 | 2.3 |  |  |
| SAUSA300_0054 | *-* | Hypothetical protein |  |  |  |  |  |  |  |  |  |  | 2.0 | 2.5 |
| SAUSA300_0055 | *-* | Alcohol dehydrogenase (EC 1.1.1.1) |  |  |  |  |  | 8.5 | 11.9 | 25.3 | 15.0 | 31.8 | 34.1 | 22.5 |
| SAUSA300_0066 | *argR* | Arginine repressor, ArgR |  |  |  | 2.3 |  |  |  |  |  |  |  |  |
| SAUSA300_0067 | *-* | Putative transcription factors | -5.8 | -2.4 |  | -3.7 | -13.1 | -7.5 | -7.1 | -6.4 | -2.4 | -2.8 |  |  |
| SAUSA300_0068 | *-* | Lead, cadmium, zinc and mercury transporting ATPase (EC 3.6.3.-) |  | -2.0 |  |  |  | -2.1 |  |  | -3.4 | -2.8 | -2.4 | -3.2 |
| SAUSA300_0070 | *-* | Lysophospholipase L2 (EC 3.1.1.5) |  |  |  |  | -3.2 | -3.1 | -2.6 | -2.8 | -2.4 | -2.7 |  |  |
| SAUSA300_0071 | *-* | Transposase |  |  |  |  |  |  |  |  |  |  |  | 2.0 |
| SAUSA300_0072 | *-* | Hypothetical protein | 2.7 |  |  |  | 3.0 | 2.2 |  |  |  |  |  |  |
| SAUSA300_0073 | *-* | Nickel-binding protein | 2.6 |  |  |  | 2.9 | 2.1 |  |  |  |  |  |  |
| SAUSA300_0074 | *opp-3B* | Hypothetical protein | 2.5 |  |  |  | 2.7 |  |  |  |  |  |  |  |
| SAUSA300_0075 | *opp-3C* | Dipeptide transport system permease protein DppC | 2.3 |  |  |  | 2.6 |  |  |  |  |  |  |  |
| SAUSA300_0076 | *-* | Hypothetical protein | 2.2 |  |  |  | 2.3 |  |  |  |  |  |  |  |
| SAUSA300_0077 | *-* | Nickel transport ATP-binding protein NikE | 2.1 |  |  |  | 2.2 |  |  |  |  |  |  |  |
| SAUSA300_0078 | *copA* | Copper-silver efflux ATPase (EC 3.6.3.-) |  |  |  |  |  |  |  |  | 2.2 | 2.7 | 2.1 |  |
| SAUSA300_0079 | *-* | Hypothetical exported protein |  |  |  |  |  |  |  |  |  | 2.5 | 2.2 |  |
| SAUSA300_0083 | *-* | Hypothetical membrane spanning protein |  |  |  |  |  |  |  | 2.0 |  |  |  |  |
| SAUSA300_0084 | *-* | Hypothetical cytosolic protein |  |  |  |  |  |  |  | 2.3 |  |  |  |  |
| SAUSA300_0085 | *-* | Rhodanese-related sulfurtransferases / Hypothetical protein |  |  | -2.2 |  |  |  |  |  |  |  |  |  |
| SAUSA300_0086 | *-* | Hydroxyacylglutathione hydrolase (EC 3.1.2.6) |  |  | -2.1 |  |  |  |  |  |  |  |  |  |
| SAUSA300_0088 | *-* | Hypothetical protein | 3.1 |  |  |  | 2.1 |  |  |  |  |  |  |  |
| SAUSA300_0089 | *-* | Nitrogen regulation protein NIFR3 | 2.5 |  |  |  | 2.0 |  |  |  |  |  |  |  |
| SAUSA300_0093 | *-* | Transcriptional regulators, LysR family |  | 2.2 |  | 2.5 |  | 2.1 |  |  |  | 2.9 |  |  |
| SAUSA300_0094 | *-* | Hypothetical protein |  |  |  |  |  |  |  |  |  | -2.0 | -2.0 |  |
| SAUSA300_0097 | *-* | Superfamily I DNA helicase |  | 3.4 | 3.7 | 3.3 | 2.8 | 6.9 | 6.5 | 7.8 | 3.9 | 3.8 | 3.0 | 2.8 |
| SAUSA300_0098 | *-* | Hypothetical protein |  |  |  |  |  | 2.2 | 2.3 | 2.6 |  |  |  |  |
| SAUSA300_0099 | *plc* | 1-phosphatidylinositol phosphodiesterase precursor (EC 4.6.1.13) | -3.6 | -3.4 |  | 3.4 |  | -2.3 |  |  | -2.2 | -5.0 | -4.4 | -3.4 |
| SAUSA300_0105 | *-* | Carboxypeptidase, M20(D) family |  |  | 2.1 |  |  |  | 2.1 | 2.2 |  |  |  | 2.7 |
| SAUSA300_0107 | *-* | Sodium-dependent phosphate transporter | 6.2 |  |  |  |  |  | 2.8 | 2.9 |  |  |  |  |
| SAUSA300_0108 | *sok* | Myosin-crossreactive antigen |  |  | 3.9 | 2.4 |  |  |  | 2.6 |  |  | 2.4 | 4.9 |
| SAUSA300_0109 | *-* | Transporter, drug/metabolite exporter family |  |  |  |  |  |  |  |  | 2.2 |  |  | 3.1 |
| SAUSA300_0110 | *-* | Transcriptional regulator, GntR family / tyrosine aminotransferase (ec 2.6.1.5) |  |  |  | 2.2 |  |  | 2.1 | 3.0 |  |  |  |  |
| SAUSA300_0111 | *-* | Hypothetical protein | -2.0 |  |  |  | -2.2 | -2.2 |  |  | -2.5 | -2.6 |  |  |
| SAUSA300_0113 | *spa* | Immunoglobulin G binding protein A precursor |  | 10.6 | 11.6 | 13.4 | 5.5 | 14.3 | 16.1 | 16.8 | 8.9 | 13.4 | 11.9 | 9.6 |
| SAUSA300_0114 | *-* | Staphylococcal accessory regulator A | 2.5 | 3.1 | 2.8 | 2.5 |  |  | 2.2 | 2.0 |  |  |  |  |
| SAUSA300_0115 | *sirC* | Staphylobactin transport system permease protein |  | 3.2 |  |  | 10.7 | 14.6 | 15.3 | 15.3 | 9.0 | 12.5 | 9.8 | 9.1 |
| SAUSA300_0116 | *sirB* | Staphylobactin transport system permease protein | 2.0 | 4.0 |  |  | 22.9 | 33.8 | 32.7 | 32.7 | 20.0 | 24.8 | 17.8 | 17.4 |
| SAUSA300_0117 | *sirA* | Staphylobactin-binding protein | 4.2 | 8.0 | 5.1 |  | 54.7 | 78.3 | 80.6 | 59.7 | 55.9 | 67.5 | 45.4 | 47.1 |
| SAUSA300_0118 | *-* | Cysteine synthase (EC 2.5.1.47) |  |  |  |  | 11.6 | 79.1 | 72.1 | 85.0 | 14.5 | 24.2 | 24.5 | 42.4 |
| SAUSA300_0119 | *-* | Ornithine cyclodeaminase family protein |  |  |  |  | 5.7 | 50.0 | 43.9 | 53.2 | 8.7 | 14.3 | 15.1 | 27.1 |
| SAUSA300_0120 | *sbnC* | Siderophore biosynthesis IucC protein (EC 6.-.-.-) |  |  |  |  | 5.3 | 60.9 | 54.1 | 61.9 | 8.3 | 18.8 | 23.3 | 36.7 |
| SAUSA300_0121 | *-* | Multidrug resistance efflux pump |  |  |  |  | 3.0 | 28.6 | 24.8 | 25.3 | 4.1 | 6.9 | 8.9 | 13.4 |
| SAUSA300_0122 | *sbnE* | IucA/IucC family siderophore biosynthesis protein |  |  |  |  | 4.2 | 58.3 | 47.0 | 43.1 | 6.5 | 17.1 | 23.9 | 39.4 |
| SAUSA300_0123 | *sbnF* | IucC family siderophore biosynthesis protein |  |  |  |  | 3.3 | 46.4 | 35.1 | 31.3 | 4.0 | 11.5 | 16.2 | 26.4 |
| SAUSA300_0124 | *-* | 4-hydroxy-2-oxovalerate aldolase (EC 4.1.2.-) |  |  |  |  | 3.2 | 52.6 | 40.9 | 35.7 | 3.9 | 11.6 | 17.0 | 25.5 |
| SAUSA300_0125 | *-* | Diaminopimelate decarboxylase (EC 4.1.1.20) |  |  |  |  | 2.5 | 48.4 | 35.3 | 33.5 | 3.3 | 9.7 | 14.2 | 22.6 |
| SAUSA300_0126 | *-* | Siderphore biosynthesis protein SbnI |  |  |  |  | 2.2 | 35.3 | 28.9 | 27.6 | 2.9 | 10.1 | 14.8 | 23.3 |
| SAUSA300_0127 | *-* | Hypothetical protein |  |  |  |  |  |  |  | -2.1 |  |  | -2.3 |  |
| SAUSA300_0128 | *-* | Hypothetical protein | 2.5 |  | 2.5 |  |  |  |  |  |  |  |  |  |
| SAUSA300_0129 | *-* | (R,R)-butanediol dehydrogenase (EC 1.1.1.4) / Acetoin dehydrogenase (EC 1.1.1.5) | -3.1 |  |  |  | -2.2 | 2.5 | 4.4 | 7.3 | 2.5 | 5.7 | 10.0 | 10.3 |
| SAUSA300_0135 | *-* | Superoxide dismutase (EC 1.15.1.1) | -2.4 |  |  |  |  |  |  |  |  |  |  |  |
| SAUSA300_0136 | *-* | Hypothetical protein | -14.0 | -6.6 | -2.4 | -3.7 | -9.2 | -8.1 | -8.1 | -11.3 | -3.6 | -6.4 | -6.6 | -5.1 |
| SAUSA300_0137 | *-* | Transcriptional regulator, GntR family |  |  | 2.7 | 3.0 |  | 2.0 |  |  |  |  |  |  |
| SAUSA300_0138 | *deoD* | Purine nucleoside phosphorylase (EC 2.4.2.1) |  | 3.8 | 6.9 |  | -2.6 | -3.6 | -2.7 | -3.3 |  |  | 4.5 | 7.2 |
| SAUSA300_0139 | *-* | Permease |  |  | 3.6 |  |  | -2.5 |  | -2.1 |  |  | 3.8 | 5.5 |
| SAUSA300_0142 | *phnE* | Phosphonates transport system permease protein PhnE |  |  |  |  |  |  |  |  |  |  |  | 2.0 |
| SAUSA300_0148 | *-* | Hypothetical protein |  |  |  |  |  |  |  |  |  |  |  | 2.3 |
| SAUSA300_0151 | *adhE* | Alcohol dehydrogenase (EC 1.1.1.1) / Acetaldehyde dehydrogenase [acetylating] (EC 1.2.1.10) |  | 2.6 | 30.2 | 61.2 |  | 4.9 | 11.4 | 14.3 |  |  | 5.4 | 13.9 |
| SAUSA300_0152 | *cap5A* | Hypothetical protein |  |  |  |  |  |  | 2.7 | 7.8 |  |  |  | 2.2 |
| SAUSA300_0153 | *cap5B* | Tyrosine-protein kinase (capsular polysaccharide biosynthesis) |  |  |  |  |  |  | 2.3 | 6.1 |  |  |  |  |
| SAUSA300_0154 | *cap5C* | Phosphotyrosine-protein phosphatase (capsular polysaccharide biosynthesis) (EC 3.1.3.48) |  |  |  |  |  |  |  | 2.8 |  |  |  |  |
| SAUSA300_0157 | *cap5F* | UDP-2-acetamido-2,6-dideoxy-beta-L-talose 4-dehydrogenase (EC 1.1.1.-) |  |  |  |  |  |  |  |  |  | -2.0 |  |  |
| SAUSA300_0160 | *cap5I* | Glycosyltransferase (EC 2.4.1.-) | -2.2 | -2.0 |  |  | -2.2 | -2.4 | -2.3 |  |  | -2.3 | -2.2 |  |
| SAUSA300_0161 | *cap5J* | Capsular polysaccharide synthesis protein Cap5J |  |  |  |  |  | -2.2 |  |  | -2.4 | -2.5 | -2.2 |  |
| SAUSA300_0162 | *cap5K* | Capsular polysaccharide synthesis protein Cap5K |  |  |  |  |  |  |  |  | -2.2 | -2.3 | -2.2 |  |
| SAUSA300_0163 | *cap5L* | Hypothetical protein |  |  |  |  |  |  |  |  | -2.3 | -2.4 |  |  |
| SAUSA300_0168 | *isdI* | Heme-degrading monooxygenase IsdI |  |  |  |  | 3.7 | 6.9 | 8.7 | 10.2 | 2.9 | 3.1 | 2.8 | 3.5 |
| SAUSA300_0169 | *-* | Hypothetical membrane spanning protein |  |  |  | -2.4 | 4.3 | 8.2 | 10.2 | 10.9 | 4.5 | 4.9 | 4.2 | 6.0 |
| SAUSA300_0170 | *-* | Aldehyde dehydrogenase B (EC 1.2.1.22) | -20.2 | -8.0 | -10.8 | -19.2 | -16.4 | -21.1 | -24.0 | -28.2 |  | 2.1 |  |  |
| SAUSA300_0171 | *-* | Cobalt-zinc-cadmium resistance protein CzcD | -2.3 |  |  |  | -2.8 | -2.7 | -2.3 | -2.0 | -2.5 | -2.3 |  |  |
| SAUSA300_0172 | *-* | Hypothetical protein | -4.0 | -2.4 |  | -2.2 | -13.3 | -19.4 | -20.1 | -22.7 | -5.0 | -10.2 | -9.5 | -8.6 |
| SAUSA300_0173 | *-* | alpha-helical coiled-coil protein SrpF | -6.9 |  | 2.0 | 5.6 | -27.8 | -30.8 | -14.1 | -5.4 | -8.9 | -5.0 | -7.4 | -7.9 |
| SAUSA300_0174 | *-* | ABC transporter ATP-binding protein | -13.9 |  |  | 4.1 | -32.9 | -47.4 | -24.5 | -10.3 | -13.1 | -7.5 | -13.1 | -14.6 |
| SAUSA300_0175 | *-* | ABC transporter substrate-binding protein | -14.8 |  |  | 3.0 | -30.0 | -34.1 | -19.6 | -10.6 | -12.7 | -8.3 | -12.3 | -15.3 |
| SAUSA300_0176 | *-* | ABC transporter permease protein | -12.6 |  |  | 2.3 | -18.1 | -28.1 | -15.3 | -9.2 | -9.3 | -7.7 | -11.6 | -10.7 |
| SAUSA300_0177 | *-* | Isovaleryl-CoA dehydrogenase (EC 1.3.99.10) | -15.8 |  |  | 2.3 | -30.0 | -33.6 | -21.1 | -10.8 | -19.2 | -13.1 | -15.3 | -16.2 |
| SAUSA300_0178 | *-* | Hypothetical cytosolic protein | -4.3 | -2.2 | -2.1 |  | -2.8 | -6.4 | -7.1 | -5.6 | -2.0 |  | -2.5 | -2.8 |
| SAUSA300_0179 | *-* | NAD-dependent formate dehydrogenase (EC 1.2.1.2) | -4.8 | -5.0 | -5.5 | -3.6 | -5.8 | -7.1 | -8.6 | -7.5 | -3.8 | -4.7 | -4.1 | -3.7 |
| SAUSA300_0180 | *-* | Multidrug resistance protein ImrP | -3.0 | -2.2 | -2.5 |  | -2.8 |  |  |  | -2.8 | -2.2 |  |  |
| SAUSA300_0181 | *-* | Surfactin synthetase subunit 2 | -2.6 | -3.8 | -3.4 | -2.1 |  | -2.6 | -2.8 | -2.7 | -2.4 | -3.1 | -2.6 | -2.6 |
| SAUSA300_0182 | *-* | 4'-phosphopantetheinyl transferase (EC 2.7.8.-) | -2.9 | -4.3 | -4.3 | -2.5 | -2.8 | -3.7 | -3.5 | -3.3 | -2.9 | -3.5 | -2.8 | -3.1 |
| SAUSA300_0183 | *-* | Hypothetical membrane associated protein | -3.6 |  | 3.2 | 3.7 | -3.7 |  |  |  | -3.1 | -5.0 |  |  |
| SAUSA300_0187 | *rocD* | Ornithine aminotransferase (EC 2.6.1.13) |  |  |  |  |  |  |  | 2.1 |  |  |  |  |
| SAUSA300_0188 | *brnQ* | Branched-chain amino acid transport system carrier protein |  |  |  |  | 2.3 | 4.1 | 4.0 | 4.5 | 3.1 | 3.6 | 2.5 | 2.3 |
| SAUSA300_0189 | *entB* | Isochorismatase family protein | -3.5 | -2.7 | -3.0 | -3.8 | -6.2 | -7.3 | -7.0 | -5.7 | -2.4 |  |  | -2.1 |
| SAUSA300_0190 | *ipdC* | Indole-3-pyruvate decarboxylase (EC 4.1.1.74) | -2.6 | -2.8 | -3.4 | -4.2 | -3.2 | -3.6 | -3.4 | -2.8 |  |  |  |  |
| SAUSA300_0191 | *ptsG* | PTS system, glucose-specific IIABC component (EC 2.7.1.69) | 2.7 |  | 2.6 |  | 3.8 |  |  |  | 2.3 |  |  |  |
| SAUSA300_0192 | *-* | outer surface protein | -3.5 |  | 3.0 |  | -2.5 | -4.2 | -2.5 | -3.9 |  |  |  | 2.4 |
| SAUSA300_0193 | *murQ* | Glucokinase regulatory protein | -4.5 | -2.2 |  |  | -2.6 | -4.3 | -3.0 | -3.6 |  |  |  | 2.0 |
| SAUSA300_0194 | *-* | PTS system, sucrose-specific IIBC component (EC 2.7.1.69) | -2.5 |  |  |  | -2.1 | -3.4 | -2.6 | -2.7 |  |  |  |  |
| SAUSA300_0195 | *-* | Transcriptional regulator, RpiR family |  |  | 2.0 |  |  | -3.2 | -2.7 | -2.7 |  |  |  |  |
| SAUSA300_0196 | *hsdR* | Type I restriction-modification system restriction subunit (EC 3.1.21.3) |  | 2.6 | 2.6 | 2.7 | 2.5 | 3.5 | 3.4 | 3.6 | 3.1 | 2.6 |  |  |
| SAUSA300_0198 | *-* | Hypothetical cytosolic protein |  |  | 2.1 |  |  | 2.1 | 2.2 | 2.1 |  |  |  |  |
| SAUSA300_0199 | *-* | Hypothetical protein |  | 2.4 | 2.8 | 2.6 |  | 3.1 | 3.4 | 3.4 | 2.3 | 2.3 | 2.6 | 2.1 |
| SAUSA300_0200 | *-* | Oligopeptide transport ATP-binding protein OppD / Oligopeptide transport ATP-binding protein OppF |  |  |  | 3.7 | -2.0 | -2.0 |  | 2.0 |  |  |  |  |
| SAUSA300_0201 | *-* | Oligopeptide transport system permease protein OppB |  |  |  |  |  |  | 2.8 | 8.0 |  | 2.7 |  | 2.1 |
| SAUSA300_0202 | *-* | Oligopeptide transport system permease protein OppC | -2.0 |  |  |  | -2.2 |  |  | 6.3 |  |  |  |  |
| SAUSA300_0203 | *-* | Oligopeptide-binding protein OppA | -2.2 |  |  |  | -2.6 |  |  | 6.4 |  |  |  |  |
| SAUSA300_0204 | *ggt* | Gamma-glutamyltranspeptidase (EC 2.3.2.2) |  |  |  |  | -2.0 |  |  | 3.3 |  |  |  |  |
| SAUSA300_0205 | *-* | membrane lipoprotein |  |  |  |  |  |  |  | -2.7 |  |  |  |  |
| SAUSA300_0206 | *-* | FMN-dependent NADH-azoreductase (EC 1.6.5.2) |  |  |  |  |  |  |  |  | 3.0 | 5.0 | 2.8 | 2.1 |
| SAUSA300_0207 | *-* | Lipoprotein nlpd/lppb homolog |  |  | -2.8 |  | -2.7 | -2.9 | -2.4 |  |  | 3.2 | 3.4 |  |
| SAUSA300_0208 | *-* | Hypothetical protein |  | 3.6 | 5.6 |  |  |  | 2.1 |  |  |  | 2.2 |  |
| SAUSA300_0209 | *-* | Maltose/maltodextrin-binding protein | -2.4 |  | 3.0 |  |  |  |  |  |  | 2.0 | 2.4 | 2.7 |
| SAUSA300_0210 | *-* | Maltodextrin transport system permease protein MalC | -4.2 | -2.3 |  | -2.3 |  | -2.9 | -2.2 |  | -2.1 |  |  |  |
| SAUSA300_0211 | *-* | Hypothetical protein | -4.3 | -2.9 |  | -2.7 |  | -3.2 | -2.5 |  | -2.3 |  |  |  |
| SAUSA300_0212 | *-* | NAD-dependent oxidoreductase | -3.9 | -2.9 |  | -3.0 |  | -3.5 | -2.6 | -2.1 | -2.7 | -2.1 |  |  |
| SAUSA300_0213 | *-* | Hypothetical protein | -2.9 |  |  | -2.3 |  | -2.3 |  |  | -2.1 |  |  |  |
| SAUSA300_0214 | *-* | IolE protein homolog |  |  |  |  |  |  |  |  | -2.1 |  |  | 2.1 |
| SAUSA300_0215 | *-* | Hypothetical membrane spanning protein | 10.3 | 2.4 | 2.9 |  | 11.5 | 4.6 | 4.2 |  | 4.1 | 3.8 | 4.2 | 4.9 |
| SAUSA300_0216 | *uhpT* | Hexose phosphate transport protein | -2.2 |  |  |  |  | -2.0 |  |  | 2.3 | 10.6 | 45.5 | 45.4 |
| SAUSA300_0217 | *-* | Two-component response regulator VesN |  |  |  | 2.2 |  |  | 2.0 | 2.2 |  |  |  |  |
| SAUSA300_0218 | *-* | Two-component sensor kinase VesM (EC 2.7.3.-) |  | 2.4 | 2.9 | 3.5 | 2.2 | 2.8 | 3.5 | 3.9 | 2.8 | 2.4 |  | 2.7 |
| SAUSA300_0219 | *-* | Iron(III)-binding protein | 2.4 | 3.8 | 4.5 | 5.7 | 3.0 | 4.2 | 5.3 | 5.6 | 2.7 | 2.5 | 2.1 | 2.0 |
| SAUSA300_0220 | *pflB* | Formate acetyltransferase (EC 2.3.1.54) | -2.0 | 8.6 | 20.5 | 13.5 |  | 6.9 | 15.2 | 16.0 | -3.3 | -3.0 | 9.4 | 19.2 |
| SAUSA300_0221 | *pflA* | Pyruvate formate-lyase activating enzyme (EC 1.97.1.4) | -2.4 | 3.9 | 10.6 | 8.8 |  | 3.6 | 7.5 | 10.0 | -3.8 | -3.9 | 5.5 | 11.0 |
| SAUSA300_0222 | *-* | Hypothetical protein / Glycerophosphodiester phosphodiesterase (EC 3.1.4.46) |  | 2.1 |  | 2.7 |  |  |  | 3.6 |  |  |  | 2.2 |
| SAUSA300_0223 | *-* | Hypothetical protein | 3.7 |  |  | 2.6 | 6.8 | 6.7 | 8.1 | 6.5 | 2.2 | 3.7 | 6.8 | 7.4 |
| SAUSA300_0224 | *coa* | Staphylocoagulase precursor | 11.4 | 3.4 |  |  | 8.7 |  |  |  |  | 2.3 | 3.8 | 3.0 |
| SAUSA300_0225 | *-* | 3-ketoacyl-CoA thiolase (EC 2.3.1.16) |  |  |  |  |  |  |  |  | 2.1 |  | 3.4 | 4.7 |
| SAUSA300_0226 | *-* | Enoyl-CoA hydratase (EC 4.2.1.17) / Delta(3)-cis-delta(2)-trans-enoyl-CoA isomerase (EC 5.3.3.8) / 3-hydroxyacyl-CoA dehydrogenase (EC 1.1.1.35) |  |  |  |  |  |  |  |  |  | 2.5 | 5.5 | 5.8 |
| SAUSA300_0227 | *fadD* | Glutaryl-CoA dehydrogenase (EC 1.3.99.7) |  |  |  |  |  |  |  |  | 2.1 | 2.8 | 7.4 | 7.1 |
| SAUSA300_0228 | *fadE* | Long-chain-fatty-acid--CoA ligase (EC 6.2.1.3) |  |  |  |  |  |  |  |  |  |  | 3.9 | 3.3 |
| SAUSA300_0229 | *-* | Acetyl-CoA:acetoacetyl-CoA transferase alpha subunit (EC 2.8.3.-) |  | -2.1 |  | -2.1 |  |  |  |  | 2.2 | 3.4 | 4.8 | 4.3 |
| SAUSA300_0230 | *-* | Hypothetical membrane spanning protein | 2.7 | 5.4 | 3.2 | 6.3 | 5.8 | 6.3 | 7.5 | 8.8 | 3.6 | 4.1 | 3.0 | 2.7 |
| SAUSA300_0231 | *-* | Dipeptide-binding protein |  |  |  | 2.2 | -2.2 |  | -2.0 | -2.3 | -2.3 | -3.1 | -2.5 | -2.3 |
| SAUSA300_0232 | *-* | Hypothetical cytosolic protein |  |  | 2.2 | 3.1 |  |  |  |  |  |  |  |  |
| SAUSA300_0233 | *-* | Hypothetical protein |  | 3.2 | 6.6 | 9.0 |  | 2.0 | 2.0 |  |  |  |  |  |
| SAUSA300_0234 | *-* | Flavohemoprotein / Dihydropteridine reductase (EC 1.5.1.34) / Nitric oxide dioxygenase (EC 1.14.12.17) |  | 5.2 | 8.9 | 13.2 |  | 3.2 | 3.1 |  |  |  |  |  |
| SAUSA300_0235 | *-* | L-lactate dehydrogenase (EC 1.1.1.27) | -9.4 |  |  |  | -11.4 |  |  |  | -27.4 | -22.6 |  |  |
| SAUSA300_0236 | *-* | PTS system, glucose-specific IIBC component (EC 2.7.1.69) |  |  |  | 2.4 |  | 2.0 | 2.8 | 3.8 |  |  | 2.1 | 2.1 |
| SAUSA300_0237 | *-* | Inosine-uridine preferring nucleoside hydrolase (EC 3.2.2.1) | -2.2 |  |  |  |  |  |  |  |  |  |  |  |
| SAUSA300_0245 | *-* | D-ribitol-5-phosphate cytidylyltransferase (EC 2.7.7.40) | -2.6 |  |  |  |  | 2.6 | 2.6 | 2.2 | 2.2 | 2.3 |  |  |
| SAUSA300_0246 | *-* | Ribitol-5-phosphate 2-dehydrogenase (EC 1.1.1.137) | -2.5 |  |  |  |  | 2.8 | 2.7 | 2.4 | 2.2 | 2.3 |  |  |
| SAUSA300_0247 | *-* | CDP-ribitol ribitolphosphotransferase (EC 2.7.8.14) | -2.0 |  |  |  |  | 3.0 | 2.9 | 2.7 | 3.0 | 3.2 | 2.7 | 2.3 |
| SAUSA300_0253 | *scdA* | Nitric oxide-dependent regulator DnrN |  |  | 4.3 | 7.0 |  | 2.4 | 4.1 | 4.5 |  | -2.0 |  |  |
| SAUSA300_0254 | *-* | Autolysin sensor kinase (EC 2.7.3.-) |  | 2.2 | 2.4 | 4.9 | 2.1 | 2.4 | 3.0 | 4.2 |  |  | 2.0 | 2.2 |
| SAUSA300_0255 | *-* | Autolysin response regulator | 2.6 | 3.2 | 3.7 | 6.3 | 2.8 | 2.8 | 3.6 | 4.5 |  |  |  |  |
| SAUSA300_0256 | *lrgA* | Murein hydrolase exporter | -3.3 | -2.2 |  |  |  | 11.7 | 7.0 | 4.3 | 13.1 | 17.4 | 14.1 | 12.7 |
| SAUSA300_0257 | *lrgB* | Murein hydrolase export regulator | -4.1 | -4.7 |  |  |  | 5.8 | 4.4 | 2.9 | 7.0 | 11.1 | 11.0 | 10.8 |
| SAUSA300_0259 | *-* | PTS system, beta-glucoside-specific IIABC component (EC 2.7.1.69) | 2.0 |  |  |  |  |  |  | 2.4 |  |  |  |  |
| SAUSA300_0260 | *bglA* | 6-phospho-beta-glucosidase (EC 3.2.1.86) | 2.5 |  |  |  |  |  | 2.0 | 3.1 |  | 2.3 | 2.2 | 2.0 |
| SAUSA300_0261 | *-* | Hypothetical protein |  | 2.4 | 3.0 | 2.9 | 5.9 | 18.5 | 36.4 | 54.1 | 4.4 | 5.2 | 8.5 | 16.6 |
| SAUSA300_0262 | *rbsK* | Ribokinase (EC 2.7.1.15) | -2.0 |  |  |  |  |  |  |  |  |  |  |  |
| SAUSA300_0263 | *rbsD* | D-ribose mutarotase (EC 5.1.3.-) |  |  |  |  |  |  |  |  |  |  |  | 2.3 |
| SAUSA300_0264 | *-* | Ribose uptake protein | -2.1 |  |  |  |  |  |  |  |  |  |  | 2.0 |
| SAUSA300_0265 | *-* | Transcriptional repressor | -2.2 |  |  |  | -2.4 |  |  |  | -2.2 | -2.0 |  |  |
| SAUSA300_0266 | *-* | Hypothetical cytosolic protein | 2.4 |  | 2.1 |  | 2.0 | 2.3 |  |  |  |  |  |  |
| SAUSA300_0268 | *-* | Multidrug resistance protein B |  |  |  | -2.4 | 4.5 | 3.0 | 3.2 | 2.8 | 2.8 | 2.8 | 3.0 | 4.2 |
| SAUSA300_0269 | *-* | Choloylglycine hydrolase (EC 3.5.1.24) |  |  |  |  | 2.6 | 4.4 | 4.4 | 4.5 | 2.1 | 3.0 | 2.9 | 2.9 |
| SAUSA300_0270 | *lytM* | Peptidoglycan hydrolase |  |  |  |  |  | 2.4 | 2.8 | 2.8 |  | 3.2 | 3.8 | 3.1 |
| SAUSA300_0271 | *-* | ABC transporter ATP-binding protein |  |  |  |  | 2.5 |  |  |  | 2.4 |  |  |  |
| SAUSA300_0272 | *-* | Hypothetical protein |  |  |  |  | 3.1 |  |  |  | 2.9 |  |  |  |
| SAUSA300_0273 | *-* | Hypothetical protein |  |  |  |  | 3.3 |  |  |  | 2.7 |  |  |  |
| SAUSA300_0274 | *-* | Hypothetical protein | 2.4 | 2.9 | 2.1 |  | 4.7 | 2.9 |  |  | 3.8 | 2.6 |  |  |
| SAUSA300_0277 | *-* | Secretory antigen precursor SsaA | 2.5 | 2.9 | 3.1 | 4.2 | 5.8 | 3.8 | 2.9 | 3.3 | 3.4 | 2.4 |  |  |
| SAUSA300_0279 | *-* | Hypothetical membrane spanning protein |  |  |  |  | 2.1 |  |  |  |  |  | -2.9 | -3.8 |
| SAUSA300_0281 | *-* | Hypothetical protein |  |  |  | 2.8 | 2.1 | 2.5 |  |  | 3.6 | 2.0 |  |  |
| SAUSA300_0282 | *-* | Hypothetical membrane associated protein |  |  |  | 2.5 | 2.1 | 2.1 |  |  | 3.6 | 2.1 |  |  |
| SAUSA300_0283 | *-* | DNA segregation ATPase and related proteins (FtsK/SpoIIIE family) |  |  |  |  |  |  |  |  |  |  |  | -2.5 |
| SAUSA300_0284 | *-* | Hypothetical protein |  |  |  |  |  |  |  |  |  |  |  | -2.1 |
| SAUSA300_0285 | *-* | Hypothetical protein |  |  |  |  |  |  |  |  |  |  |  | -2.2 |
| SAUSA300_0288 | *-* | Hypothetical cytosolic protein |  |  |  |  |  |  |  |  |  |  | -2.2 | -2.5 |
| SAUSA300_0292 | *-* | Hypothetical protein |  |  |  |  |  | -2.1 | -2.6 | -4.1 |  | -2.6 | -4.5 | -6.2 |
| SAUSA300_0293 | *-* | Hypothetical cytosolic protein |  |  |  |  |  |  | -2.0 | -2.0 |  |  | -2.1 |  |
| SAUSA300_0294 | *-* | Hypothetical cytosolic protein |  |  |  |  |  | -2.1 | -2.5 | -2.4 |  | -2.3 | -2.6 | -2.9 |
| SAUSA300_0302 | *-* | Hypothetical cytosolic protein |  |  |  |  |  |  | -2.1 |  |  |  | -2.4 | -2.5 |
| SAUSA300_0303 | *-* | Hypothetical protein |  |  | -2.1 |  |  | -3.0 | -3.2 | -2.6 |  | -2.5 | -3.0 |  |
| SAUSA300_0304 | *-* | Hypothetical protein |  |  |  |  |  | -3.2 | -3.2 | -4.2 | -2.0 | -3.2 | -4.8 | -5.2 |
| SAUSA300_0305 | *-* | Formate/nitrite transporter family protein | -5.0 | 3.9 | 5.4 | 6.2 | -4.5 |  |  |  | -6.3 | -8.1 |  |  |
| SAUSA300_0306 | *brnQ* | Branched-chain amino acid transport system carrier protein | -3.1 | -2.7 | -3.6 | -2.2 |  |  |  |  |  |  |  |  |
| SAUSA300_0307 | *-* | Acid phosphatase (EC 3.1.3.2) | 3.8 | 6.0 | 11.5 | 21.2 | 8.7 | 16.9 | 18.7 | 17.6 | 10.6 | 9.1 | 5.6 | 6.8 |
| SAUSA300_0308 | *-* | ABC transporter permease protein | -3.0 | -2.0 |  | -2.4 | -6.1 | -5.0 | -5.5 | -5.8 | -5.8 | -7.1 | -4.8 | -4.4 |
| SAUSA300_0309 | *-* | ABC transporter ATP-binding protein | -2.6 |  |  |  | -5.3 | -4.2 | -4.2 | -4.3 | -3.4 | -3.7 | -3.0 | -3.3 |
| SAUSA300_0310 | *pfoR* | Transcriptional regulator PfoR |  | 2.4 |  | 2.7 |  |  |  | 2.8 |  |  |  |  |
| SAUSA300_0311 | *-* | Ribokinase (EC 2.7.1.15) |  |  |  |  |  |  |  |  |  |  |  | 2.0 |
| SAUSA300_0312 | *-* | Sugar kinases |  |  |  |  |  |  |  |  | 2.2 |  | 2.0 | 3.2 |
| SAUSA300_0313 | *-* | Nucleoside permease NupC |  |  |  |  |  |  |  |  |  |  |  | 2.5 |
| SAUSA300_0314 | *-* | Hypothetical protein |  |  | 2.2 |  |  |  |  |  |  |  |  | 2.5 |
| SAUSA300_0315 | *nanA* | N-acetylneuraminate lyase (EC 4.1.3.3) |  | 2.1 | 4.0 |  |  |  |  |  |  |  |  | 2.7 |
| SAUSA300_0317 | *-* | Transcriptional regulator, RpiR family |  |  |  | 2.2 |  |  |  |  |  |  |  |  |
| SAUSA300_0318 | *-* | N-acetylmannosamine-6-phosphate 2-epimerase (EC 5.1.3.9) | 2.0 |  |  |  |  |  |  |  |  |  |  |  |
| SAUSA300_0319 | *-* | Hypothetical membrane spanning protein |  |  |  |  |  |  |  |  |  |  | 2.1 | 2.2 |
| SAUSA300_0320 | *-* | Lipase (EC 3.1.1.3) | -5.3 | -3.6 | -2.8 |  | -2.5 |  |  |  |  |  |  |  |
| SAUSA300_0321 | *-* | Acetyl esterase (EC 3.1.1.-) |  |  |  | 2.1 |  |  |  |  |  |  |  |  |
| SAUSA300_0322 | *-* | Probable NADH-dependent flavin oxidoreductase VqiG (EC 1.-.-.-) |  |  |  | 2.6 | 2.0 | 2.1 |  | 2.1 |  |  |  |  |
| SAUSA300_0323 | *-* | Hypothetical protein |  | -2.3 | -2.5 | -2.5 |  |  |  |  |  |  |  |  |
| SAUSA300_0324 | *-* | Luciferase-like monooxygenase (EC 1.14.-.-) |  |  |  | -2.9 |  | 2.3 |  |  |  |  |  |  |
| SAUSA300_0325 | *-* | Glycine cleavage system H protein |  |  |  | -2.4 |  | 2.3 |  |  |  |  |  |  |
| SAUSA300_0326 | *-* | ATPase associated with chromosome architecture/replication |  |  |  | -2.3 |  | 2.2 |  |  |  |  |  |  |
| SAUSA300_0327 | *-* | SIR2 family protein |  |  |  | -2.1 |  | 2.5 |  |  |  |  |  |  |
| SAUSA300_0328 | *-* | Lipoate-protein ligase A (EC 6.3.2.-) |  |  |  | -2.3 |  | 2.3 |  |  |  |  |  |  |
| SAUSA300_0329 | *-* | Cprd14 protein | -2.2 |  |  |  |  |  |  |  |  |  |  |  |
| SAUSA300_0330 | *ulaA* | Putative transport protein SgaT | -2.4 | -3.0 | -2.4 | -3.3 | -2.3 | -3.4 | -3.2 | -2.8 |  |  | 2.9 | 3.6 |
| SAUSA300_0331 | *-* | Pts system, IIB component (ec 2.7.1.69) | -4.4 | -3.9 | -3.4 | -4.3 | -4.2 | -4.5 | -4.4 | -4.2 |  |  | 2.4 |  |
| SAUSA300_0332 | *-* | PTS system, mannitol-specific IIA component (EC 2.7.1.69) | -5.1 | -4.6 | -4.1 | -4.6 | -4.9 | -5.6 | -4.8 | -5.5 |  |  | 2.5 |  |
| SAUSA300_0333 | *-* | Transcription antiterminator, BglG family / PTS system, mannitol (Cryptic)-specific IIA component (EC 2.7.1.69) | -5.4 | -5.7 | -4.7 | -6.0 | -5.2 | -6.2 | -6.2 | -6.2 |  |  |  |  |
| SAUSA300_0334 | *-* | Transcriptional regulator, MarR family | 2.1 |  |  |  |  |  |  |  |  |  |  |  |
| SAUSA300_0336 | *-* | Hypothetical cytosolic protein | 2.2 |  |  |  |  |  |  |  |  |  |  |  |
| SAUSA300_0337 | *glpT* | Glycerol-3-phosphate transporter |  |  |  |  |  | -2.0 |  |  |  |  |  |  |
| SAUSA300_0338 | *-* | Hypothetical protein | -2.4 |  |  | -2.0 | -2.8 | -2.6 | -2.6 | -2.1 |  |  |  |  |
| SAUSA300_0339 | *-* | Luciferase-like monooxygenase (EC 1.14.-.-) | -2.4 |  |  |  | -2.9 | -2.5 | -2.3 |  |  | 2.4 | 2.3 |  |
| SAUSA300_0340 | *-* | FMN reductase (EC 1.5.1.29) | -2.0 |  |  |  | -2.8 | -2.2 | -2.2 |  |  | 3.3 | 3.2 | 2.2 |
| SAUSA300_0341 | *-* | Hypothetical membrane spanning protein |  | 2.5 | 2.1 | 2.9 |  |  |  |  |  |  |  |  |
| SAUSA300_0342 | *-* | Hypothetical protein |  |  |  |  |  |  |  |  |  |  |  |  |
| SAUSA300_0343 | *-* | Ribosomal-protein-serine acetyltransferase (EC 2.3.1.-) |  | 2.8 |  | 4.7 | 2.3 | 4.3 | 6.2 | 14.8 |  | 3.5 | 4.9 | 3.8 |
| SAUSA300_0344 | *-* | Lipoprotein |  |  |  |  |  |  |  | 4.9 |  |  |  |  |
| SAUSA300_0345 | *-* | Dyp-type peroxidase family protein |  |  |  |  |  |  |  | 3.1 |  |  |  |  |
| SAUSA300_0347 | *tatC* | Sec-independent protein translocase protein TatC | -2.3 |  | -2.9 |  |  | -2.0 |  |  |  |  |  |  |
| SAUSA300_0348 | *-* | Sec-independent protein translocase protein TatA | -2.4 |  | -3.8 |  | -2.1 | -2.2 | -2.1 |  | -2.4 |  |  | -2.2 |
| SAUSA300_0349 | *-* | Sec-independent protein translocase protein TatA | -2.8 | -2.1 | -7.6 |  | -4.7 | -4.0 | -5.0 | -3.9 | -3.5 | -2.1 |  | -2.1 |
| SAUSA300_0350 | *-* | Transcriptional regulator, Cro/CI family | 3.1 | 3.4 | 3.3 | 2.4 | 2.9 | 2.8 | 2.5 | 2.4 | 2.7 | 2.8 | 2.4 | 2.1 |
| SAUSA300_0351 | *-* | ABC transporter permease protein | 2.9 | 3.6 | 3.1 | 2.1 | 2.8 | 2.3 | 2.5 | 2.3 | 3.1 | 3.1 | 3.0 | 2.5 |
| SAUSA300_0352 | *-* | ABC transporter ATP-binding protein | 2.7 | 3.6 | 3.1 | 2.2 | 2.6 | 2.3 | 2.4 | 2.4 | 2.8 | 3.3 | 3.0 | 2.5 |
| SAUSA300_0354 | *ltrA* | Low temperature requirement protein A |  |  | -2.5 |  |  |  |  |  |  | -2.1 | -2.8 | -3.0 |
| SAUSA300_0355 | *-* | Acetyl-CoA acetyltransferase (EC 2.3.1.9) |  |  | 2.4 |  |  |  |  |  |  |  |  |  |
| SAUSA300_0356 | *-* | Polyketide cyclase family protein | -2.8 |  |  |  | -2.1 | 2.1 | 5.1 | 15.8 |  |  | 2.2 | 2.5 |
| SAUSA300_0357 | *metE* | 5-methyltetrahydropteroyltriglutamate--homocysteine methyltransferase (EC 2.1.1.14) | -6.0 | -3.5 | -5.1 | -4.1 | -3.4 | 2.4 | 5.6 | 15.3 |  |  |  | 2.2 |
| SAUSA300_0358 | *-* | 5-methyltetrahydrofolate--homocysteine methyltransferase (EC 2.1.1.13) homocysteine-binding subunit / Methylenetetrahydrofolate reductase (EC 1.5.1.20) | -2.9 |  | -2.5 | -2.2 |  | 4.8 | 15.0 | 27.6 |  | 2.7 | 3.7 | 5.0 |
| SAUSA300_0359 | *-* | Cystathionine beta-lyase (EC 4.4.1.8) |  |  |  |  |  | 4.1 | 14.0 | 23.3 | 3.1 | 3.5 | 4.6 | 7.1 |
| SAUSA300_0360 | *-* | Cystathionine gamma-synthase (EC 2.5.1.48) |  |  |  |  |  | 7.8 | 26.6 | 41.3 | 2.8 | 3.7 | 5.3 | 6.2 |
| SAUSA300_0361 | *-* | Chromosome partitioning protein parB |  | 2.3 |  | 2.9 | 2.2 | 2.8 | 2.7 | 2.9 | 2.1 |  |  |  |
| SAUSA300_0362 | *-* | Mechanosensitive ion channel |  |  |  |  |  |  |  |  |  | -2.0 | -2.2 | -2.4 |
| SAUSA300_0365 | *-* | Hypothetical protein |  | -2.2 | -2.0 | -2.6 |  | -3.0 | -3.1 | -3.1 | -2.8 | -3.4 | -2.6 | -3.1 |
| SAUSA300_0370 | *-* | Exotoxin | -2.3 |  |  | 2.2 |  | 2.1 | 4.0 | 5.8 |  | 4.0 | 8.8 | 8.1 |
| SAUSA300_0371 | *-* | Hypothetical protein |  |  |  |  |  |  |  |  |  |  | 2.3 | 2.8 |
| SAUSA300_0372 | *-* | Hypothetical exported protein | -4.8 |  |  |  | -26.5 | -4.6 | -3.9 | -3.0 | -4.0 | -3.4 |  |  |
| SAUSA300_0373 | *-* | Hypothetical protein |  |  |  | 2.7 |  | 2.3 | 2.4 | 2.6 |  |  |  |  |
| SAUSA300_0374 | *-* | Hypothetical membrane spanning protein |  |  |  |  | -14.7 | -3.6 | -3.4 | -3.4 | -3.9 | -3.5 | -2.2 |  |
| SAUSA300_0375 | *-* | Phosphoglycerate mutase family protein | -3.2 | -3.0 | -3.0 | -2.7 | -4.3 | -3.3 | -3.7 | -3.5 | -3.7 | -4.8 | -5.4 | -4.7 |
| SAUSA300_0379 | *ahpF* | Peroxiredoxin reductase (NAD(P)H) (EC 1.8.1.-) / NADH oxidase H2O2-forming (EC 1.6.3.-) |  |  |  |  |  |  |  |  |  | 2.1 | 2.6 | 2.2 |
| SAUSA300_0380 | *ahpC* | Peroxiredoxin (EC 1.11.1.15) |  |  |  |  |  |  |  | 2.1 |  | 2.3 | 3.1 | 2.5 |
| SAUSA300_0381 | *-* | Chromate reductase (EC 1.-.-.-) / NADPH-dependent FMN reductase (EC 1.5.1.-) / Oxygen-insensitive NADPH nitroreductase (EC 1.-.-.-) |  |  |  |  | -2.0 |  |  | -2.2 |  |  |  | -2.4 |
| SAUSA300_0382 | *-* | Sodium:dicarboxylate symporter family protein VhcL | -4.7 |  |  | 2.8 | -8.5 | -7.9 | -4.5 | -2.6 | -3.9 | -3.3 | -3.3 | -2.8 |
| SAUSA300_0383 | *-* | Hypothetical protein | 2.1 | 2.2 |  |  | 2.0 |  |  |  |  |  |  |  |
| SAUSA300_0385 | *-* | Transcriptional regulator | -3.6 |  |  |  | -4.4 | -3.1 | -3.0 | -3.5 |  |  |  |  |
| SAUSA300_0386 | *xpt* | Xanthine phosphoribosyltransferase (EC 2.4.2.-) |  |  |  | 2.2 |  |  | 2.2 | 3.5 |  |  |  |  |
| SAUSA300_0387 | *pbuX* | Xanthine permease |  |  | -2.2 |  |  |  |  | 2.2 |  |  |  |  |
| SAUSA300_0393 | *-* | Hypothetical membrane spanning protein |  |  | 2.1 | 2.3 |  |  |  |  | 2.0 | 2.7 | 2.5 |  |
| SAUSA300_0394 | *-* | putative nucleoside-diphosphate-sugar epimerases |  |  |  |  |  |  |  |  | 2.3 | 2.9 | 2.4 |  |
| SAUSA300_0395 | *-* | Exotoxin | -3.3 | -2.6 |  | -2.9 | -4.1 | -3.0 | -2.2 | -2.2 | -3.2 | -2.9 |  |  |
| SAUSA300_0396 | *set7* | Exotoxin |  |  |  |  |  |  |  | 2.1 |  | 2.6 | 4.7 | 4.3 |
| SAUSA300_0397 | *-* | Exotoxin |  |  |  |  |  |  |  |  |  |  |  | 2.4 |
| SAUSA300_0398 | *-* | Exotoxin |  |  |  |  |  |  |  |  |  |  | 2.5 | 3.6 |
| SAUSA300_0401 | *-* | Exotoxin |  |  |  |  |  |  |  |  |  |  | 4.4 | 7.1 |
| SAUSA300_0402 | *-* | Exotoxin |  |  |  |  |  |  |  | 2.4 |  | 2.4 | 4.6 | 3.8 |
| SAUSA300_0403 | *-* | Exotoxin |  |  |  |  |  |  |  |  |  | 2.1 | 3.4 | 3.3 |
| SAUSA300_0404 | *-* | Exotoxin |  |  |  |  |  |  |  |  |  |  | 2.8 | 2.6 |
| SAUSA300_0405 | *hsdM* | Type I restriction-modification system methylation subunit |  |  |  | 2.0 |  |  |  | 2.8 |  |  |  |  |
| SAUSA300_0407 | *-* | Exotoxin | 16.9 | 7.6 | 20.1 | 6.3 | 26.0 | 7.4 | 8.9 | 2.4 | 3.7 | 3.9 | 12.0 | 14.9 |
| SAUSA300_0408 | *-* | Hypothetical cytosolic protein | 12.9 | 8.1 | 18.5 | 5.0 | 18.8 | 5.3 | 6.8 | 2.1 | 2.8 | 3.1 | 7.6 | 10.3 |
| SAUSA300_0409 | *-* | Hypothetical exported protein | -5.3 | -3.3 | -3.0 |  | -2.5 |  |  |  | 2.3 | 3.4 | 4.3 | 4.4 |
| SAUSA300_0410 | *-* | membrane lipoprotein | 2.1 |  | 2.1 | 2.3 | 2.9 | 2.3 |  |  |  |  |  |  |
| SAUSA300_0411 | *-* | membrane lipoprotein | 2.0 |  | 2.5 | 2.2 | 2.3 | 2.1 |  |  |  |  |  |  |
| SAUSA300_0419 | *-* | membrane lipoprotein |  |  | 2.2 |  |  |  |  | -2.1 |  |  |  |  |
| SAUSA300_0420 | *-* | Hypothetical cytosolic protein |  |  | 2.1 | 2.0 |  |  |  |  |  |  | -2.1 | -2.6 |
| SAUSA300_0422 | *-* | Hypothetical protein |  |  |  |  |  |  |  | -2.2 |  |  |  | -2.0 |
| SAUSA300_0424 | *-* | Low-affinity zinc transport protein | 2.3 |  |  | -2.8 |  | -2.9 | -3.1 | -2.9 | -2.0 | -3.0 | -3.1 |  |
| SAUSA300_0425 | *-* | NADH-quinone oxidoreductase chain L (EC 1.6.5.3) |  |  | -6.9 | -16.3 | -3.0 | -9.6 | -19.5 | -23.1 | -4.3 | -9.8 | -19.2 | -23.2 |
| SAUSA300_0426 | *-* | Hypothetical protein |  |  | -3.2 | -7.2 | -2.5 | -4.1 | -8.2 | -10.0 | -3.3 | -6.3 | -9.0 | -8.6 |
| SAUSA300_0427 | *-* | Hypothetical cytosolic protein |  |  |  |  |  |  |  | -2.2 |  |  |  | -2.0 |
| SAUSA300_0429 | *-* | Phosphatidylglycerophosphatase B homolog |  |  |  |  |  |  |  |  |  |  |  | -2.1 |
| SAUSA300_0431 | *-* | Hypothetical protein |  | -3.9 | -2.0 | -4.6 |  | -3.5 | -3.8 | -5.3 | -2.8 | -4.4 | -4.0 | -2.4 |
| SAUSA300_0432 | *-* | Sodium/neurotransmitter symporter family protein | 6.9 | 2.4 |  |  | 2.6 |  |  | -2.2 |  |  |  |  |
| SAUSA300_0433 | *cysM* | Cysteine synthase (EC 2.5.1.47) |  |  | 2.6 | 2.2 | -3.3 | -4.1 | -3.1 | -3.0 |  |  | -2.0 |  |
| SAUSA300_0434 | *metB* | Cystathionine beta-lyase (EC 4.4.1.8) / Cystathionine gamma-lyase (EC 4.4.1.1) |  |  | 3.0 | 2.0 | -3.7 | -4.4 | -3.5 | -2.5 | -2.3 |  | -2.1 | -2.0 |
| SAUSA300_0435 | *-* | ABC transporter ATP-binding protein | -3.1 |  |  |  | -3.1 |  | 2.8 | 8.2 |  |  |  |  |
| SAUSA300_0436 | *-* | ABC transporter permease protein | -5.8 |  | -2.3 |  | -6.5 |  |  | 4.8 | -2.4 |  |  |  |
| SAUSA300_0437 | *-* | ABC transporter substrate-binding protein | -6.5 | -3.2 | -3.8 |  | -9.5 | -2.9 |  |  | -4.5 | -2.2 | -2.0 | -2.1 |
| SAUSA300_0438 | *-* | Putative endopeptidase LytE precursor | 5.5 | 3.7 | 3.0 | 7.2 | 3.6 | 7.9 | 9.7 | 15.1 | 3.1 | 4.1 | 3.9 | 4.2 |
| SAUSA300_0442 | *-* | Hypothetical membrane spanning protein |  |  |  |  | -2.1 |  |  |  |  |  |  |  |
| SAUSA300_0443 | *-* | Hypothetical membrane spanning protein |  | 2.5 |  | 3.7 |  | 2.2 | 2.4 | 3.2 |  |  | 2.5 | 2.4 |
| SAUSA300_0445 | *gltB* | Glutamate synthase [NADPH] large chain (EC 1.4.1.13) |  |  |  | 2.6 | 3.2 | 8.8 | 11.9 | 36.7 | 3.8 | 11.1 | 18.1 | 12.7 |
| SAUSA300_0446 | *gltD* | Glutamate synthase [NADPH] small chain (EC 1.4.1.13) |  |  | -2.6 |  |  | 2.9 | 4.3 | 10.5 |  | 3.7 | 7.6 | 6.1 |
| SAUSA300_0448 | *treP* | PTS system, trehalose-specific IIBC component (EC 2.7.1.69) |  |  | 2.3 |  | -13.8 | -26.6 | -13.4 | -12.2 | -17.2 | -15.0 | -9.9 | -8.9 |
| SAUSA300_0449 | *treC* | Trehalose-6-phosphate hydrolase (EC 3.2.1.93) | -2.0 | -3.2 |  | -2.2 | -7.4 | -13.7 | -12.0 | -10.9 | -12.4 | -14.4 | -11.8 | -9.6 |
| SAUSA300_0450 | *treR* | Trehalose operon transcriptional repressor |  | -3.1 |  | -2.3 | -3.9 | -6.2 | -6.3 | -6.4 | -5.1 | -6.1 | -5.9 | -5.4 |
| SAUSA300_0451 | *-* | Acetyltransferase (EC 2.3.1.-) |  |  |  |  |  |  |  |  |  |  | -2.1 |  |
| SAUSA300_0452 | *dnaX* | DNA polymerase III subunit gamma/tau (EC 2.7.7.7) |  |  |  |  |  |  |  |  |  |  | -2.2 | -2.0 |
| SAUSA300_0464 | *-* | Methyltransferase (EC 2.1.1.-) |  | -2.4 |  | -2.4 |  |  | -2.2 | -2.9 |  | -2.4 | -2.6 | -2.4 |
| SAUSA300_0465 | *-* | Hypothetical protein with endo/excinuclease domain |  |  |  | -2.1 |  |  | -2.0 | -2.4 |  | -2.2 | -3.0 | -2.8 |
| SAUSA300_0466 | *-* | Tetrapyrrole (Corrin/Porphyrin) methylase family protein |  |  |  | -2.5 |  |  | -2.2 | -2.5 | -2.2 | -2.5 | -2.6 | -2.9 |
| SAUSA300_0469 | *-* | Ribonuclease M5 (EC 3.1.26.8) |  |  |  |  |  |  |  |  |  |  |  | -2.3 |
| SAUSA300_0470 | *ksgA* | Dimethyladenosine transferase (EC 2.1.1.-) |  |  |  |  |  |  |  |  |  |  | -2.0 | -2.1 |
| SAUSA300_0471 | *-* | VEG protein |  | -2.1 |  |  |  |  |  |  |  |  |  |  |
| SAUSA300_0472 | *ipk* | 4-diphosphocytidyl-2-C-methyl-D-erythritol kinase (EC 2.7.1.148) | -2.2 | -2.3 | -3.3 |  |  | -2.9 | -2.3 |  | -2.7 | -2.8 | -2.3 |  |
| SAUSA300_0473 | *purR* | Pur operon repressor |  |  | -2.0 |  |  | -2.4 | -2.1 |  |  |  |  |  |
| SAUSA300_0474 | *-* | Translation initiation inhibitor | -4.5 | -2.8 | -2.2 | -2.8 | -6.1 | -8.0 | -6.0 | -4.5 | -6.1 | -5.1 | -2.9 | -2.5 |
| SAUSA300_0475 | *-* | Stage V sporulation protein G | -3.9 | -2.4 |  | -2.1 | -5.1 | -6.7 | -4.9 | -3.6 | -6.0 | -5.0 | -2.9 | -2.8 |
| SAUSA300_0476 | *-* | Hypothetical protein | -8.0 | -11.4 | -10.9 | -12.0 | -9.3 | -14.3 | -13.7 | -13.0 | -9.0 | -10.9 | -4.3 | -3.0 |
| SAUSA300_0478 | *prs* | Ribose-phosphate pyrophosphokinase (EC 2.7.6.1) |  |  |  |  |  |  |  |  | 2.6 | 2.0 |  |  |
| SAUSA300_0490 | *hslO* | 33 kDa chaperonin |  |  | -2.1 | -2.0 |  | -2.6 | -2.9 | -3.1 | -4.0 | -2.6 | -2.4 | -2.5 |
| SAUSA300_0491 | *cysK* | Cysteine synthase (EC 2.5.1.47) | -2.3 |  |  | 2.4 | -3.2 | -3.3 | -2.3 |  | -2.5 |  |  |  |
| SAUSA300_0503 | *-* | Transcriptional regulator, gntr family / aminotransferase class-i (ec 2.6.1.-) |  | 4.9 | 4.0 | 3.7 |  | 3.2 | 3.7 | 2.7 |  | 2.5 | 2.2 | 2.0 |
| SAUSA300_0504 | *-* | pyridoxine biosynthesis protein |  | 3.4 | 3.2 | 3.2 |  | 2.7 | 3.0 | 2.7 |  | 2.3 |  |  |
| SAUSA300_0505 | *-* | pyridoxine biosynthesis amidotransferase (EC 2.4.2.-) |  | 2.9 | 3.2 | 3.2 |  | 2.1 | 2.6 | 2.3 |  |  |  |  |
| SAUSA300_0506 | *nupC* | Nucleoside permease NupC | 2.9 | 3.1 | 3.1 | 2.8 | 3.5 | 2.9 | 3.3 | 3.3 | 3.3 | 2.9 | 2.1 | 2.1 |
| SAUSA300_0507 | *ctsR* | Transcriptional regulator CtsR |  |  | 2.1 |  |  |  |  |  |  | 2.2 |  |  |
| SAUSA300_0508 | *-* | Clpc atpase |  | 2.4 | 2.5 |  |  |  |  |  | 2.6 | 3.5 | 3.0 | 2.7 |
| SAUSA300_0509 | *-* | Arginine kinase (EC 2.7.3.3) |  | 2.8 | 3.0 |  |  |  |  |  | 3.1 | 3.7 | 3.4 | 3.2 |
| SAUSA300_0510 | *clpC* | Negative regulator of genetic competence *clpC*/*mecB* / Hemolysin *tlyB* |  | 2.4 | 2.5 |  |  |  |  |  | 2.2 | 3.2 | 3.4 | 2.8 |
| SAUSA300_0511 | *radA* | DNA repair protein RadA |  |  |  |  |  |  |  | 2.1 |  |  |  |  |
| SAUSA300_0525 | *rplL* | LSU ribosomal protein L12P (L7/L12) | 2.1 |  |  |  | 2.1 |  |  |  |  |  |  |  |
| SAUSA300_0526 | *-* | 16S rRNA m(2)G 1207 methyltransferase (EC 2.1.1.52) |  |  |  | -3.3 |  | -2.0 | -2.1 | -3.1 |  |  |  | -2.2 |
| SAUSA300_0527 | *rpoB* | DNA-directed RNA polymerase beta chain (EC 2.7.7.6) |  |  |  | -2.3 |  |  | -2.0 | -2.2 |  |  |  |  |
| SAUSA300_0530 | *rpsL* | SSU ribosomal protein S12P |  |  |  | -2.4 |  |  |  |  |  |  |  |  |
| SAUSA300_0531 | *-* | SSU ribosomal protein S7P |  |  |  | -2.2 |  |  |  |  |  |  |  |  |
| SAUSA300_0536 | *-* | ThiJ/PfpI family protein | -4.1 |  |  |  | -10.8 | -5.2 | -4.8 | -4.4 | -3.5 |  | -2.4 | -3.2 |
| SAUSA300_0537 | *-* | L-ribulokinase (EC 2.7.1.16) |  |  |  |  |  |  |  | 2.1 |  |  |  | 2.1 |
| SAUSA300_0538 | *-* | L-threonine 3-dehydrogenase (EC 1.1.1.103) |  |  |  | -4.6 |  | -4.2 | -4.2 | -4.8 |  | -2.3 | -2.5 | -2.9 |
| SAUSA300_0539 | *ilvE* | Branched-chain amino acid aminotransferase (EC 2.6.1.42) |  |  |  |  |  |  | 2.1 | 3.7 |  |  |  |  |
| SAUSA300_0540 | *-* | Putative phosphatase |  |  |  | 2.5 |  | 2.9 | 3.5 | 7.0 |  |  | 2.2 | 2.0 |
| SAUSA300_0541 | *-* | Deoxyadenosine kinase (EC 2.7.1.76) / Deoxycytidine kinase (EC 2.7.1.74) | 2.1 |  |  |  |  |  |  |  |  |  |  |  |
| SAUSA300_0542 | *-* | Deoxyguanosine kinase (EC 2.7.1.113) | 2.2 |  |  |  |  |  |  |  |  |  |  |  |
| SAUSA300_0544 | *-* | Hydrolase (HAD superfamily) | 2.2 |  |  |  |  | 2.3 | 2.3 | 2.8 |  |  |  |  |
| SAUSA300_0545 | *-* | Acyl carrier protein phosphodiesterase (EC 3.1.4.14) | 2.1 |  |  |  |  |  |  |  |  |  |  |  |
| SAUSA300_0546 | *sdrC* | Fibronectin-binding protein | -3.7 | -3.5 | -4.0 | -2.8 | -2.9 | -2.5 | -2.4 | -2.3 | -2.5 | -2.5 | -2.6 | -2.0 |
| SAUSA300_0547 | *sdrD* | Fibronectin-binding protein | -2.6 |  | -2.4 |  |  |  |  |  |  |  |  |  |
| SAUSA300_0548 | *sdrE* | Fibronectin-binding protein |  |  |  |  |  |  |  |  |  |  | -2.3 |  |
| SAUSA300_0549 | *-* | Poly(Glycerol-phosphate) alpha-glucosyltransferase (EC 2.4.1.52) |  | 2.5 | 2.6 | 2.5 |  | 2.3 | 2.3 | 2.4 |  |  |  |  |
| SAUSA300_0552 | *-* | GlcNAc-PI de-N-acetylase family protein |  |  | -2.4 |  |  |  |  |  |  |  |  | -2.1 |
| SAUSA300_0553 | *-* | Hypothetical protein |  |  | -3.9 |  |  | -2.1 | -2.1 |  |  |  | -2.0 | -2.3 |
| SAUSA300_0554 | *-* | Glucosamine-6-phosphate isomerase (EC 3.5.99.6) |  |  | 2.0 | 2.8 |  | 2.1 | 2.3 | 2.2 |  |  |  |  |
| SAUSA300_0555 | *-* | Hexulose-6-phosphate synthase (EC 4.1.2.-) | -2.2 |  |  |  | -2.8 |  |  | -2.2 |  |  |  |  |
| SAUSA300_0556 | *-* | Hexulose-6-phosphate isomerase (EC 5.-.-.-) | -2.2 |  |  |  | -2.7 |  |  | -2.0 |  |  |  |  |
| SAUSA300_0557 | *-* | Putative phosphatase |  |  |  | 2.3 | 2.2 | 5.8 | 5.2 | 5.9 | 2.8 | 4.0 | 3.1 | 3.3 |
| SAUSA300_0558 | *-* | Proline/betaine transporter | -3.0 | -2.0 |  | -3.3 | -8.4 | -6.9 | -7.1 | -7.4 | -7.2 | -4.7 | -3.2 | -3.5 |
| SAUSA300_0562 | *thiD* | Phosphomethylpyrimidine kinase (EC 2.7.4.7) / Hydroxymethylpyrimidine kinase (EC 2.7.1.49) |  |  |  | 2.3 |  |  |  |  |  |  |  |  |
| SAUSA300_0566 | *-* | Arginine permease | -3.9 | -4.7 | -5.4 | -6.3 | -7.6 | -10.9 | -10.2 | -10.6 | -11.6 | -10.8 | -7.8 | -7.4 |
| SAUSA300_0572 | *mvk* | Mevalonate kinase (EC 2.7.1.36) | -2.1 | -3.7 | -2.1 | -4.9 | -2.2 | -3.9 | -5.2 | -8.3 | -4.2 | -5.8 | -5.0 | -4.6 |
| SAUSA300_0573 | *mvaD* | Diphosphomevalonate decarboxylase (EC 4.1.1.33) | -2.5 | -3.4 |  | -5.1 | -2.6 | -5.1 | -6.5 | -10.4 | -3.7 | -5.5 | -5.6 | -4.7 |
| SAUSA300_0574 | *-* | Phosphomevalonate kinase (EC 2.7.4.2) | -2.1 | -2.6 |  | -4.8 | -2.5 | -5.5 | -7.1 | -14.4 | -3.6 | -5.3 | -5.4 | -4.8 |
| SAUSA300_0575 | *-* | Hypothetical cytosolic protein |  | -5.8 | -3.8 | -4.5 |  | -4.4 | -4.3 | -3.3 | -4.6 | -4.1 | -3.1 | -2.6 |
| SAUSA300_0576 | *-* | Pyridine nucleotide-disulphide oxidoreductase family protein (EC 1.-.-.-) |  | 3.5 |  |  |  |  |  | 2.0 | 11.4 | 23.3 | 15.3 | 7.9 |
| SAUSA300_0577 | *-* | Rrf2 family protein | 2.7 | 3.8 |  |  |  |  |  | 2.5 | 21.8 | 40.0 | 15.2 | 5.2 |
| SAUSA300_0579 | *-* | Phage protein | 2.9 | 3.7 | 4.1 | 4.4 | 2.8 |  |  |  | 2.4 |  |  |  |
| SAUSA300_0580 | *-* | Hypothetical membrane spanning protein |  | 2.3 | 2.5 | 2.7 |  |  |  |  |  |  |  |  |
| SAUSA300_0581 | *-* | Hypothetical membrane spanning protein |  | 2.3 | 2.5 | 2.7 |  |  |  |  |  |  |  |  |
| SAUSA300_0582 | *-* | Hypothetical membrane spanning protein |  | 2.3 | 2.1 | 2.2 |  |  |  |  |  |  |  |  |
| SAUSA300_0591 | *-* | Hypothetical protein | 2.1 | 2.7 |  | 3.9 | 2.3 | 3.1 | 2.7 | 2.8 | 2.3 | 2.5 | 2.0 |  |
| SAUSA300_0592 | *-* | dGTP triphosphohydrolase |  |  |  | 2.1 |  |  |  |  |  |  |  |  |
| SAUSA300_0594 | *adh* | Alcohol dehydrogenase (EC 1.1.1.1) | -10.1 |  | 4.5 | 7.3 | -5.1 | 2.4 | 4.4 | 5.7 | -8.0 | -5.2 | 4.0 | 8.6 |
| SAUSA300_0595 | *-* | Hypothetical protein | 2.3 |  |  |  | 2.0 |  |  |  |  |  |  |  |
| SAUSA300_0598 | *-* | Cobalamin-binding protein | 2.4 |  |  | 2.1 | 3.2 | 2.3 | 2.2 | 3.0 |  |  |  |  |
| SAUSA300_0599 | *-* | Transporter |  | 2.1 |  | 2.4 |  | 2.3 | 2.2 | 2.6 |  |  |  |  |
| SAUSA300_0600 | *-* | Hydrolase (HAD superfamily) | 2.6 | 3.3 | 2.5 | 3.3 | 2.6 | 2.8 | 2.9 | 2.7 | 2.7 | 2.5 |  |  |
| SAUSA300_0601 | *-* | 3-oxoadipate enol-lactonase (EC 3.1.1.24) | 2.5 | 3.5 | 3.1 | 3.3 | 2.7 | 2.9 | 2.6 | 2.4 | 2.5 | 2.5 |  |  |
| SAUSA300_0603 | *-* | Hypothetical protein |  | -3.6 | -3.2 | -3.0 | -2.3 | -4.1 | -4.5 | -4.5 | -3.5 | -3.5 | -3.1 | -2.7 |
| SAUSA300_0604 | *-* | Esterase (EC 3.1.1.-) | -3.1 |  |  | -3.2 | -25.7 | -6.9 | -5.5 | -5.4 | -3.5 | -3.1 |  |  |
| SAUSA300_0605 | *sarA* | Staphylococcal accessory regulator A |  |  |  | -2.1 |  | -2.3 | -2.0 | -2.1 |  |  |  |  |
| SAUSA300_0608 | *-* | Hypothetical protein |  |  |  |  |  |  |  |  |  |  |  | 2.4 |
| SAUSA300_0610 | *-* | Sodium/proton antiporter protein ShaA |  |  |  |  |  |  |  |  |  |  | 2.0 | 3.1 |
| SAUSA300_0611 | *-* | Sodium/proton antiporter protein ShaB |  |  |  |  |  |  |  |  |  |  |  | 2.4 |
| SAUSA300_0614 | *-* | Sodium/proton antiporter protein ShaE |  |  |  |  | -2.2 | -2.0 |  |  |  |  |  |  |
| SAUSA300_0615 | *-* | Sodium/proton antiporter protein ShaF |  |  |  |  | -2.0 | -2.1 |  |  |  |  |  |  |
| SAUSA300_0616 | *-* | Sodium/proton antiporter protein ShaG |  |  |  |  | -2.0 |  |  |  |  |  |  |  |
| SAUSA300_0618 | *-* | Manganese-binding protein | -10.6 | -2.1 |  |  |  |  |  |  |  |  |  |  |
| SAUSA300_0619 | *-* | Manganese transport system membrane protein | -9.6 | -2.1 |  |  |  |  |  |  |  | 2.0 |  |  |
| SAUSA300_0620 | *-* | Manganese transport system ATP-binding protein | -12.6 | -2.9 | -2.1 |  |  |  |  |  |  |  |  |  |
| SAUSA300_0621 | *-* | Iron-dependent repressor | -2.2 | -2.2 | -2.2 | -3.3 |  |  |  |  |  |  |  |  |
| SAUSA300_0629 | *pbp4* | Hypothetical protein |  | -2.1 | -4.9 | -3.2 |  | -3.6 | -4.4 | -3.9 | -3.8 | -3.8 | -3.8 | -4.7 |
| SAUSA300_0630 | *-* | Multidrug resistance ABC transporter ATP-binding and permease protein | -2.9 |  |  |  | -3.6 | -3.0 | -2.8 | -2.5 | -2.9 | -2.8 | -2.6 | -2.4 |
| SAUSA300_0633 | *fhuA* | Ferrichrome transport ATP-binding protein fhuC |  |  |  | -4.2 | 2.9 | 4.0 | 4.3 | 4.8 | 2.9 | 3.4 | 2.8 | 2.9 |
| SAUSA300_0634 | *fhuB* | Ferrichrome transport system permease protein fhuB |  |  | -2.7 | -3.7 | 2.2 | 2.4 | 2.5 | 3.6 | 2.2 | 2.4 | 2.1 | 2.3 |
| SAUSA300_0635 | *fhuG* | Ferrichrome transport system permease protein FhuG |  |  | -2.0 | -2.4 |  |  | 2.2 | 2.8 |  |  |  |  |
| SAUSA300_0636 | *-* | Dihydroxyacetone kinase (EC 2.7.1.29) |  | 2.4 | 4.2 |  |  |  |  |  | 5.2 | 6.9 | 7.0 | 5.5 |
| SAUSA300_0637 | *-* | Dihydroxyacetone kinase (EC 2.7.1.29) |  |  | 3.0 |  |  |  |  |  | 3.0 | 3.7 | 4.1 | 3.4 |
| SAUSA300_0638 | *-* | Dihydroxyacetone kinase phosphotransfer protein |  |  | 2.2 |  |  |  |  |  | 2.1 | 3.3 | 3.2 | 2.7 |
| SAUSA300_0639 | *-* | Hypothetical protein |  |  |  |  |  |  |  | -2.2 |  |  |  |  |
| SAUSA300_0640 | *-* | Hypothetical membrane spanning protein |  |  |  | -2.0 |  | -2.2 | -2.4 | -2.4 |  |  |  |  |
| SAUSA300_0641 | *-* | Lipase (EC 3.1.1.3) |  |  | -3.4 |  |  |  |  |  | -2.6 |  |  |  |
| SAUSA300_0642 | *-* | Hypothetical protein |  |  |  |  |  |  |  |  | -2.4 |  |  | -2.1 |
| SAUSA300_0643 | *-* | Acetyltransferase, GNAT family |  |  |  |  |  | -2.5 | -3.3 | -4.6 |  |  |  | -2.1 |
| SAUSA300_0649 | *-* | Putative pit accessory protein | 2.5 |  |  |  |  |  |  |  |  |  |  |  |
| SAUSA300_0651 | *-* | Secretory antigen precursor SsaA | 4.1 | 2.4 | 2.2 | 4.5 | 2.9 | 5.8 | 6.0 | 8.7 | 4.8 | 5.8 | 5.1 | 5.5 |
| SAUSA300_0653 | *-* | Transcriptional regulator, AraC family |  |  |  |  |  |  |  |  | -2.1 |  |  |  |
| SAUSA300_0654 | *sarX* | Staphylococcal accessory regulator a |  |  |  |  |  | -2.0 |  |  | -2.1 | -2.0 | -2.1 |  |
| SAUSA300_0661 | *-* | Hypothetical membrane spanning protein |  |  | -2.1 |  |  |  |  |  |  |  |  |  |
| SAUSA300_0663 | *-* | Hypothetical exported protein |  |  |  |  |  |  |  |  | -2.3 | -2.7 | -2.9 | -2.6 |
| SAUSA300_0664 | *-* | Hypothetical protein | -2.5 |  |  | -2.0 | -4.1 | -3.7 | -3.7 | -3.9 | -4.1 | -4.3 | -2.8 | -2.4 |
| SAUSA300_0665 | *-* | Ribosomal-protein-alanine acetyltransferase (EC 2.3.1.128) | -2.6 | -3.1 |  | -3.2 | -3.0 | -4.3 | -4.6 | -5.4 | -5.8 | -6.3 | -4.2 | -3.5 |
| SAUSA300_0666 | *-* | Lysine decarboxylase family | -2.8 | -2.3 |  | -2.8 | -2.7 | -3.2 | -3.2 | -3.6 | -2.2 | -2.5 |  |  |
| SAUSA300_0667 | *-* | EMG2 protein | -2.9 | -2.2 | -2.1 | -3.1 | -2.8 | -3.5 | -3.8 | -4.5 | -2.8 | -3.1 | -2.3 | -2.1 |
| SAUSA300_0668 | *-* | Hypothetical protein |  |  |  |  | -3.0 | -2.4 | -2.6 | -3.2 | -2.1 | -2.4 |  |  |
| SAUSA300_0669 | *-* | Undecaprenyl-diphosphatase (EC 3.6.1.27) (Bacitracin resistance protein) | 2.7 | 2.6 | 2.0 |  | 2.5 | 2.4 |  |  |  |  |  |  |
| SAUSA300_0670 | *-* | Transport ATP-binding protein CydD |  |  |  |  | -2.4 |  |  |  |  |  |  |  |
| SAUSA300_0671 | *-* | Hypothetical protein |  |  |  |  | -2.3 |  |  | -2.1 | -2.2 | -2.1 |  |  |
| SAUSA300_0676 | *-* | Transporter, Divalent Anion:Sodium Symporter family |  |  | 2.4 | 2.1 |  |  | 2.0 | 2.3 |  |  |  |  |
| SAUSA300_0678 | *-* | Permease |  |  |  |  |  |  | -2.2 |  |  |  |  |  |
| SAUSA300_0680 | *norA* | Quinolone resistance protein NorA |  |  |  |  |  | -2.1 |  |  | -2.0 |  |  |  |
| SAUSA300_0681 | *-* | Hypothetical protein | 5.0 | 3.0 |  | 3.4 | 3.5 | 4.8 | 5.8 | 7.5 | 3.6 | 4.6 | 4.4 | 5.1 |
| SAUSA300_0682 | *ybaK* | Regulatory protein | 2.6 |  | 2.8 | 2.3 |  |  |  | 2.2 |  |  |  |  |
| SAUSA300_0683 | *-* | Fructose repressor |  | 2.6 | 3.6 |  | -7.6 | -16.7 | -6.7 | -5.5 | -13.2 | -14.4 | -3.6 |  |
| SAUSA300_0684 | *fruB* | 1-phosphofructokinase (EC 2.7.1.56) |  | 2.5 | 3.9 | 2.2 | -7.1 | -18.1 | -7.8 | -5.5 | -12.8 | -17.8 | -3.9 |  |
| SAUSA300_0685 | *fruA* | PTS system, fructose-specific IIABC component (EC 2.7.1.69) |  |  | 3.4 |  | -7.8 | -19.8 | -8.6 | -4.6 | -14.6 | -14.5 | -4.4 |  |
| SAUSA300_0688 | *-* | 2,5-diketo-D-gluconic acid reductase (EC 1.1.1.274) | -3.0 |  |  |  | -3.7 |  | -2.8 | -3.7 |  |  | -2.0 | -2.6 |
| SAUSA300_0689 | *-* | Undecaprenyl-phosphate beta-N-acetylglucosaminyltransferase (EC 2.4.1.-) | -2.6 |  |  |  | -3.7 | -2.1 | -2.6 | -2.7 |  |  |  | -2.4 |
| SAUSA300_0690 | *saeS* | Sensory transduction protein kinase SaeS (EC 2.7.3.-) |  |  |  |  |  |  |  |  |  |  | 2.2 |  |
| SAUSA300_0691 | *saeR* | Two-component response regulator SaeR |  |  |  |  |  |  |  |  |  |  | 2.4 | 2.2 |
| SAUSA300_0692 | *-* | Hypothetical protein |  |  | -2.0 |  |  |  |  |  |  |  | 2.5 | 2.1 |
| SAUSA300_0693 | *-* | Hypothetical protein |  | 2.6 | 3.9 | 6.3 | 3.4 | 3.8 | 5.2 | 5.2 | 8.2 | 9.1 | 10.7 | 9.3 |
| SAUSA300_0694 | *-* | Hypothetical protein |  | -2.1 |  | -2.5 |  | -2.5 | -2.8 | -2.2 | -2.0 | -2.1 |  |  |
| SAUSA300_0695 | *-* | Queuosine biosynthesis protein QueE | 3.1 |  |  |  |  |  |  |  |  |  |  |  |
| SAUSA300_0696 | *-* | 6-pyruvoyl tetrahydropterin synthase (EC 4.2.3.12) | 3.2 |  |  |  |  |  |  | 2.1 |  |  |  |  |
| SAUSA300_0697 | *-* | Queuosine biosynthesis protein QueC | 3.3 |  |  |  |  |  |  | 2.1 |  |  |  |  |
| SAUSA300_0698 | *pabA* | Anthranilate synthase component II (EC 4.1.3.27) / Para-aminobenzoate synthase glutamine amidotransferase component II (EC 6.3.5.8) | 2.1 |  |  | 2.1 | 2.7 | 2.3 | 2.4 | 2.4 | 2.4 | 2.3 | 2.2 | 2.2 |
| SAUSA300_0699 | *-* | Para-aminobenzoate synthetase component I (EC 6.3.5.8) |  |  |  |  | 2.3 | 2.0 | 2.0 | 2.1 |  |  |  |  |
| SAUSA300_0700 | *-* | 4-amino-4-deoxychorismate lyase (EC 4.1.3.38) |  |  |  |  | 2.1 |  |  |  |  |  |  |  |
| SAUSA300_0703 | *-* | Sulfatase family protein |  |  |  |  |  |  |  | -2.1 |  |  |  |  |
| SAUSA300_0704 | *-* | ABC transporter ATP-binding protein Uup |  |  | -2.7 |  |  |  |  |  | -2.6 |  |  |  |
| SAUSA300_0705 | *recQ* | ATP-dependent DNA helicase recQ (EC 3.6.1.-) |  |  |  |  |  | -2.2 |  |  | -2.4 |  |  |  |
| SAUSA300_0706 | *-* | Glycine betaine transport ATP-binding protein | -3.2 |  | -3.2 |  | -2.8 |  |  |  |  |  |  |  |
| SAUSA300_0707 | *-* | Glycine betaine transport system permease protein / Glycine betaine-binding protein | -2.8 |  | -2.3 |  | -2.9 | -2.1 |  |  |  |  |  |  |
| SAUSA300_0708 | *hisC* | Histidinol-phosphate aminotransferase (EC 2.6.1.9) | -3.2 |  | -2.8 |  | -2.4 |  | 2.4 | 6.1 |  | 2.0 | 2.8 |  |
| SAUSA300_0709 | *-* | Hydrolase (HAD superfamily) |  |  | -2.4 |  |  |  |  | 2.4 |  |  |  |  |
| SAUSA300_0711 | *-* | Hypothetical protein | -11.0 | -5.5 | -5.5 | -6.6 | -26.1 | -20.4 | -18.4 | -15.2 | -10.2 | -9.7 | -4.3 | -3.7 |
| SAUSA300_0712 | *-* | Di-/tripeptide transporter |  |  |  | 2.2 |  |  |  |  |  |  |  |  |
| SAUSA300_0715 | *nrdI* | NrdI protein | -2.6 |  | -3.1 |  | -2.5 |  |  |  | -2.6 |  |  |  |
| SAUSA300_0716 | *-* | Ribonucleoside-diphosphate reductase alpha chain (EC 1.17.4.1) | -2.8 |  | -2.6 |  | -2.5 |  |  |  |  |  |  |  |
| SAUSA300_0717 | *nrdF* | Ribonucleoside-diphosphate reductase beta chain (EC 1.17.4.1) | -2.6 |  | -3.6 |  | -2.5 |  |  |  | -3.0 | -2.3 | -2.4 | -2.2 |
| SAUSA300_0718 | *sstA* | Ferric anguibactin transport system permease protein fatD | 3.8 | 3.6 | 2.5 |  | 24.3 | 24.7 | 21.6 | 12.6 | 15.4 | 13.0 | 10.0 | 11.7 |
| SAUSA300_0719 | *sstB* | Ferric anguibactin transport system permease protein fatC | 3.5 | 3.4 | 2.7 |  | 19.2 | 17.1 | 15.0 | 8.4 | 13.2 | 9.8 | 7.2 | 7.7 |
| SAUSA300_0720 | *sstC* | Ferric anguibactin transport ATP-binding protein | 4.4 | 3.6 | 2.8 |  | 22.3 | 17.0 | 14.8 | 6.3 | 15.4 | 10.8 | 6.8 | 7.7 |
| SAUSA300_0721 | *sstD* | Ferric anguibactin-binding protein | 6.0 | 6.2 | 5.2 |  | 23.0 | 26.7 | 25.3 | 13.0 | 15.8 | 16.8 | 14.1 | 12.0 |
| SAUSA300_0723 | *-* | Glutamate-rich protein GrpB | 3.5 |  |  |  | 2.8 |  |  |  |  |  |  |  |
| SAUSA300_0725 | *-* | Hypothetical cytosolic protein |  |  | -2.0 |  |  | -2.0 | -2.1 |  | -2.9 | -2.8 | -2.4 | -2.5 |
| SAUSA300_0726 | *-* | Glycerate kinase (EC 2.7.1.31) |  |  |  |  |  |  |  | -2.2 |  |  |  |  |
| SAUSA300_0730 | *-* | Sensory transduction protein kinase (EC 2.7.3.-) |  | -2.1 |  |  |  |  |  | -2.1 |  |  |  |  |
| SAUSA300_0734 | *-* | COMF operon protein 1 |  | -2.2 |  |  |  |  |  | -2.1 |  |  |  |  |
| SAUSA300_0735 | *-* | COMF operon protein 3 |  |  |  | -2.4 |  | -2.1 | -2.2 | -2.1 |  | -2.3 |  |  |
| SAUSA300_0736 | *yfiA* | Ribosome-associated factor Y |  | 2.4 | 3.8 |  |  | 3.0 | 3.5 | 2.6 | 3.0 | 3.5 | 4.2 | 4.8 |
| SAUSA300_0737 | *secA* | Protein translocase subunit SecA |  |  |  |  |  |  |  | -2.1 |  |  |  |  |
| SAUSA300_0740 | *-* | Hydrolase (HAD superfamily) |  |  |  | 2.8 |  |  |  | 2.6 |  |  | 2.1 |  |
| SAUSA300_0747 | *trxB* | Thioredoxin reductase (EC 1.8.1.9) | -2.1 |  |  |  |  |  | -2.1 |  |  |  |  |  |
| SAUSA300_0748 | *-* | ATP-binding protein (contains P-loop) | -2.3 |  | -2.3 |  | -2.3 | -2.1 | -2.2 | -2.0 |  |  |  |  |
| SAUSA300_0749 | *-* | Hypothetical membrane associated protein | -3.1 |  |  | -2.1 | -3.4 | -3.0 | -2.7 | -2.3 | -2.1 | -2.0 |  |  |
| SAUSA300_0750 | *-* | Hypothetical cytosolic protein | -2.3 | -2.1 | -2.0 | -2.5 | -2.6 | -3.0 | -3.1 | -2.9 | -2.4 | -2.2 |  |  |
| SAUSA300_0753 | *-* | Cell division inhibitor | -2.6 |  |  |  | -2.6 | -2.2 | -2.1 |  | -2.0 |  |  |  |
| SAUSA300_0754 | *-* | Hypothetical protein | -2.8 | -2.9 | -3.1 |  | -4.3 | -4.1 | -3.9 | -3.1 | -5.9 | -5.4 | -4.8 | -4.7 |
| SAUSA300_0763 | *est* | Carboxylesterase (EC 3.1.1.1) |  | -3.0 | -3.7 | -4.6 |  | -3.4 | -3.2 | -3.7 | -2.9 | -2.7 | -2.5 | -2.3 |
| SAUSA300_0764 | *rnr* | Exoribonuclease II (EC 3.1.13.1) |  |  | -2.4 | -3.4 |  | -3.2 | -3.0 | -3.1 | -3.4 | -3.2 | -3.0 | -3.7 |
| SAUSA300_0765 | *smpB* | SsrA-binding protein |  |  |  | -2.2 |  | -2.7 | -2.4 | -2.5 | -2.4 | -2.3 | -2.3 | -2.9 |
| SAUSA300_0767 | *-* | Hypothetical protein |  |  |  | 2.1 |  |  |  |  |  |  | -2.1 | -2.5 |
| SAUSA300_0768 | *-* | Hypothetical protein |  |  |  |  |  |  |  |  |  |  | -3.3 | -4.5 |
| SAUSA300_0769 | *-* | Hypothetical exported protein |  |  |  | 2.3 |  |  |  |  |  |  | -3.2 | -4.8 |
| SAUSA300_0770 | *-* | Hypothetical protein |  |  |  |  | -2.7 | -2.7 | -2.5 | -2.2 |  | -2.6 |  |  |
| SAUSA300_0771 | *-* | Acetyltransferase (EC 2.3.1.-) |  |  |  |  |  |  | -2.0 | -2.0 | -2.0 | -2.2 | -2.1 | -2.2 |
| SAUSA300_0772 | *clfA* | Fibronectin-binding protein | -6.2 | -5.4 | -3.5 | -6.9 | -13.4 | -22.2 | -19.2 | -17.8 | -7.7 | -10.2 | -3.9 | -2.9 |
| SAUSA300_0773 | *-* | Staphylocoagulase precursor | 13.1 | 2.4 | 3.0 |  | 26.4 | 5.1 | 6.7 | 3.0 | 4.2 | 4.1 | 7.6 | 7.3 |
| SAUSA300_0774 | *empbp* | Extracellular matrix binding protein / Fibrinogen-binding protein |  | -2.2 |  | 2.3 | 2.2 | 4.0 | 5.8 | 6.3 | 6.5 | 9.7 | 20.4 | 22.6 |
| SAUSA300_0775 | *-* | extracellular matrix and plasma binding protein | 4.2 | 2.5 | 4.7 | 3.2 | 7.2 | 4.0 | 5.7 | 3.3 | 6.2 | 5.1 | 9.5 | 12.9 |
| SAUSA300_0776 | *nuc* | Thermonuclease (EC 3.1.31.1) | -2.4 |  |  | 2.7 |  | 2.4 | 2.4 | 3.2 |  |  |  |  |
| SAUSA300_0777 | *-* | Cold shock protein |  | -2.6 | -2.8 | -2.7 |  | -2.9 | -2.4 | -2.6 | -4.7 | -5.6 | -4.3 | -3.4 |
| SAUSA300_0780 | *-* | Hypothetical exported protein |  |  | -2.9 |  |  |  |  |  |  |  |  |  |
| SAUSA300_0782 | *-* | Hypothetical protein |  |  |  | 2.4 | -2.6 | -2.5 |  |  | -2.3 |  |  |  |
| SAUSA300_0781 | *-* | Hypothetical protein | -2.7 |  |  | -2.0 | -18.8 | -6.3 | -4.2 | -4.0 | -3.6 | -3.6 |  |  |
| SAUSA300_0783 | *-* | Phosphoglycerate mutase family protein |  | -3.6 |  |  |  | -2.1 | -2.1 | -3.1 |  | -2.1 | -2.3 | -2.4 |
| SAUSA300_0784 | *-* | Transporter, LysE family |  |  | -2.5 |  |  | 2.6 | 2.5 | 3.5 |  |  |  |  |
| SAUSA300_0785 | *-* | Acetyltransferase (EC 2.3.1.-) | 2.1 |  | 2.5 | 3.3 |  |  |  | 2.1 |  |  |  |  |
| SAUSA300_0786 | *-* | Organic hydroperoxide resistance protein | -4.8 | -3.0 |  | -2.4 | -11.6 | -6.7 | -4.6 | -4.6 | -6.9 | -7.2 | -3.3 | -2.5 |
| SAUSA300_0787 | *aroD* | 3-dehydroquinate dehydratase (EC 4.2.1.10) |  |  |  |  |  |  |  |  |  |  |  |  |
| SAUSA300_0788 | *-* | Nitroreductase family |  |  |  |  | 2.9 | 4.1 | 3.9 | 4.1 | 2.5 | 2.5 | 2.1 | 2.4 |
| SAUSA300_0790 | *-* | Arsenate reductase family protein |  |  |  | 2.2 |  |  |  |  | 2.2 | 2.1 |  |  |
| SAUSA300_0791 | *gcvH* | Glycine cleavage system H protein |  |  |  |  |  |  |  |  | 2.2 |  |  |  |
| SAUSA300_0793 | *-* | Hypothetical cytosolic protein | 2.1 |  | 5.5 |  | 2.9 | 2.7 |  |  | 2.5 |  |  |  |
| SAUSA300_0794 | *-* | DNA primase (bacterial type) and small primase-like proteins |  |  |  |  |  |  |  | -2.0 |  |  |  |  |
| SAUSA300_0796 | *-* | ABC transporter ATP-binding protein |  |  | -3.2 |  |  |  | 3.6 | 8.3 |  |  |  |  |
| SAUSA300_0797 | *-* | ABC transporter permease protein | -3.7 |  | -6.2 | -2.2 | -2.5 |  | 2.5 | 5.7 |  |  |  |  |
| SAUSA300_0798 | *-* | ABC transporter substrate-binding protein | -3.3 |  | -4.6 |  | -2.5 |  | 2.5 | 5.0 |  |  |  |  |
| SAUSA300_0799 | *int* | DNA integration/recombination/inversion protein | 5.0 |  | 2.0 |  | 3.9 |  |  |  |  |  |  |  |
| SAUSA300_0800 | *sek* | Enterotoxin | 2.8 | 2.3 | 2.2 | 2.1 | 2.6 |  |  |  | 2.1 |  |  |  |
| SAUSA300_0801 | *seq* | Enterotoxin | 2.5 |  |  |  | 2.3 |  |  |  |  |  |  |  |
| SAUSA300_0802 | *-* | Zn-dependent alcohol dehydrogenases and related dehydrogenases | 2.1 |  |  |  |  |  |  |  |  |  |  |  |
| SAUSA300_0803 | *-* | Phage transcriptional repressor | 2.3 |  |  |  |  |  |  |  |  |  |  |  |
| SAUSA300_0804 | *-* | Transcriptional regulator, Cro family |  |  |  |  |  | -2.1 | -2.4 | -2.1 |  | -2.1 |  |  |
| SAUSA300_0805 | *-* | Phage protein |  |  |  |  |  | -2.8 | -2.5 | -2.3 |  | -2.2 | -2.1 | -2.0 |
| SAUSA300_0806 | *-* | Hypothetical protein |  |  |  |  |  | -2.4 | -2.0 |  |  |  |  |  |
| SAUSA300_0807 | *-* | Hypothetical protein |  |  |  |  |  |  |  |  |  |  |  |  |
| SAUSA300_0808 | *-* | Hypothetical protein |  |  |  |  |  | -2.1 |  |  |  |  |  |  |
| SAUSA300_0810 | *-* | Hypothetical protein |  |  |  |  |  |  |  |  | -3.0 | -2.5 | -2.9 | -3.7 |
| SAUSA300_0814 | *-* | Phage-related protein |  |  | -2.4 |  |  |  |  |  | -2.7 | -2.4 | -2.4 | -2.5 |
| SAUSA300_0815 | *ear* | Hypothetical protein | -3.7 | -3.0 | -2.2 |  |  |  |  |  |  | 2.1 | 3.2 | 4.3 |
| SAUSA300_0817 | *-* | Integral membrane protein |  |  |  | -2.0 |  |  |  |  |  |  |  |  |
| SAUSA300_0818 | *sufC* | ATP-dependent transporter sufC | -2.3 | -3.0 | -4.3 | -4.3 | -3.5 | -3.9 | -3.9 | -3.7 | -4.0 | -3.8 | -3.8 | -4.1 |
| SAUSA300_0819 | *sufD* | SufD protein | -2.4 | -2.7 | -4.0 | -4.5 | -3.4 | -4.0 | -4.0 | -3.8 | -3.4 | -3.3 | -3.4 | -3.8 |
| SAUSA300_0820 | *sufS* | Cysteine desulfurase (EC 2.8.1.7) / Selenocysteine lyase (EC 4.4.1.16) | -2.2 | -2.8 | -3.8 | -4.7 | -3.1 | -4.3 | -4.6 | -4.2 | -2.8 | -2.8 | -2.9 | -3.1 |
| SAUSA300_0821 | *-* | IscU protein |  | -2.3 | -3.2 | -3.6 | -2.6 | -3.6 | -3.7 | -3.8 | -2.7 | -2.7 | -2.7 | -3.3 |
| SAUSA300_0822 | *sufB* | ABC transporter-associated protein SufB |  |  | -2.5 | -2.7 | -2.4 | -3.0 | -3.0 | -2.8 | -2.4 | -2.0 |  | -2.4 |
| SAUSA300_0823 | *-* | Hypothetical protein |  |  |  |  |  |  |  | 2.6 |  |  |  |  |
| SAUSA300_0825 | *-* | Nitropropane dioxygenase / Trans-enoyl-CoA reductase family | -2.0 |  |  |  | -2.4 |  | -2.1 |  | -2.3 |  |  | -2.2 |
| SAUSA300_0826 | *-* | Hypothetical cytosolic protein |  |  |  |  |  |  |  | -2.3 |  |  |  |  |
| SAUSA300_0827 | *-* | Hypothetical membrane spanning protein |  |  |  |  |  |  |  | -2.2 |  |  |  |  |
| SAUSA300_0828 | *-* | 5'-nucleotidase (EC 3.1.3.5) |  |  |  |  |  |  |  | -2.1 |  |  |  |  |
| SAUSA300_0832 | *-* | Hypothetical cytosolic protein |  | 2.0 | 2.5 | 2.1 |  |  |  |  | 2.2 |  |  |  |
| SAUSA300_0833 | *-* | Hydrolase (HAD superfamily) |  | 2.1 | 2.2 | 2.0 |  | 2.0 |  |  | 2.5 |  |  |  |
| SAUSA300_0834 | *-* | Glyoxylate reductase (NADP+) (EC 1.1.1.79) / Glyoxylate reductase (NAD+) (EC 1.1.1.26) / Hydroxypyruvate reductase (EC 1.1.1.81) |  | 2.1 | 2.6 | 2.4 |  |  |  |  | 2.2 |  |  |  |
| SAUSA300_0835 | *dltA* | D-alanine-activating enzyme (EC 6.3.2.-) | 2.7 |  |  |  | 3.1 | 2.0 |  |  |  |  |  |  |
| SAUSA300_0836 | *dltB* | Protein DltB |  |  |  |  | 2.2 |  |  |  |  |  |  |  |
| SAUSA300_0837 | *dltC* | D-alanyl carrier protein | 2.1 |  |  |  |  |  |  |  |  |  |  |  |
| SAUSA300_0838 | *dltD* | Protein DltD precursor | 2.1 |  |  |  |  |  |  |  |  |  |  |  |
| SAUSA300_0841 | *-* | NADH dehydrogenase family |  |  |  |  |  | 2.0 |  | 2.1 | 2.5 | 2.3 |  |  |
| SAUSA300_0842 | *-* | Hypothetical protein |  |  | -2.1 |  |  |  |  |  |  |  |  |  |
| SAUSA300_0846 | *-* | transport protein | 2.6 |  |  |  | 4.2 |  |  |  |  |  |  |  |
| SAUSA300_0847 | *-* | ComA operon protein 2 |  |  |  |  | 2.6 |  |  |  |  |  |  |  |
| SAUSA300_0848 | *-* | Thioredoxin reductase/alkyl hydroperoxide reductase |  |  |  |  |  |  |  |  |  | 2.1 | 2.4 | 6.4 |
| SAUSA300_0857 | *-* | Peptidyl-prolyl cis-trans isomerase (EC 5.2.1.8) |  | 2.7 | 2.5 | 3.0 |  | 2.3 |  |  | 2.6 | 2.1 |  |  |
| SAUSA300_0858 | *-* | S1-type RNA-binding domain |  |  |  |  |  |  |  |  | -2.7 | -2.6 | -2.3 | -2.3 |
| SAUSA300_0859 | *-* | Probable NADH-dependent flavin oxidoreductase yqiG (EC 1.-.-.-) | -2.4 |  |  |  | -2.9 |  |  |  |  |  |  |  |
| SAUSA300_0860 | *rocD* | Ornithine aminotransferase (EC 2.6.1.13) | -2.8 |  |  |  | -2.1 |  |  |  |  | 2.2 | 2.1 |  |
| SAUSA300_0861 | *gudB* | NAD-specific glutamate dehydrogenase (EC 1.4.1.2) |  |  |  |  |  |  |  |  |  |  |  |  |
| SAUSA300_0862 | *glpQ* | Glycerophosphoryl diester phosphodiesterase (EC 3.1.4.46) |  |  | 3.3 | 2.9 | 2.1 | 3.3 | 3.7 | 3.6 | 3.0 | 3.1 | 3.3 | 4.4 |
| SAUSA300_0863 | *argH* | Argininosuccinate lyase (EC 4.3.2.1) |  |  | 3.2 |  |  |  |  |  | 2.6 | 3.5 | 4.4 | 2.4 |
| SAUSA300_0864 | *argG* | Argininosuccinate synthase (EC 6.3.4.5) |  |  | 4.0 |  | 2.4 |  |  |  | 3.1 | 4.1 | 4.0 | 2.9 |
| SAUSA300_0865 | *pgi* | Glucose-6-phosphate isomerase (EC 5.3.1.9) |  |  |  | 2.4 |  |  |  |  |  |  |  |  |
| SAUSA300_0866 | *-* | Alkaline phosphatase like protein |  |  | -2.3 |  |  |  |  |  |  |  |  |  |
| SAUSA300_0872 | *-* | Hypothetical protein |  |  |  | -2.3 |  | -2.5 | -2.2 | -2.4 |  | -2.3 |  |  |
| SAUSA300_0873 | *cdr* | CoA-disulfide reductase (EC 1.8.1.14) | -2.2 |  |  |  |  |  |  |  |  |  |  |  |
| SAUSA300_0874 | *-* | Hydrolase (HAD superfamily) |  |  |  |  |  |  |  | -2.1 |  |  |  |  |
| SAUSA300_0877 | *clpB* | ClpB protein | -2.3 |  |  | -2.4 | -2.1 | -3.1 | -2.7 | -2.6 |  |  |  |  |
| SAUSA300_0878 | *-* | Citrate synthase I repressor |  |  |  | 2.4 |  |  |  | 2.0 |  |  |  |  |
| SAUSA300_0883 | *-* | Outer membrane protein |  |  | 2.6 | 3.7 |  |  |  |  |  |  |  |  |
| SAUSA300_0885 | *fabH* | 3-oxoacyl-[acyl-carrier-protein] synthase III (EC 2.3.1.41) |  |  |  |  | 2.2 |  |  | -2.1 |  |  |  |  |
| SAUSA300_0886 | *fabF* | 3-oxoacyl-[acyl-carrier-protein] synthase (EC 2.3.1.41) |  |  | 2.2 |  |  |  |  |  |  |  |  |  |
| SAUSA300_0887 | *oppB* | Oligopeptide transport system permease protein oppB | -4.7 | -2.4 | -4.7 | -2.1 |  | 2.3 |  | 2.5 |  | 2.3 |  |  |
| SAUSA300_0888 | *oppC* | Oligopeptide transport system permease protein oppC | -4.7 | -2.2 | -4.3 | -2.3 |  | 2.5 |  | 2.7 |  | 2.2 |  |  |
| SAUSA300_0889 | *oppD* | Oligopeptide transport ATP-binding protein oppD | -5.6 | -2.5 | -3.9 | -2.8 |  | 2.6 |  | 2.6 | 2.3 | 3.1 | 2.9 |  |
| SAUSA300_0890 | *oppF* | Oligopeptide transport ATP-binding protein oppF | -6.1 | -2.6 | -5.1 | -3.3 |  | 2.3 |  | 2.2 |  | 2.4 | 2.7 |  |
| SAUSA300_0891 | *oppA* | Oligopeptide-binding protein oppA | -6.5 | -2.8 | -5.9 | -3.4 | -2.2 |  |  | 2.0 |  |  | 2.1 |  |
| SAUSA300_0892 | *oppA* | Oligopeptide-binding protein oppA |  |  |  |  |  | 2.3 | 2.5 | 5.3 |  |  |  | 2.2 |
| SAUSA300_0897 | *trpS* | Tryptophanyl-tRNA synthetase (EC 6.1.1.2) | 2.6 | 3.2 | 2.1 | 4.0 | 2.3 | 2.8 | 2.6 | 2.5 | 2.4 | 2.1 |  |  |
| SAUSA300_0901 | *-* | Putative competence protein/transcription factor |  |  | -2.1 |  |  |  |  |  |  |  |  |  |
| SAUSA300_0902 | *pepF* | Oligoendopeptidase F (EC 3.4.24.-) |  |  |  | 2.7 |  |  |  |  |  |  |  |  |
| SAUSA300_0905 | *-* | Organic phosphate binding CYTH family protein |  | 2.1 | 2.5 | 2.6 |  | 2.0 |  |  | 2.0 |  |  |  |
| SAUSA300_0906 | *-* | Hypothetical protein |  |  | 2.1 | 2.4 | 2.1 | 2.1 |  |  | 2.4 | 2.1 |  |  |
| SAUSA300_0907 | *-* | GTP pyrophosphokinase homolog |  |  | 2.0 | 2.0 |  |  |  |  | 2.1 | 2.0 |  |  |
| SAUSA300_0908 | *ppnK* | ATP-NAD kinase (EC 2.7.1.23) |  |  | 2.1 |  |  |  |  |  |  |  |  |  |
| SAUSA300_0914 | *-* | Sodium/proton-dependent alanine carrier protein |  |  |  |  | 2.2 | 11.7 | 15.1 | 36.1 | 5.1 | 8.6 | 10.5 | 8.1 |
| SAUSA300_0915 | *-* | Acetyl esterase (EC 3.1.1.-) |  |  |  |  |  | 2.6 | 2.8 | 4.1 |  | 2.2 | 2.1 |  |
| SAUSA300_0916 | *-* | 2'-5' RNA ligase (EC 6.5.1.-) |  |  | 2.7 |  |  |  |  |  |  |  |  |  |
| SAUSA300_0917 | *-* | Permease |  |  |  |  |  |  |  | -2.3 |  |  |  |  |
| SAUSA300_0922 | *-* | Tellurite resistance protein TerC |  |  | -2.5 | -2.8 |  | -2.3 | -2.8 | -3.9 |  |  | -2.1 | -2.8 |
| SAUSA300_0923 | *htrA* | Endopeptidase degP (EC 3.4.21.-) | 3.3 |  |  | 2.2 | 3.3 | 2.7 | 2.2 |  | 2.4 |  |  |  |
| SAUSA300_0928 | *comK* | Competence transcription factor | -8.7 | -15.8 | -16.8 | -4.8 | -5.4 | -12.2 | -10.0 | -4.2 | -9.4 | -12.0 | -9.1 | -7.5 |
| SAUSA300_0929 | *-* | Hypothetical protein |  |  | 2.4 |  |  | 5.0 | 6.0 | 6.7 | 5.5 | 8.4 | 9.6 | 9.2 |
| SAUSA300_0930 | *-* | Lipoate-protein ligase A (EC 6.3.2.-) |  |  |  |  |  |  |  |  |  | 2.0 |  |  |
| SAUSA300_0931 | *-* | Hypothetical protein |  |  |  |  |  |  |  | -2.1 | -3.5 | -3.3 | -3.2 | -3.8 |
| SAUSA300_0932 | *-* | Hypothetical protein |  |  |  |  |  |  |  | -2.2 |  |  | -2.0 | -2.0 |
| SAUSA300_0934 | *-* | Hypothetical protein |  |  |  |  |  | 6.6 | 10.7 | 33.0 | 2.4 | 6.7 | 8.7 | 6.8 |
| SAUSA300_0935 | *-* | Hypothetical protein |  |  |  |  |  | 5.1 | 8.8 | 26.1 | 2.1 | 5.5 | 7.5 | 6.3 |
| SAUSA300_0936 | *-* | ABC transporter ATP-binding protein |  |  |  |  |  | 3.5 | 5.9 | 15.7 |  | 3.6 | 5.1 | 4.6 |
| SAUSA300_0937 | *-* | Hypothetical protein | 2.1 |  |  |  | 2.0 |  |  |  |  |  | -2.2 | -2.3 |
| SAUSA300_0939 | *-* | Poly(Glycerol-phosphate) alpha-glucosyltransferase (EC 2.4.1.52) | 2.7 | 2.7 | 2.1 | 2.1 |  |  |  |  |  |  | -2.3 | -2.4 |
| SAUSA300_0940 | *-* | Hypothetical membrane spanning protein | 2.3 |  |  |  |  |  |  |  |  |  |  | -2.1 |
| SAUSA300_0941 | *-* | Iron(III) dicitrate-binding protein |  | -2.4 | -2.6 | -2.8 | -2.1 | -2.5 | -3.2 | -2.3 | -2.1 | -2.6 | -2.9 | -2.5 |
| SAUSA300_0943 | *-* | Hypothetical protein |  |  |  |  |  |  |  |  |  | 2.1 |  |  |
| SAUSA300_0944 | *menA* | 1,4-dihydroxy-2-naphthoate polyprenyltransferase (EC 2.5.1.-) | 2.5 |  |  |  | 2.5 |  |  |  | 2.2 |  |  |  |
| SAUSA300_0945 | *-* | Isochorismate synthase (EC 5.4.4.2) |  |  |  | -2.4 |  |  | -2.6 | -3.1 |  |  | -2.3 | -2.2 |
| SAUSA300_0946 | *menD* | 2-succinyl-6-hydroxy-2,4-cyclohexadiene-1-carboxylate synthase (EC 2.5.1.64) / 2-oxoglutarate decarboxylase (EC 4.1.1.71) |  |  |  | -2.4 |  | -2.2 | -2.6 | -3.2 |  | -2.1 | -2.3 | -2.3 |
| SAUSA300_0947 | *-* | Menaquinone biosynthesis related protein |  |  |  |  |  |  | -2.2 | -3.0 | -2.1 | -2.0 | -2.4 | -2.5 |
| SAUSA300_0949 | *sspC* | Hypothetical protein | -5.4 | -6.8 | -5.6 | -3.7 | -3.4 | -6.2 | -6.9 | -6.8 | -2.4 | -5.9 | -7.9 | -7.5 |
| SAUSA300_0950 | *sspB* | Staphopain (EC 3.4.22.-) | -6.0 | -7.1 | -6.2 | -4.0 | -3.1 | -5.7 | -6.9 | -8.1 | -2.5 | -5.7 | -9.2 | -11.6 |
| SAUSA300_0951 | *sspA* | Glutamyl endopeptidase precursor (EC 3.4.21.19) | -6.3 | -8.5 | -6.6 | -4.6 | -2.9 | -5.6 | -7.5 | -9.2 | -2.5 | -6.0 | -9.1 | -6.1 |
| SAUSA300_0952 | *-* | Aromatic amino acid aminotransferase (EC 2.6.1.57) / Acetyldiaminopimelate aminotransferase (EC 2.6.1.-) |  |  | -2.9 |  |  |  |  | 2.6 |  |  |  |  |
| SAUSA300_0953 | *-* | Permease |  |  |  | -2.1 |  | -2.8 | -4.5 | -6.0 |  |  |  | -3.1 |
| SAUSA300_0954 | *-* | Transcriptional regulator, MarR family |  |  |  |  | 2.9 | 2.1 | 2.3 | 3.6 |  |  |  |  |
| SAUSA300_0955 | *atl* | Peptidoglycan endo-beta-N-acetylglucosaminidase (EC 3.2.1.-) / N-acetylmuramoyl-L-alanine amidase (EC 3.5.1.28) |  |  |  | 2.8 |  | 3.7 | 4.4 | 8.8 |  | 3.0 | 3.2 | 3.4 |
| SAUSA300_0956 | *-* | Acetyltransferase, GNAT family |  |  | -2.2 |  |  |  |  |  |  |  |  |  |
| SAUSA300_0958 | *-* | Transcriptional regulator, LytR family |  |  | -2.5 |  |  |  |  |  |  |  |  |  |
| SAUSA300_0959 | *fmt* | Beta-lactamase family protein |  |  |  |  | 2.2 |  |  |  |  |  |  |  |
| SAUSA300_0965 | *folD* | Methylenetetrahydrofolate dehydrogenase (NADP+) (EC 1.5.1.5) / Methenyltetrahydrofolate cyclohydrolase (EC 3.5.4.9) |  |  |  |  |  | 2.3 |  |  | 2.2 | 2.3 | 2.0 |  |
| SAUSA300_0966 | *purE* | Phosphoribosylaminoimidazole carboxylase carboxyltransferase subunit (EC 4.1.1.21) | 2.5 | 5.4 | 3.1 | 2.1 |  | 30.5 | 11.2 | 7.8 | 41.6 | 30.0 | 9.0 | 5.4 |
| SAUSA300_0967 | *purK* | Phosphoribosylaminoimidazole carboxylase NCAIR mutase subunit (EC 4.1.1.21) | 2.3 | 6.3 | 3.0 |  |  | 26.9 | 9.6 | 6.5 | 36.4 | 26.8 | 8.4 | 4.7 |
| SAUSA300_0968 | *purC* | Phosphoribosylamidoimidazole-succinocarboxamide synthase (EC 6.3.2.6) |  | 5.8 | 2.7 |  | -2.1 | 17.8 | 6.8 | 3.9 | 31.8 | 22.4 | 7.5 | 5.0 |
| SAUSA300_0969 | *purS* | Phosphoribosylformylglycinamidine synthase, purS component (EC 6.3.5.3) |  | 5.5 | 2.6 |  | -3.3 | 14.3 | 6.0 | 3.0 | 27.2 | 19.3 | 6.5 | 3.6 |
| SAUSA300_0970 | *purQ* | Phosphoribosylformylglycinamidine synthase (EC 6.3.5.3) |  | 5.0 | 2.2 |  | -3.4 | 13.3 | 5.4 | 2.7 | 21.4 | 15.9 | 6.3 | 4.2 |
| SAUSA300_0971 | *purL* | Phosphoribosylformylglycinamidine synthase (EC 6.3.5.3) | -3.7 | 3.7 |  |  | -6.7 | 10.5 | 4.4 |  | 12.1 | 9.5 | 3.8 | 2.3 |
| SAUSA300_0972 | *purF* | Amidophosphoribosyltransferase (EC 2.4.2.14) | -5.1 | 2.4 |  |  | -8.6 | 7.2 | 3.0 |  | 9.4 | 7.9 | 3.7 | 2.4 |
| SAUSA300_0973 | *purM* | Phosphoribosylformylglycinamidine cyclo-ligase (EC 6.3.3.1) | -6.2 |  |  | -2.4 | -11.6 | 5.4 | 2.2 |  | 7.1 | 5.8 | 2.6 |  |
| SAUSA300_0974 | *purN* | Phosphoribosylglycinamide formyltransferase (EC 2.1.2.2) | -6.5 |  |  | -2.4 | -11.7 | 3.8 |  |  | 4.8 | 4.1 | 2.2 |  |
| SAUSA300_0975 | *purH* | Phosphoribosylaminoimidazolecarboxamide formyltransferase (EC 2.1.2.3) / IMP cyclohydrolase (EC 3.5.4.10) | -6.7 |  |  | -2.1 | -10.4 | 4.4 |  |  | 6.4 | 5.3 | 2.5 |  |
| SAUSA300_0976 | *purD* | Phosphoribosylamine--glycine ligase (EC 6.3.4.13) | -7.0 |  |  | -2.1 | -12.2 | 3.3 |  |  | 4.2 | 3.6 | 2.0 |  |
| SAUSA300_0977 | *-* | Hydroxymethylpyrimidine transport system permease protein |  |  |  |  | 2.6 | 2.4 | 4.7 | 8.1 |  | 2.6 | 4.9 | 6.2 |
| SAUSA300_0978 | *-* | Hydroxymethylpyrimidine transport ATP-binding protein |  |  |  |  | 2.9 | 2.9 | 5.2 | 9.3 |  | 2.8 | 4.6 | 6.2 |
| SAUSA300_0979 | *-* | Hydroxymethylpyrimidine transport system permease protein |  |  |  |  | 2.5 | 2.7 | 4.4 | 7.5 |  |  | 3.1 | 3.9 |
| SAUSA300_0982 | *-* | Radical activating enzymes proteins | -6.1 | -3.8 | -3.9 | -4.7 | -10.3 | -8.5 | -7.0 | -6.2 | -8.5 | -7.3 | -2.7 |  |
| SAUSA300_0985 | *-* | Hypothetical protein | 3.3 |  |  |  | 3.3 |  |  |  |  |  |  |  |
| SAUSA300_0986 | *-* | Cytochrome d ubiquinol oxidase subunit I (EC 1.10.3.-) | 7.0 |  | 3.3 | 2.2 | 4.0 | 2.1 | 2.7 | 2.7 |  |  | 3.5 | 4.4 |
| SAUSA300_0987 | *-* | Cytochrome d ubiquinol oxidase subunit II (EC 1.10.3.-) | 6.1 | 2.1 | 4.0 |  | 3.0 |  | 2.1 |  |  |  | 2.1 | 2.6 |
| SAUSA300_0990 | *-* | Putative transcriptional regulator |  |  |  |  |  |  |  |  |  |  |  | -2.1 |
| SAUSA300_0995 | *-* | Dihydrolipoamide acetyltransferase component of pyruvate dehydrogenase complex (EC 2.3.1.12) |  |  |  |  |  |  |  | -2.2 |  |  |  |  |
| SAUSA300_0997 | *-* | Hypothetical cytosolic protein |  |  |  |  |  |  |  |  |  |  | -2.1 |  |
| SAUSA300_0998 | *-* | spermidine/putrescine transport operon transcriptional regulator | 4.9 |  |  |  | 6.4 | 4.2 | 3.1 | 2.2 | 3.1 |  |  |  |
| SAUSA300_0999 | *potA* | Spermidine/putrescine transport ATP-binding protein potA | 4.8 |  |  |  | 7.0 | 4.2 | 3.3 | 2.4 | 3.1 |  |  |  |
| SAUSA300_1000 | *potB* | Spermidine/putrescine transport system permease protein PotB | 4.7 |  |  |  | 6.5 | 3.6 | 3.2 | 2.4 | 3.3 | 2.2 |  |  |
| SAUSA300_1001 | *potC* | Spermidine/putrescine transport system permease protein PotC | 5.4 |  |  |  | 7.0 | 3.8 | 3.5 | 2.3 | 4.3 | 2.7 |  |  |
| SAUSA300_1002 | *potD* | Spermidine/putrescine-binding protein |  |  |  | -3.1 |  |  |  |  |  |  | -2.6 | -3.7 |
| SAUSA300_1003 | *-* | Hypothetical protein |  |  | -2.1 |  |  |  |  |  | -2.6 | -2.5 | -2.4 | -2.5 |
| SAUSA300_1004 | *-* | Hypothetical protein |  |  |  | 2.2 |  |  |  |  |  |  |  |  |
| SAUSA300_1005 | *-* | Manganese transport protein MntH |  |  | -2.1 |  | -2.0 | -3.0 | -2.8 | -2.1 | -3.5 | -3.3 | -3.7 | -4.1 |
| SAUSA300_1008 | *-* | Hypothetical protein | 2.1 |  |  |  |  |  |  |  |  |  |  |  |
| SAUSA300_1009 | *typA* | GTP-binding protein TypA/BipA |  | -3.2 | -3.2 | -8.6 |  | -3.3 | -4.0 | -6.8 | -2.3 | -3.2 | -3.9 | -3.5 |
| SAUSA300_1012 | *-* | Hypothetical protein |  |  |  |  |  |  |  |  | 2.4 |  |  |  |
| SAUSA300_1013 | *-* | Cell division protein FtsW |  |  | -2.4 |  |  |  |  |  |  |  |  |  |
| SAUSA300_1014 | *pyc* | Pyruvate carboxylase (EC 6.4.1.1) |  |  |  | 2.7 | 4.3 | 6.4 | 5.9 | 6.7 | 4.3 | 4.2 | 3.2 | 2.7 |
| SAUSA300_1015 | *ctaA* | Heme O monooxygenase (EC 1.-.-.-) | 2.7 |  |  |  |  |  |  |  |  |  |  |  |
| SAUSA300_1016 | *cyoE* | Protoheme IX farnesyltransferase (EC 2.5.1.-) | 2.6 |  | 2.0 | 2.3 |  |  |  |  |  |  |  |  |
| SAUSA300_1017 | *-* | Hypothetical membrane spanning protein | 2.0 |  |  | 2.1 |  |  |  |  |  |  |  |  |
| SAUSA300_1021 | *-* | Hypothetical cytosolic protein | 2.1 |  |  |  |  |  |  |  |  |  |  |  |
| SAUSA300_1022 | *-* | Hypothetical protein |  |  |  |  | 2.2 |  |  |  |  |  |  |  |
| SAUSA300_1025 | *-* | Hypothetical cytosolic protein |  |  |  |  |  |  | -2.0 |  |  |  |  |  |
| SAUSA300_1026 | *-* | Hypothetical protein | 2.1 |  |  | -2.1 |  |  |  | -2.3 |  |  |  |  |
| SAUSA300_1027 | *rpmF* | LSU ribosomal protein L32P | 2.6 |  |  |  | 2.1 |  |  |  |  |  |  |  |
| SAUSA300_1028 | *isdB* | Iron transport associated domain-containing protein |  |  |  |  | 27.0 | 200.6 | 240.1 | 233.9 | 29.6 | 95.7 | 106.7 | 138.6 |
| SAUSA300_1029 | *isdA* | Iron transport associated domain-containing protein |  | 2.3 |  |  | 9.2 | 35.9 | 47.8 | 50.4 | 6.5 | 20.7 | 21.5 | 23.6 |
| SAUSA300_1030 | *isdC* | Iron transport associated domain-containing protein |  | 2.6 |  |  | 53.8 | 128.7 | 141.5 | 181.2 | 43.5 | 70.2 | 60.0 | 68.2 |
| SAUSA300_1031 | *isdD* | Hypothetical membrane associated protein |  |  |  |  | 20.1 | 45.7 | 49.4 | 60.9 | 21.2 | 31.0 | 24.9 | 32.8 |
| SAUSA300_1032 | *isdE* | Ferrichrome-binding protein |  |  |  |  | 30.4 | 80.8 | 80.4 | 113.6 | 27.6 | 47.1 | 40.2 | 47.2 |
| SAUSA300_1033 | *isdF* | Transporter |  |  |  |  | 20.6 | 48.2 | 50.7 | 66.7 | 18.1 | 31.2 | 27.4 | 31.9 |
| SAUSA300_1034 | *srtB* | Sortase B family protein |  | 2.2 |  |  | 20.9 | 52.5 | 59.2 | 73.4 | 23.8 | 43.3 | 35.4 | 43.5 |
| SAUSA300_1035 | *isdG* | Heme-degrading monooxygenase IsdG |  | 2.2 |  |  | 17.8 | 40.7 | 44.9 | 53.2 | 14.5 | 25.9 | 20.2 | 24.3 |
| SAUSA300_1037 | *pheS* | Phenylalanyl-tRNA synthetase alpha chain (EC 6.1.1.20) | 2.5 | 2.1 |  |  | 2.7 | 2.0 |  |  |  |  |  |  |
| SAUSA300_1038 | *pheT* | Phenylalanyl-tRNA synthetase beta chain (EC 6.1.1.20) | 2.5 | 2.2 |  |  | 2.6 |  |  |  |  |  |  |  |
| SAUSA300_1042 | *-* | DNA Polymerase X family (EC 2.7.7.7) |  | -2.2 | -2.2 | -3.2 |  |  | -2.8 | -3.9 |  |  | -2.4 | -2.3 |
| SAUSA300_1043 | *mutS2* | DNA mismatch repair protein mutS |  |  |  | -2.2 |  |  |  | -2.7 |  |  |  |  |
| SAUSA300_1045 | *uvrC* | Excinuclease ABC subunit C |  |  |  |  |  | -2.8 | -3.1 | -2.8 | -2.0 |  |  |  |
| SAUSA300_1046 | *sdhC* | Succinate dehydrogenase cytochrome b558 subunit (EC 1.3.99.1) |  |  |  |  | -2.9 | -8.4 | -6.4 | -10.6 | -2.1 | -2.3 |  | -2.4 |
| SAUSA300_1047 | *sdhA* | Succinate dehydrogenase flavoprotein subunit (EC 1.3.99.1) |  |  |  | -2.7 | -3.2 | -8.5 | -7.9 | -9.3 | -2.1 | -2.4 |  | -2.3 |
| SAUSA300_1048 | *sdhB* | Succinate dehydrogenase iron-sulfur protein (EC 1.3.99.1) |  |  |  | -2.7 | -3.4 | -9.2 | -10.2 | -8.9 | -2.5 | -2.9 | -2.1 | -2.4 |
| SAUSA300_1052 | *-* | Fibrinogen-binding protein precursor | 3.4 | 2.3 |  |  | 3.7 | 2.6 | 2.7 |  | 3.7 | 3.8 | 3.3 | 2.5 |
| SAUSA300_1053 | *-* | Hypothetical protein |  | -2.3 |  |  |  |  | 3.5 | 5.0 | 2.7 | 4.6 | 11.0 | 13.7 |
| SAUSA300_1055 | *efb* | Fibrinogen-binding protein precursor |  |  |  |  |  |  |  |  | 2.1 | 3.0 | 3.1 | 2.5 |
| SAUSA300_1056 | *-* | Hypothetical protein |  |  |  |  |  |  |  |  | 2.8 | 4.0 | 4.3 | 3.0 |
| SAUSA300_1058 | *hla* | Alpha-hemolysin |  |  |  | 2.8 | 2.5 | 2.3 | 3.0 | 3.6 | 3.1 | 2.5 | 2.8 | 3.3 |
| SAUSA300_1059 | *-* | Exotoxin |  |  |  |  |  |  |  |  |  | 2.3 | 4.7 | 4.6 |
| SAUSA300_1060 | *-* | Exotoxin |  |  |  |  |  |  |  |  | 2.1 | 3.1 | 5.9 | 5.6 |
| SAUSA300_1061 | *-* | Exotoxin |  |  |  |  |  |  |  | 2.6 | 2.9 | 4.7 | 8.6 | 7.6 |
| SAUSA300_1064 | *-* | Arginine/ornithine antiporter | -2.8 | -3.6 | -2.6 | -3.2 | -4.3 | -4.6 | -4.0 | -3.2 | -3.4 | -3.5 | -2.5 |  |
| SAUSA300_1065 | *-* | Transporter |  | 2.1 | 4.2 | 4.1 |  | 2.4 | 2.6 | 2.5 |  |  |  |  |
| SAUSA300_1067 | *-* | Phenol soluble modulin | -41.7 | -7.3 | -3.1 |  | -128.5 | -326.8 | -232.0 | -90.2 | -66.7 | -90.8 | -21.5 | -14.3 |
| SAUSA300_1068 | *-* | Phenol soluble modulin beta 1 | -36.2 | -7.4 | -3.2 |  | -81.3 | -206.4 | -179.1 | -82.8 | -54.8 | -61.3 | -18.2 | -9.9 |
| SAUSA300_1069 | *-* | Hydrolase (HAD superfamily) |  |  |  |  |  |  |  | -2.0 |  |  |  | -2.1 |
| SAUSA300_1071 | *-* | Hypothetical membrane spanning protein |  | -2.1 | -2.5 | -2.5 | -2.1 | -2.3 | -2.9 | -3.7 |  |  |  | -2.1 |
| SAUSA300_1072 | *mraZ* | Cell division protein MraZ |  |  |  |  |  | -2.4 | -2.4 | -2.7 |  | -2.4 | -2.0 |  |
| SAUSA300_1073 | *mraW* | S-adenosyl-methyltransferase MraW (EC 2.1.1.-) |  |  |  |  |  |  | -2.0 |  |  | -2.3 | -2.1 | -2.4 |
| SAUSA300_1075 | *pbpA* | Division specific D,D-transpeptidase / Cell division protein FtsI |  |  | -2.2 | -2.0 |  | -2.3 | -2.8 | -3.3 |  | -2.8 | -2.8 | -3.0 |
| SAUSA300_1076 | *mraY* | Phospho-N-acetylmuramoyl-pentapeptide-transferase (EC 2.7.8.13) |  |  |  |  |  | -2.0 | -2.8 | -4.0 |  | -2.4 | -2.9 | -2.9 |
| SAUSA300_1077 | *murD* | UDP-N-acetylmuramoylalanine--D-glutamate ligase (EC 6.3.2.9) |  |  |  |  |  | -2.0 | -2.6 | -3.6 |  |  | -2.4 | -2.5 |
| SAUSA300_1078 | *divIB* | Hypothetical protein |  |  |  |  |  |  | -2.1 | -2.8 |  |  | -2.0 | -2.1 |
| SAUSA300_1082 | *-* | Pyridoxal-5'-phosphate family protein |  |  | -2.3 |  |  | -2.0 |  |  | -2.3 |  |  | -2.6 |
| SAUSA300_1083 | *-* | Hypothetical cytosolic protein |  |  |  |  |  | -2.1 |  |  | -2.1 |  |  |  |
| SAUSA300_1085 | *-* | RNA binding protein |  |  |  | -2.4 |  | -2.3 | -2.8 | -3.5 | -2.9 | -2.9 | -3.1 | -3.6 |
| SAUSA300_1086 | *-* | Cell division initiation protein DivIVA |  |  |  |  |  |  |  |  |  |  |  |  |
| SAUSA300_1087 | *ileS* | Isoleucyl-tRNA synthetase (EC 6.1.1.5) | 2.6 | 2.5 | 2.1 |  | 2.9 | 2.3 | 2.5 |  | 2.8 | 2.4 |  |  |
| SAUSA300_1088 | *-* | Glyoxalase family protein |  |  |  |  |  |  |  | -2.3 |  |  |  | -2.2 |
| SAUSA300_1089 | *lspA* | Lipoprotein signal peptidase (EC 3.4.23.36) |  | -2.2 | -2.1 | -2.7 |  |  | -2.2 | -2.7 |  |  | -2.1 | -2.1 |
| SAUSA300_1090 | *-* | Ribosomal large subunit pseudouridine synthase D (EC 4.2.1.70) |  |  |  | -2.4 |  |  |  | -2.1 |  |  |  |  |
| SAUSA300_1091 | *pyrR* | Uracil phosphoribosyltransferase (EC 2.4.2.9) / Pyrimidine operon regulatory protein PyrR |  | -2.9 |  | -2.6 | 2.4 |  |  | -3.1 |  |  |  |  |
| SAUSA300_1092 | *pyrP* | Uracil permease |  | -12.3 | 2.5 | -3.7 | 5.2 |  | -3.6 | -14.6 | 2.2 |  |  |  |
| SAUSA300_1093 | *pyrB* | Aspartate carbamoyltransferase (EC 2.1.3.2) |  | -7.7 | 3.6 | -2.2 | 5.7 |  | -2.6 | -14.3 | 2.7 |  |  |  |
| SAUSA300_1094 | *pyrC* | Dihydroorotase (EC 3.5.2.3) |  | -5.0 | 5.6 |  | 9.0 |  |  | -8.7 | 3.8 |  |  |  |
| SAUSA300_1095 | *carA* | Carbamoyl-phosphate synthase small chain (EC 6.3.5.5) | 2.1 | -2.8 | 8.1 |  | 11.5 | 3.0 |  | -5.0 | 5.7 | 2.5 |  |  |
| SAUSA300_1096 | *carB* | Carbamoyl-phosphate synthase large chain (EC 6.3.5.5) | 4.2 |  | 16.1 | 2.3 | 27.5 | 5.6 |  | -2.0 | 11.1 | 4.3 | 2.3 | 2.0 |
| SAUSA300_1097 | *pyrF* | Orotidine 5'-phosphate decarboxylase (EC 4.1.1.23) | 8.8 |  | 37.0 | 6.8 | 57.9 | 11.4 | 3.6 |  | 24.0 | 9.5 | 4.8 | 3.9 |
| SAUSA300_1098 | *pyrE* | Orotate phosphoribosyltransferase (EC 2.4.2.10) | 8.1 |  | 29.9 | 6.7 | 45.9 | 10.5 | 3.6 |  | 19.2 | 8.5 | 4.8 | 3.8 |
| SAUSA300_1099 | *-* | Hypothetical protein |  |  | 7.1 | 2.2 | 8.6 | 2.5 |  |  | 3.9 |  |  |  |
| SAUSA300_1101 | *-* | Fibronectin-binding protein / Fibrinogen-binding protein |  | -2.1 | -2.4 | -2.2 | -2.1 | -2.4 | -2.8 | -2.9 | -2.4 | -2.1 | -2.2 | -2.6 |
| SAUSA300_1106 | *-* | Hypothetical protein |  |  |  |  |  |  |  |  |  |  | -2.1 | -2.7 |
| SAUSA300_1107 | *-* | Hypothetical protein |  |  |  |  | -2.5 |  |  |  |  |  |  |  |
| SAUSA300_1109 | *fmt* | Methionyl-tRNA formyltransferase (EC 2.1.2.9) |  |  |  |  |  |  |  |  |  | -2.1 | -2.2 | -2.1 |
| SAUSA300_1110 | *sun* | 16S rRNA m(5)C 967 methyltransferase (EC 2.1.1.-) |  |  |  | -2.2 |  |  |  |  |  |  |  |  |
| SAUSA300_1118 | *-* | General stress protein, Gls24 family |  | -2.1 |  |  |  |  |  | -2.2 |  |  |  |  |
| SAUSA300_1119 | *-* | Dihydroxyacetone kinase family protein |  |  |  | -2.2 |  |  |  | -2.4 |  |  | -2.3 | -2.2 |
| SAUSA300_1121 | *-* | PaaI family protein, possible transcriptional regulator |  |  |  |  |  |  |  | -3.3 |  | -2.0 | -2.1 | -2.0 |
| SAUSA300_1122 | *plsX* | Fatty acid/phospholipid synthesis protein PlsX |  |  |  |  |  |  |  | -3.2 |  |  |  |  |
| SAUSA300_1123 | *fabD* | Malonyl-CoA-[acyl-carrier-protein] transacylase (EC 2.3.1.39) |  |  |  |  |  |  |  | -2.5 |  |  |  |  |
| SAUSA300_1124 | *fabG* | 3-oxoacyl-[acyl-carrier protein] reductase (EC 1.1.1.100) |  |  | 2.2 |  |  |  |  |  |  |  |  |  |
| SAUSA300_1127 | *smc* | Chromosome partition protein Smc |  |  |  |  |  |  |  | -2.1 |  |  |  |  |
| SAUSA300_1128 | *ftsY* | Cell division protein FtsY |  |  |  |  |  |  |  | -2.1 |  |  |  |  |
| SAUSA300_1131 | *rpsP* | SSU ribosomal protein S16P | 3.4 |  |  |  | 2.8 |  |  |  |  |  |  |  |
| SAUSA300_1132 | *rimM* | 16S rRNA processing protein RimM | 3.9 |  |  | -3.2 | 3.0 |  |  | -2.2 |  |  |  | -2.1 |
| SAUSA300_1133 | *trmD* | tRNA (Guanine-N(1)-)-methyltransferase (EC 2.1.1.31) | 3.8 |  |  | -2.7 | 3.1 |  |  | -2.0 |  |  |  |  |
| SAUSA300_1134 | *rplS* | LSU ribosomal protein L19P |  |  |  |  |  |  |  | -2.1 |  |  |  |  |
| SAUSA300_1140 | *lytN* | Cell wall hydrolase LytN |  |  |  |  |  | -2.3 | -2.2 | -2.1 |  |  |  |  |
| SAUSA300_1142 | *dprA* | DNA processing protein |  |  |  |  |  |  |  | 2.6 |  |  |  |  |
| SAUSA300_1143 | *topA* | DNA topoisomerase I (EC 5.99.1.2) |  |  | -2.3 |  |  |  |  |  |  |  |  |  |
| SAUSA300_1149 | *rpsB* | SSU ribosomal protein S2P |  |  |  | -2.3 |  |  |  | -2.1 |  |  |  |  |
| SAUSA300_1152 | *frr* | Ribosome Recycling Factor (RRF) | 2.0 |  | 2.1 |  | 2.3 |  |  |  |  |  |  |  |
| SAUSA300_1154 | *cdsA* | Phosphatidate cytidylyltransferase (EC 2.7.7.41) | 2.2 |  |  |  |  |  |  |  |  |  |  |  |
| SAUSA300_1158 | *-* | Hypothetical cytosolic protein |  | -3.0 | -3.4 | -3.3 |  | -2.2 | -2.6 | -3.4 |  | -2.4 | -2.3 |  |
| SAUSA300_1159 | *nusA* | N utilization substance protein A |  | -2.2 | -2.7 | -3.1 |  |  | -2.3 | -2.9 |  |  | -2.0 |  |
| SAUSA300_1160 | *-* | Hypothetical cytosolic protein |  |  |  | -2.1 |  |  |  | -2.2 |  |  |  |  |
| SAUSA300_1161 | *-* | LSU ribosomal protein L7AE |  |  | -2.2 | -2.7 |  |  | -2.0 | -2.9 |  |  | -2.3 | -2.2 |
| SAUSA300_1163 | *rbfA* | Ribosome-binding factor A | 2.2 |  |  |  |  |  |  |  |  |  |  |  |
| SAUSA300_1164 | *truB* | tRNA pseudouridine synthase B (EC 4.2.1.70) |  |  |  |  |  |  |  | -2.5 |  |  |  |  |
| SAUSA300_1166 | *rpsO* | SSU ribosomal protein S15P |  |  |  |  |  |  |  | -2.3 |  |  |  |  |
| SAUSA300_1167 | *pnpA* | Polyribonucleotide nucleotidyltransferase (EC 2.7.7.8) / Polynucleotide adenylyltransferase (EC 2.7.7.19) |  |  | -2.2 |  |  | -2.1 | -2.4 | -2.4 |  | -2.1 | -2.6 | -3.0 |
| SAUSA300_1169 | *ftsK* | Cell division protein FtsK |  |  | -2.3 | -2.1 |  | -2.3 | -2.0 |  | -2.4 | -2.4 | -2.4 | -2.5 |
| SAUSA300_1173 | *-* | Acetoacetyl-CoA reductase (EC 1.1.1.36) |  |  |  |  |  |  |  |  |  |  |  | -2.1 |
| SAUSA300_1175 | *-* | Transcriptional regulator |  |  |  |  |  |  |  |  |  |  |  | -2.0 |
| SAUSA300_1178 | *recA* | RecA protein |  |  |  |  |  |  | -2.1 |  |  |  |  |  |
| SAUSA300_1180 | *-* | Hypothetical protein |  |  |  |  | -2.4 |  |  | -2.5 |  |  |  |  |
| SAUSA300_1188 | *mutS* | DNA mismatch repair protein MutS |  | -2.1 | -2.6 | -2.4 |  |  | -2.3 | -2.3 |  | -2.2 | -2.4 | -2.3 |
| SAUSA300_1189 | *mutL* | DNA mismatch repair protein MutL |  |  |  | -2.1 |  |  |  |  |  | -2.0 | -2.2 | -2.1 |
| SAUSA300_1191 | *glpF* | Glycerol uptake facilitator protein | -2.6 | -2.6 | -6.3 | -8.4 | -3.4 | -8.7 | -7.9 | -11.4 | -4.6 | -4.8 | -2.5 |  |
| SAUSA300_1192 | *glpK* | Glycerol kinase (EC 2.7.1.30) |  | -3.2 | -5.5 | -8.1 | -2.8 | -7.1 | -8.0 | -11.1 | -4.9 | -5.0 | -3.8 | -2.7 |
| SAUSA300_1193 | *glpD* | Glycerol-3-phosphate dehydrogenase (EC 1.1.99.5) | -3.4 | -3.0 | -9.8 | -5.4 | -10.9 | -10.5 | -14.7 | -22.4 | -10.2 | -13.0 | -7.8 | -4.5 |
| SAUSA300_1194 | *-* | Lysophospholipase L2 (EC 3.1.1.5) |  |  |  |  |  |  |  |  |  |  | -2.4 | -2.6 |
| SAUSA300_1196 | *hfq* | RNA-binding protein, Hfq family |  |  |  |  |  |  |  |  |  |  | -2.1 | -2.3 |
| SAUSA300_1197 | *-* | Glutathione peroxidase (EC 1.11.1.9) |  | 2.4 | 2.8 | 3.4 |  | 2.5 | 2.1 |  | 3.3 | 2.7 | 2.2 |  |
| SAUSA300_1199 | *-* | Aluminum resistance protein | 2.0 |  |  |  |  |  |  |  |  |  |  |  |
| SAUSA300_1200 | *glnR* | Transcriptional regulator, MerR family | 2.7 |  |  |  | -3.0 |  |  |  | -2.3 |  |  |  |
| SAUSA300_1201 | *glnA* | Glutamine synthetase (EC 6.3.1.2) | 2.6 |  | 2.0 |  | -2.3 |  |  |  |  |  |  |  |
| SAUSA300_1204 | *-* | Hypothetical protein |  |  | 2.1 | 2.3 | 3.5 | 3.6 | 3.4 | 3.4 | 2.1 |  |  |  |
| SAUSA300_1205 | *-* | Hypothetical protein |  |  |  | 2.4 | 2.8 | 3.1 | 2.6 | 2.4 |  |  |  |  |
| SAUSA300_1208 | *-* | Hypothetical protein | 3.8 | 3.2 | 3.2 | 4.9 | 4.1 | 5.2 | 4.9 | 4.4 | 3.7 | 3.3 | 2.6 | 2.8 |
| SAUSA300_1214 | *-* | Hypothetical protein |  |  | -2.3 |  |  |  | -2.0 |  |  |  |  |  |
| SAUSA300_1215 | *-* | Hypothetical protein |  |  |  | 2.4 |  |  | 2.3 | 2.1 |  |  |  |  |
| SAUSA300_1216 | *-* | Cardiolipin synthetase (EC 2.7.8.-) |  |  |  | 2.1 |  |  |  |  |  |  |  |  |
| SAUSA300_1217 | *-* | ABC transporter ATP-binding protein |  |  |  |  |  | 2.7 |  |  | 2.8 | 4.1 | 3.1 | 2.1 |
| SAUSA300_1218 | *-* | ABC transporter permease protein |  |  |  |  |  | 2.5 |  |  | 2.2 | 3.5 | 3.0 |  |
| SAUSA300_1221 | *-* | Hypothetical protein |  |  |  |  |  |  |  |  |  | -2.1 |  |  |
| SAUSA300_1222 | *nuc* | Thermonuclease (EC 3.1.31.1) | 4.2 |  |  | -2.1 | 3.8 |  |  |  |  |  |  |  |
| SAUSA300_1223 | *-* | Hypothetical protein |  |  | 2.4 | 3.0 |  |  |  |  |  |  |  |  |
| SAUSA300_1224 | *-* | Hypothetical protein |  |  | 2.3 |  |  |  |  |  |  |  |  |  |
| SAUSA300_1225 | *-* | Aspartokinase (EC 2.7.2.4) | -2.2 | -3.4 | -2.6 |  |  | 4.8 | 9.8 | 22.5 |  | 4.1 | 5.8 | 5.1 |
| SAUSA300_1226 | *-* | Homoserine dehydrogenase (EC 1.1.1.3) | -2.4 | -3.1 | -4.3 |  |  | 4.3 | 7.2 | 13.4 |  | 3.6 | 5.4 | 4.5 |
| SAUSA300_1227 | *thrC* | Threonine synthase (EC 4.2.3.1) | -2.3 | -3.4 | -3.8 | -2.5 |  | 3.7 | 5.9 | 11.9 |  | 3.0 | 5.1 | 4.0 |
| SAUSA300_1228 | *thrB* | Homoserine kinase (EC 2.7.1.39) | -2.5 | -3.3 | -3.8 | -2.4 |  | 3.7 | 6.2 | 13.1 |  | 3.6 | 6.2 | 5.1 |
| SAUSA300_1229 | *-* | Hydrolase (HAD superfamily) |  |  |  |  |  |  | 2.9 | 4.5 |  | 2.1 | 2.7 | 2.3 |
| SAUSA300_1230 | *-* | Hypothetical protein |  | 2.7 | 2.5 | 2.2 |  |  |  |  |  |  |  |  |
| SAUSA300_1231 | *-* | Lysine-specific permease |  | 2.3 |  | 2.4 |  |  | 2.4 | 3.4 |  |  |  |  |
| SAUSA300_1232 | *-* | Catalase (EC 1.11.1.6) | -2.2 |  | -2.9 | -2.6 | -2.6 | -2.7 | -2.4 |  |  |  |  |  |
| SAUSA300_1233 | *rpmG* | Lsu ribosomal protein L33P | 2.1 |  |  |  | 2.0 |  |  |  |  |  |  |  |
| SAUSA300_1234 | *rpsN* | Ssu ribosomal protein S14P | 2.8 |  | 2.4 | 3.0 | 3.2 |  | 2.2 | 2.2 |  |  |  |  |
| SAUSA300_1235 | *guaC* | GMP reductase (EC 1.7.1.7) | 8.2 | 3.8 | 2.2 |  | 7.1 | 8.1 | 3.1 |  | 20.6 | 11.4 | 3.6 | 3.2 |
| SAUSA300_1236 | *-* | Hypothetical exported protein | 6.8 | 4.5 | 3.2 | 2.4 | 5.1 | 6.5 | 3.6 | 2.1 | 12.5 | 8.2 | 3.1 | 2.0 |
| SAUSA300_1237 | *lexA* | LexA repressor (EC 3.4.21.88) |  |  | -2.2 | -2.3 | -2.8 | -3.2 | -2.7 | -2.2 |  |  |  |  |
| SAUSA300_1239 | *tkt* | Transketolase (EC 2.2.1.1) |  |  |  |  |  |  |  |  | 2.1 |  |  |  |
| SAUSA300_1240 | *-* | Hypothetical exported protein | 2.3 |  |  |  |  |  |  |  |  |  |  |  |
| SAUSA300_1241 | *-* | CcdC protein |  | -2.1 |  |  |  |  |  | -2.7 |  |  | -2.2 | -2.1 |
| SAUSA300_1243 | *sbcC* | Exonuclease SbcC (EC 3.1.11.-) |  |  |  |  |  |  |  |  |  |  |  | -2.1 |
| SAUSA300_1246 | *acnA* | Aconitate hydratase (EC 4.2.1.3) |  |  |  |  | -3.3 | -2.1 | -2.6 |  |  |  |  |  |
| SAUSA300_1248 | *-* | Hypothetical cytosolic protein |  |  |  | 2.0 |  |  |  |  |  |  |  |  |
| SAUSA300_1250 | *parE* | Topoisomerase IV subunit B (EC 5.99.1.-) |  |  | -2.0 |  |  |  |  |  |  |  |  | -2.1 |
| SAUSA300_1253 | *glcT* | Transcription antiterminator, BglG family |  | 2.2 |  | 2.6 |  |  |  | 2.1 |  | 2.1 |  |  |
| SAUSA300_1255 | *fmtC* | Lysyltransferase (EC 2.3.2.3) |  |  |  | 2.3 |  |  |  |  |  | 2.6 | 2.2 |  |
| SAUSA300_1256 | *msrA* | Peptide methionine sulfoxide reductase MsrA (EC 1.8.4.11) |  | 2.0 |  | 3.4 |  | 3.0 | 3.0 | 3.6 | 2.2 | 2.6 | 2.4 |  |
| SAUSA300_1259 | *-* | ImpB/MucB/SamB family protein | 2.6 |  |  |  |  |  |  |  |  |  | 2.1 |  |
| SAUSA300_1260 | *-* | Arogenate dehydrogenase (EC 1.3.1.43) / Prephenate dehydrogenase (EC 1.3.1.12) |  |  |  |  |  | 2.2 | 2.2 | 3.6 |  | 2.3 | 2.5 |  |
| SAUSA300_1262 | *trpE* | Anthranilate synthase component I (EC 4.1.3.27) |  |  |  |  |  |  |  |  | 2.3 | 2.3 |  |  |
| SAUSA300_1264 | *trpD* | Anthranilate phosphoribosyltransferase (EC 2.4.2.18) |  |  |  |  |  |  |  | 2.3 | 2.7 | 2.8 | 2.3 | 2.2 |
| SAUSA300_1265 | *trpC* | Indole-3-glycerol phosphate synthase (EC 4.1.1.48) |  |  |  |  |  | 2.1 | 2.2 | 3.1 | 2.7 | 3.2 | 2.7 | 2.4 |
| SAUSA300_1266 | *trpF* | N-(5'-phosphoribosyl)anthranilate isomerase (EC 5.3.1.24) |  |  |  |  |  |  |  | 2.9 | 2.7 | 3.2 | 2.9 | 2.5 |
| SAUSA300_1267 | *trpB* | Tryptophan synthase beta chain (EC 4.2.1.20) |  |  |  |  |  | 2.0 | 2.2 | 3.2 | 2.4 | 3.3 | 3.3 | 2.9 |
| SAUSA300_1269 | *femA* | Hypothetical protein |  | -2.0 |  |  |  | -2.0 | -2.5 | -3.0 |  | -2.0 |  |  |
| SAUSA300_1270 | *femB* | UDP-N-acetylmuramoylpentapeptide-triglycine glycyltransferase (EC 2.3.2.-) |  |  |  |  |  |  | -2.2 | -2.8 |  |  | -2.1 |  |
| SAUSA300_1274 | *-* | Nickel transport ATP-binding protein NikE |  |  |  |  |  | -2.1 |  |  |  |  |  |  |
| SAUSA300_1279 | *phoU* | Phosphate transport system protein PhoU | 3.2 |  |  |  |  |  |  |  |  |  |  |  |
| SAUSA300_1280 | *pstB* | Phosphate transport ATP-binding protein PstB | 3.3 |  |  |  |  |  |  |  |  |  |  |  |
| SAUSA300_1281 | *pstA* | Phosphate transport system permease protein PstA | 3.2 |  |  |  |  |  |  |  |  |  |  | 2.2 |
| SAUSA300_1282 | *pstC* | Phosphate transport system permease protein PstC | 2.3 |  |  |  |  |  |  |  | 2.2 |  |  | 2.3 |
| SAUSA300_1283 | *pstS* | Phosphate-binding protein | 3.6 |  |  |  |  |  |  |  | 2.5 |  |  |  |
| SAUSA300_1284 | *-* | S1 RNA binding domain |  |  |  |  |  |  |  |  |  |  | -2.1 |  |
| SAUSA300_1285 | *-* | ABC transporter ATP-binding protein | 3.3 |  |  |  | 2.5 | 2.2 |  |  | 2.5 |  |  |  |
| SAUSA300_1286 | *-* | Aspartokinase (EC 2.7.2.4) | -4.2 | -2.8 | -4.0 | -2.7 |  | 2.4 | 2.2 | 2.1 | 3.7 | 6.6 | 3.6 |  |
| SAUSA300_1287 | *asd* | Aspartate-semialdehyde dehydrogenase (EC 1.2.1.11) | -4.8 | -2.2 | -2.9 |  |  | 4.1 | 4.3 | 6.3 | 3.5 | 6.3 | 5.5 | 3.7 |
| SAUSA300_1288 | *dapA* | Dihydrodipicolinate synthase (EC 4.2.1.52) | -4.9 | -2.3 | -2.8 | -2.2 |  | 3.5 | 3.6 | 6.2 | 3.8 | 7.0 | 6.8 | 4.2 |
| SAUSA300_1289 | *dapB* | Dihydrodipicolinate reductase (EC 1.3.1.26) | -7.0 | -2.9 | -3.3 | -2.7 |  | 2.7 | 3.0 | 4.9 | 2.7 | 5.4 | 5.7 | 3.5 |
| SAUSA300_1290 | *dapD* | Tetrahydrodipicolinate N-acetyltransferase (EC 2.3.1.89) | -6.8 | -3.4 | -3.4 | -2.9 |  |  | 2.4 | 4.0 | 2.1 | 4.2 | 4.5 | 3.1 |
| SAUSA300_1291 | *-* | Putative N-acetyldiaminopimelate deacetylase (EC 3.5.1.47) | -2.9 | -2.5 | -3.0 | -2.6 |  |  |  | 2.6 |  | 2.6 | 2.4 | 2.0 |
| SAUSA300_1292 | *alr2* | Alanine racemase (EC 5.1.1.1) | -3.0 | -2.1 | -2.9 | -2.2 |  | 2.5 | 2.6 | 3.9 |  | 3.0 | 2.2 |  |
| SAUSA300_1293 | *lysA* | Diaminopimelate decarboxylase (EC 4.1.1.20) | -2.0 |  | -2.0 |  |  |  |  | 2.2 |  | 2.0 |  |  |
| SAUSA300_1294 | *-* | Hypothetical protein |  |  | -2.1 |  |  |  |  |  | -2.1 |  |  |  |
| SAUSA300_1296 | *-* | Putative DNA binding protein |  |  | -2.5 |  | -2.2 |  |  | -2.1 |  |  |  |  |
| SAUSA300_1297 | *-* | Acylphosphatase (EC 3.6.1.7) |  |  |  |  | -2.1 |  |  |  |  |  |  |  |
| SAUSA300_1299 | *-* | Tellurite resistance protein |  |  |  |  | -2.0 |  |  |  |  |  |  |  |
| SAUSA300_1300 | *brnQ* | Branched-chain amino acid transport system carrier protein |  |  | -3.3 | -2.1 |  |  | -2.0 |  |  |  |  |  |
| SAUSA300_1301 | *-* | von Willebrand factor type A domain protein |  |  | -3.0 |  |  | -2.1 | -2.3 |  | -2.9 | -2.3 | -2.7 | -2.8 |
| SAUSA300_1302 | *-* | Hypothetical ATPase |  |  | -5.4 | -2.1 |  | -2.3 | -2.6 | -2.1 | -2.3 |  | -2.1 | -2.3 |
| SAUSA300_1307 | *arlS* | Two-component sensor kinase arlS (EC 2.7.3.-) |  |  |  |  |  |  |  |  |  | -2.3 | -2.6 | -2.6 |
| SAUSA300_1309 | *-* | Transposase | 2.1 |  |  |  | 3.0 | 2.2 | 2.4 |  |  |  |  |  |
| SAUSA300_1311 | *murG* | UDP-N-acetylglucosamine--N-acetylmuramyl-(pentapeptide) pyrophosphoryl-undecaprenol N-acetylglucosamine transferase (EC 2.4.1.227) |  |  |  |  |  |  |  |  |  |  |  | -2.1 |
| SAUSA300_1312 | *-* | Acetyltransferase (EC 2.3.1.-) |  |  |  |  |  |  |  |  | -2.0 | -2.1 | -2.1 | -2.1 |
| SAUSA300_1314 | *-* | Hypothetical cytosolic protein |  |  |  |  |  |  |  |  |  | 2.6 | 2.9 | 2.6 |
| SAUSA300_1315 | *crr* | PTS system, glucose-specific IIA component (EC 2.7.1.69) |  |  |  |  |  |  |  |  |  | 2.7 | 3.0 | 2.7 |
| SAUSA300_1316 | *msrB* | Peptide methionine sulfoxide reductase msrB (EC 1.8.4.11) |  |  |  |  |  |  |  |  |  | 2.6 | 2.8 | 2.4 |
| SAUSA300_1317 | *msrA* | Hypothetical protein |  |  |  |  |  |  |  |  |  | 2.1 | 2.1 |  |
| SAUSA300_1320 | *thyA* | Thymidylate synthase (EC 2.1.1.45) |  |  |  |  |  | -2.0 | -2.2 | -2.3 | -2.1 | -2.5 | -2.6 | -2.9 |
| SAUSA300_1324 | *-* | Hypothetical membrane spanning protein | 3.9 |  |  |  | 2.5 |  |  |  |  |  |  |  |
| SAUSA300_1326 | *-* | Ribonuclease HI (EC 3.1.26.4) / Cell wall enzyme EBSB | 2.8 |  |  |  | 2.1 |  |  |  |  |  |  |  |
| SAUSA300_1328 | *-* | Multidrug resistance protein B | -3.5 |  | 7.2 | 16.6 |  | 6.6 | 10.4 | 12.3 | -3.0 |  | 4.2 | 5.9 |
| SAUSA300_1329 | *-* | Amino acid permease | -4.0 | 3.5 | 15.4 | 27.0 |  | 14.6 | 21.2 | 20.8 | -2.1 |  | 8.3 | 11.9 |
| SAUSA300_1330 | *ilvA* | Threonine dehydratase (EC 4.3.1.19) | -5.3 | 5.4 | 18.2 | 28.7 |  | 19.9 | 27.2 | 23.7 |  |  | 10.7 | 15.6 |
| SAUSA300_1331 | *ald* | Alanine dehydrogenase (EC 1.4.1.1) | -4.1 | 7.6 | 22.3 | 34.7 |  | 31.6 | 43.1 | 27.9 |  |  | 11.2 | 13.9 |
| SAUSA300_1334 | *-* | Sulfite reductase [NADPH] flavoprotein alpha-component (EC 1.8.1.2) | 19.8 | 2.2 | 11.2 | 3.0 | 9.8 |  |  |  |  |  |  |  |
| SAUSA300_1335 | *-* | Hypothetical protein |  | -2.0 |  | -2.9 |  | -2.2 | -2.5 | -3.5 |  |  | -2.2 |  |
| SAUSA300_1336 | *-* | Methyltransferase (EC 2.1.1.-) |  | -3.6 | -3.0 | -4.6 |  | -3.1 | -3.7 | -5.6 | -2.4 | -3.5 | -3.7 | -3.7 |
| SAUSA300_1342 | *-* | Hypothetical protein |  |  |  | -2.4 |  | -2.3 | -2.7 | -3.0 |  |  |  |  |
| SAUSA300_1343 | *nth* | Endonuclease III (EC 4.2.99.18) |  | -2.1 | -2.3 | -2.5 |  | -2.5 | -2.9 | -3.0 |  |  |  |  |
| SAUSA300_1344 | *-* | DNA replication protein DnaD |  | -2.5 | -2.8 | -2.2 |  | -2.3 | -2.9 | -2.7 |  |  |  |  |
| SAUSA300_1345 | *asnC* | Asparaginyl-tRNA synthetase (EC 6.1.1.22) | 2.2 | 2.6 | 2.4 | 2.7 | 2.0 | 2.6 | 2.2 |  | 2.0 |  |  |  |
| SAUSA300_1352 | *-* | Hypothetical membrane spanning protein |  |  | -2.7 |  |  |  |  |  |  |  |  | -2.1 |
| SAUSA300_1356 | *aroB* | 3-dehydroquinate synthase (EC 4.2.3.4) |  |  |  |  |  |  |  | 2.1 |  |  |  |  |
| SAUSA300_1357 | *aroC* | Chorismate synthase (EC 4.2.3.5) |  |  |  |  |  | 2.1 | 2.0 | 2.7 |  |  |  |  |
| SAUSA300_1358 | *ndk* | Nucleoside diphosphate kinase (EC 2.7.4.6) |  |  |  |  |  |  |  | -2.2 |  |  |  |  |
| SAUSA300_1366 | *-* | Hypothetical protein | 2.2 |  |  | 2.3 |  |  |  |  | 2.2 |  |  |  |
| SAUSA300_1367 | *cmk* | Cytidylate kinase (EC 2.7.4.14) | 2.5 | 2.4 |  | 2.7 | 2.3 | 2.0 |  |  |  |  |  |  |
| SAUSA300_1368 | *ansA* | L-asparaginase (EC 3.5.1.1) | 2.9 | 2.4 | 2.2 | 2.3 | 3.0 | 2.7 |  |  | 3.0 | 2.5 |  |  |
| SAUSA300_1369 | *-* | Thioredoxin reductase (EC 1.8.1.9) |  |  |  |  |  |  |  | -2.8 | -2.0 |  | -2.3 | -2.1 |
| SAUSA300_1373 | *-* | Ferredoxin | 2.6 |  |  | -2.1 |  |  |  |  |  |  |  |  |
| SAUSA300_1374 | *-* | Riboflavin transporter |  | -3.4 | -2.4 | -3.0 |  |  | -2.4 | -3.2 |  |  |  |  |
| SAUSA300_1377 | *-* | Hypothetical protein |  |  |  |  |  | -2.0 |  |  |  |  |  |  |
| SAUSA300_1380 | *-* | Hypothetical protein |  | -3.2 | -3.0 | -2.3 |  | -3.4 | -3.4 | -4.1 | -2.1 | -2.1 |  |  |
| SAUSA300_1381 | *lukF-PV* | Leukocidin F subunit | -4.6 | -5.7 | -2.7 |  |  | -3.1 | -2.6 |  |  |  |  | 2.6 |
| SAUSA300_1382 | *lukS-PV* | Leukocidin S subunit | -5.5 | -8.4 | -3.9 |  |  | -3.8 | -3.2 | -2.5 |  |  |  |  |
| SAUSA300_1384 | *-* | Holin |  |  |  |  |  |  |  |  |  |  |  | 2.3 |
| SAUSA300_1388 | *-* | Phage protein |  |  |  |  |  |  |  |  |  |  |  | 2.6 |
| SAUSA300_1393 | *-* | Phage protein |  |  |  |  |  |  |  |  |  |  |  | 2.7 |
| SAUSA300_1404 | *-* | Terminase large subunit |  |  |  |  |  |  |  |  |  |  |  | 2.4 |
| SAUSA300_1409 | *-* | Phage-related protein |  |  |  |  |  |  |  |  |  |  |  | 2.0 |
| SAUSA300_1410 | *-* | Virulence-associated protein E |  |  |  |  |  |  |  |  |  |  |  | 2.0 |
| SAUSA300_1412 | *-* | Transcriptional activator RinB |  |  |  |  |  |  |  |  |  | -2.2 | -2.0 |  |
| SAUSA300_1414 | *-* | Hypothetical protein |  |  |  |  |  |  |  |  |  |  |  | 2.1 |
| SAUSA300_1415 | *-* | Hypothetical protein |  |  |  |  |  |  |  |  |  |  |  | 2.4 |
| SAUSA300_1419 | *-* | Hypothetical protein |  |  |  |  |  |  |  |  |  |  |  | 2.4 |
| SAUSA300_1425 | *-* | Phage protein |  |  |  |  |  |  |  |  |  |  |  | 3.9 |
| SAUSA300_1429 | *-* | Phage protein | 2.7 | -3.0 | -3.1 | -2.8 |  | -2.2 | -3.2 | -2.7 | -3.4 | -4.1 | -2.9 | -3.2 |
| SAUSA300_1434 | *-* | Transcriptional regulator, MerR family |  |  |  |  |  |  |  | 2.0 | 2.1 |  |  |  |
| SAUSA300_1435 | *-* | Zn-dependent alcohol dehydrogenases and related dehydrogenases | 2.5 | 2.4 |  | 2.5 | 3.0 | 2.6 | 2.5 | 3.6 | 3.4 | 2.6 |  |  |
| SAUSA300_1436 | *-* | Hypothetical protein | 2.9 | 3.4 | 3.0 | 3.4 | 4.1 | 3.6 | 3.3 | 4.7 | 5.8 | 4.0 | 2.6 | 2.4 |
| SAUSA300_1440 | *-* | Hypothetical cytosolic protein |  |  |  |  |  |  |  |  | 3.0 |  |  |  |
| SAUSA300_1441 | *srrB* | Sensor protein resE (EC 2.7.3.-) |  |  |  |  |  |  |  |  |  | -2.1 |  |  |
| SAUSA300_1442 | *srrA* | Transcriptional regulatory protein ResD |  | 2.1 | 2.7 | 2.1 |  |  |  |  |  |  |  |  |
| SAUSA300_1443 | *rluB* | Ribosomal large subunit pseudouridine synthase B (EC 4.2.1.70) |  |  |  |  |  |  |  |  |  |  |  | -2.2 |
| SAUSA300_1444 | *scpB* | Segregation and condensation protein ScpB |  |  |  |  |  |  |  |  |  |  | -2.0 | -2.0 |
| SAUSA300_1445 | *scpA* | Segregation and condensation protein ScpA |  | -2.0 |  |  |  |  |  |  |  |  |  |  |
| SAUSA300_1447 | *xerD* | Integrase/recombinase (XerD/RipX family) | -2.4 | -3.1 |  |  |  | -2.8 | -3.0 | -3.1 | -2.5 | -2.0 | -2.1 | -2.6 |
| SAUSA300_1448 | *-* | Ferric uptake regulation protein |  | -2.4 | -2.2 |  |  | -2.2 | -2.2 | -2.5 | -2.2 | -2.1 | -2.3 | -2.8 |
| SAUSA300_1450 | *-* | Oxidoreductase (EC 1.1.1.-) |  |  |  | 2.7 |  |  |  |  | 2.1 | 2.1 |  |  |
| SAUSA300_1451 | *-* | Hypothetical protein |  |  |  | 3.1 |  | 2.0 |  | 2.1 |  |  |  |  |
| SAUSA300_1452 | *proC* | Pyrroline-5-carboxylate reductase (EC 1.5.1.2) |  |  |  | 3.0 |  | 2.5 | 2.2 | 3.3 |  | 2.3 | 2.2 |  |
| SAUSA300_1456 | *-* | Exo-alpha-1,4-glucosidase (EC 3.2.1.20) | -6.1 | -2.9 |  | -7.5 | -4.2 | -7.8 | -5.0 | -5.3 | -2.2 |  |  |  |
| SAUSA300_1457 | *malR* | Maltose operon transcriptional repressor | -6.1 |  |  | -7.1 | -4.0 | -6.5 | -3.6 | -6.0 |  |  |  |  |
| SAUSA300_1458 | *-* | Lactoylglutathione lyase (EC 4.4.1.5) |  |  |  |  | -2.9 | -2.1 |  |  | -2.6 |  |  | -2.2 |
| SAUSA300_1461 | *-* | Hypothetical protein | 2.7 | 2.3 | 2.3 | 3.4 | 2.7 | 2.2 | 2.3 | 2.1 |  |  |  |  |
| SAUSA300_1462 | *-* | Integral membrane protein |  |  |  | 2.0 |  |  |  |  |  |  |  |  |
| SAUSA300_1463 | *-* | Hypothetical protein |  |  | 2.1 | 2.6 |  |  |  |  |  |  |  |  |
| SAUSA300_1470 | *-* | Dimethylallyltransferase (EC 2.5.1.1) / Geranyltranstransferase (EC 2.5.1.10) |  |  |  |  |  |  |  |  |  |  |  | -2.1 |
| SAUSA300_1479 | *-* | Hypothetical protein |  |  |  |  | -2.3 | -2.0 | -2.3 |  | -2.0 |  |  |  |
| SAUSA300_1480 | *-* | Secretory antigen precursor SsaA | -2.0 |  |  |  | -2.1 | -2.2 | -2.3 |  |  |  |  |  |
| SAUSA300_1492 | *-* | Hypothetical protein |  |  |  | 2.0 |  |  |  |  |  |  |  |  |
| SAUSA300_1504 | *-* | ComG operon protein 1 | -2.3 | -3.8 | -3.3 | -3.4 | -2.5 | -2.7 | -2.8 | -2.6 | -2.3 | -2.8 | -2.8 |  |
| SAUSA300_1510 | *-* | 5-formyltetrahydrofolate cyclo-ligase (EC 6.3.3.2) |  |  | 2.1 |  |  |  |  |  |  |  |  |  |
| SAUSA300_1513 | *-* | Superoxide dismutase (EC 1.15.1.1) |  |  |  |  | -2.3 |  |  |  |  |  |  |  |
| SAUSA300_1514 | *zur* | Zinc-specific metalloregulatory protein |  |  |  |  |  |  |  | -2.1 |  |  |  |  |
| SAUSA300_1515 | *-* | High-affinity zinc uptake system membrane protein ZnuB |  |  |  | -2.3 |  |  | -2.0 | -2.2 |  |  |  |  |
| SAUSA300_1516 | *-* | High-affinity zinc uptake system ATP-binding protein ZnuC |  |  |  | -2.3 |  |  |  | -2.1 |  |  |  |  |
| SAUSA300_1517 | *-* | Endonuclease IV (EC 3.1.21.2) |  |  |  | -2.0 |  | -2.0 | -2.1 | -2.0 | -2.1 | -2.3 | -2.6 | -2.9 |
| SAUSA300_1518 | *-* | ATP-dependent RNA helicase |  |  |  | -2.3 |  |  | -2.2 |  |  |  | -2.1 | -2.1 |
| SAUSA300_1519 | *-* | NIF3-related protein |  |  | -2.2 | -2.1 |  | -2.2 | -2.2 |  | -2.5 | -2.0 |  | -2.2 |
| SAUSA300_1520 | *-* | Hypothetical cytosolic protein |  | -2.9 | -6.3 | -4.5 |  | -4.4 | -5.2 | -4.2 | -4.2 | -3.7 | -3.7 | -3.8 |
| SAUSA300_1521 | *rpoD* | RNA polymerase sigma factor RpoD |  |  | -2.2 | -2.3 |  | -2.2 | -2.3 | -2.3 | -2.5 | -2.5 | -2.6 | -2.7 |
| SAUSA300_1522 | *dnaG* | DNA primase (EC 2.7.7.-) |  | -3.5 | -4.2 | -3.6 |  | -2.7 | -3.2 | -3.1 | -3.8 | -3.9 | -3.4 | -3.4 |
| SAUSA300_1524 | *-* | CBS domain containing protein |  |  | 2.1 | 2.1 |  |  |  |  |  |  |  |  |
| SAUSA300_1525 | *glyS* | Glycyl-tRNA synthetase (EC 6.1.1.14) |  |  |  |  |  |  |  | 2.0 |  |  |  |  |
| SAUSA300_1532 | *-* | Siderophore-mediated iron transport protein |  |  |  |  |  |  |  |  |  |  | 2.1 |  |
| SAUSA300_1536 | *-* | tRNA 2-methylthioadenosine synthase homolog |  |  |  |  |  |  |  |  |  |  |  | -2.1 |
| SAUSA300_1538 | *prmA* | Ribosomal protein L11 methyltransferase (EC 2.1.1.-) |  |  |  |  |  |  |  | -2.4 |  |  |  |  |
| SAUSA300_1539 | *dnaJ* | Chaperone protein DnaJ |  |  |  | -2.4 |  |  | -2.3 | -2.8 |  |  |  |  |
| SAUSA300_1540 | *dnaK* | Chaperone protein DnaK |  | 2.5 | 2.3 |  |  |  |  |  |  |  |  |  |
| SAUSA300_1542 | *hrcA* | Heat-inducible transcription repressor HrcA |  | 2.1 | 2.3 |  |  |  |  |  |  |  |  | 2.4 |
| SAUSA300_1543 | *-* | Oxygen-independent coproporphyrinogen-III oxidase (EC 1.3.99.22) | 2.0 |  | -2.6 | -2.5 |  |  | -2.4 | -2.9 | -2.3 | -2.5 | -3.4 | -3.6 |
| SAUSA300_1545 | *rpsT* | SSU ribosomal protein S20P | 2.2 |  |  |  |  |  |  |  |  |  |  |  |
| SAUSA300_1546 | *holA* | DNA polymerase III, delta subunit (EC 2.7.7.7) |  | -3.4 | -3.3 | -3.9 |  | -2.6 | -3.2 | -3.8 | -3.3 | -3.9 | -4.1 | -4.0 |
| SAUSA300_1547 | *-* | COME operon protein 3 | -2.2 | -3.5 | -4.2 | -3.1 | -2.8 | -3.3 | -3.8 | -3.2 | -2.7 | -3.0 | -3.2 | -2.7 |
| SAUSA300_1548 | *-* | ComE operon protein 2 |  |  |  | 2.5 |  |  |  |  |  |  |  |  |
| SAUSA300_1551 | *-* | iojap protein family |  |  |  |  |  |  |  |  |  |  | -2.0 | -2.1 |
| SAUSA300_1560 | *-* | Fic family protein | 3.7 | 5.4 | 3.2 | 6.2 | 5.0 | 7.3 | 5.8 | 7.5 | 5.4 | 6.1 | 5.4 | 5.2 |
| SAUSA300_1561 | *-* | Hypothetical membrane spanning protein |  |  | -4.0 | -4.7 | -3.9 |  | -2.7 |  | -2.2 |  |  |  |
| SAUSA300_1562 | *-* | Lactam utilization protein LamB |  |  | -3.6 | -3.7 | -2.8 |  |  |  |  |  |  |  |
| SAUSA300_1563 | *accC* | Biotin carboxylase (EC 6.3.4.14) |  |  | -3.0 | -3.1 | -2.5 |  |  |  |  |  |  |  |
| SAUSA300_1564 | *accB* | Biotin carboxyl carrier protein of acetyl-CoA carboxylase |  |  | -2.0 |  |  |  |  |  |  |  |  |  |
| SAUSA300_1565 | *-* | Regulator of kinase autophosphorylation inhibitor |  |  | -3.3 | -3.5 | -3.0 |  |  |  | -2.1 |  |  | -2.1 |
| SAUSA300_1566 | *-* | Kinase autophosphorylation inhibitor KipI |  |  | -3.6 | -4.0 | -2.8 |  |  |  | -3.3 | -3.0 | -2.6 | -2.8 |
| SAUSA300_1568 | *udk* | Uridine kinase (EC 2.7.1.48) |  |  |  |  |  | -2.0 |  | -2.1 |  |  |  |  |
| SAUSA300_1569 | *-* | Peptidase family U32 |  |  |  | -3.5 |  | -2.3 | -2.5 | -2.7 |  |  | -2.1 | -2.2 |
| SAUSA300_1570 | *-* | Peptidase family U32 |  | -2.3 | -2.5 | -3.5 |  | -2.5 | -3.0 | -2.9 | -2.5 | -2.4 | -2.7 | -3.3 |
| SAUSA300_1571 | *-* | O-methyltransferase (EC 2.1.1.-) |  | -3.3 | -3.5 | -3.3 |  | -2.2 | -2.8 | -2.5 | -2.5 | -2.7 | -2.5 | -2.6 |
| SAUSA300_1575 | *alaS* | Alanyl-tRNA synthetase (EC 6.1.1.7) | 2.0 |  |  |  |  |  |  |  |  |  |  |  |
| SAUSA300_1579 | *-* | Cysteine desulfurase (EC 2.8.1.7) / Selenocysteine lyase (EC 4.4.1.16) |  |  | -2.7 |  |  |  |  |  |  |  |  |  |
| SAUSA300_1580 | *-* | Luciferase-like monooxygenase (EC 1.14.-.-) | -2.0 |  | -2.6 | -2.0 | -4.1 | -3.2 | -3.5 | -3.3 |  |  |  |  |
| SAUSA300_1581 | *-* | Hypothetical protein | -3.1 | -2.6 |  | -2.4 | -10.7 | -5.6 | -5.4 | -5.4 | -8.5 | -8.0 | -4.2 | -2.9 |
| SAUSA300_1582 | *-* | Hypothetical protein | -4.6 | -2.7 | -2.6 | -3.8 | -14.5 | -8.7 | -7.7 | -8.2 | -11.3 | -9.6 | -5.5 | -3.6 |
| SAUSA300_1583 | *-* | Rrf2 family protein |  |  |  | 2.0 |  |  |  |  |  |  |  |  |
| SAUSA300_1585 | *-* | ThiF/MoeB family protein |  |  |  |  |  |  |  | -2.0 |  |  | -2.0 | -2.1 |
| SAUSA300_1586 | *aspS* | Aspartyl-tRNA synthetase (EC 6.1.1.12) | 2.3 |  |  |  | 3.4 |  |  |  |  |  |  |  |
| SAUSA300_1587 | *hisS* | Histidyl-tRNA synthetase (EC 6.1.1.21) | 2.6 |  |  |  | 4.0 | 2.3 |  |  | 2.5 |  |  |  |
| SAUSA300_1589 | *dtd* | D-tyrosyl-tRNA(Tyr) deacylase (EC 3.1.-.-) |  |  |  |  |  |  |  |  | -2.2 |  |  | -2.1 |
| SAUSA300_1590 | *-* | GTP pyrophosphokinase (EC 2.7.6.5) / Guanosine-3',5'-bis(Diphosphate) 3'-pyrophosphohydrolase (EC 3.1.7.2) |  |  |  |  |  |  |  | 2.3 |  |  |  |  |
| SAUSA300_1591 | *apt* | Adenine phosphoribosyltransferase (EC 2.4.2.7) |  |  |  |  |  |  |  |  |  |  | -2.0 | -2.1 |
| SAUSA300_1592 | *recJ* | Single-stranded-DNA-specific exonuclease recJ (EC 3.1.-.-) |  |  | -2.2 | -2.4 |  |  |  |  |  |  | -2.1 |  |
| SAUSA300_1595 | *tgt* | Queuine tRNA-ribosyltransferase (EC 2.4.2.29) | 2.6 |  |  |  |  |  |  |  |  |  |  |  |
| SAUSA300_1596 | *queA* | S-adenosylmethionine:tRNA ribosyltransferase-isomerase (EC 5.-.-.-) | 2.4 |  |  |  |  |  |  |  |  |  |  |  |
| SAUSA300_1600 | *obgE* | GTP-binding protein CgtA (probably involved in DNA repair) |  |  |  |  |  |  |  |  |  |  | -2.0 | -2.2 |
| SAUSA300_1606 | *-* | Hypothetical protein |  |  |  |  |  |  |  |  |  | 2.0 | 2.5 |  |
| SAUSA300_1608 | *radC* | DNA repair protein RadC |  | -2.9 | -2.2 | -2.4 | -2.3 | -2.5 | -2.1 |  |  |  |  |  |
| SAUSA300_1612 | *tag* | DNA-3-methyladenine glycosylase (EC 3.2.2.20) |  |  |  | 2.7 |  |  |  | 2.6 |  |  |  |  |
| SAUSA300_1613 | *-* | AbrB protein |  |  |  |  |  |  |  |  |  | 2.3 | 3.3 | 2.7 |
| SAUSA300_1620 | *engB* | GTP-binding protein YihA |  | -2.4 | -2.5 | -2.0 |  |  | -2.1 | -2.4 |  | -2.5 | -3.1 | -3.2 |
| SAUSA300_1621 | *clpX* | ATP-dependent endopeptidase clp ATP-binding subunit ClpX |  |  |  |  |  |  |  | -2.3 |  |  |  |  |
| SAUSA300_1622 | *tig* | Trigger factor, ppiase (EC 5.2.1.8) |  |  |  |  |  |  |  | -2.3 |  |  |  |  |
| SAUSA300_1624 | *-* | Hypothetical cytosolic protein |  |  |  |  |  |  |  |  |  |  | -2.1 |  |
| SAUSA300_1625 | *rplT* | LSU ribosomal protein L20P |  |  |  |  |  |  |  | -2.2 |  |  |  |  |
| SAUSA300_1626 | *rpmI* | LSU ribosomal protein L35P |  |  |  | -2.5 |  |  |  | -2.9 |  |  |  |  |
| SAUSA300_1627 | *infC* | Bacterial Protein Translation Initiation Factor 3 (IF-3) |  |  |  | -2.9 |  |  | -2.0 | -3.0 |  |  | -2.0 |  |
| SAUSA300_1628 | *lysP* | Lysine-specific permease |  |  |  |  |  |  |  | -2.5 |  |  |  |  |
| SAUSA300_1629 | *thrS* | Threonyl-tRNA synthetase (EC 6.1.1.3) |  | 2.2 | 2.1 |  |  |  | 2.4 |  |  |  |  |  |
| SAUSA300_1631 | *-* | Replication initiation and membrane attachment protein |  |  |  |  |  |  |  | 2.2 |  |  |  |  |
| SAUSA300_1632 | *nrdR* | Putative regulatory protein |  |  |  |  |  |  |  | 2.1 |  |  |  |  |
| SAUSA300_1633 | *gap* | Glyceraldehyde 3-phosphate dehydrogenase (EC 1.2.1.12) | -3.2 |  |  | -4.3 |  | -2.9 | -3.2 | -4.0 |  |  |  | 2.2 |
| SAUSA300_1640 | *icd* | Isocitrate dehydrogenase [NADP] (EC 1.1.1.42) |  |  |  | -2.5 | -2.2 |  |  |  | 2.4 | 3.9 | 3.5 | 2.5 |
| SAUSA300_1641 | *gltA* | Citrate synthase (EC 2.3.3.1) |  |  |  | -2.7 | -2.2 |  | -2.1 | -2.3 |  | 3.2 | 2.3 |  |
| SAUSA300_1645 | *pfkA* | 6-phosphofructokinase (EC 2.7.1.11) |  | 2.3 | 2.1 | 3.0 |  | 2.9 | 2.8 | 2.7 | 2.4 |  |  |  |
| SAUSA300_1653 | *-* | Metal-dependent hydrolase (EC 3.-.-.-) |  |  |  |  | -2.2 |  |  |  |  |  |  |  |
| SAUSA300_1654 | *-* | Xaa-Pro aminopeptidase (EC 3.4.11.9) |  |  |  | 2.9 |  | 2.4 | 2.1 | 2.5 | 2.1 | 2.2 | 2.2 | 2.0 |
| SAUSA300_1655 | *ald* | Alanine dehydrogenase (EC 1.4.1.1) |  |  | 2.4 |  |  |  |  | -2.1 |  |  |  |  |
| SAUSA300_1656 | *-* | Universal stress protein family | -3.0 |  |  |  |  |  |  | 2.0 |  | 2.0 | 2.7 | 3.1 |
| SAUSA300_1658 | *-* | Adenine-specific methyltransferase (EC 2.1.1.72) |  | -3.1 | -3.2 | -4.3 |  | -3.6 | -5.5 | -8.2 |  |  | -2.7 | -3.3 |
| SAUSA300_1660 | *-* | Hypothetical membrane spanning protein | 2.9 |  |  | -2.2 |  |  |  |  |  |  |  |  |
| SAUSA300_1661 | *thiI* | Thiamine biosynthesis protein thiI | 2.8 |  |  | -2.8 |  |  |  | -2.0 |  |  |  |  |
| SAUSA300_1662 | *-* | Cysteine desulfurase (EC 2.8.1.7) / Selenocysteine lyase (EC 4.4.1.16) | 2.8 | -2.1 | -3.4 | -3.3 |  |  |  | -2.3 |  |  |  |  |
| SAUSA300_1666 | *rpsD* | SSU ribosomal protein S4P |  |  |  |  |  |  |  | -2.3 |  |  |  |  |
| SAUSA300_1668 | *-* | Osmotically inducible protein C | -2.1 |  |  |  | -2.8 |  |  | -2.1 |  |  |  |  |
| SAUSA300_1669 | *-* | Serine--pyruvate aminotransferase (EC 2.6.1.51) | -4.4 |  | -4.3 |  |  | 4.2 | 6.3 | 16.5 | 2.8 | 5.7 | 7.4 | 5.5 |
| SAUSA300_1670 | *serA* | D-3-phosphoglycerate dehydrogenase (EC 1.1.1.95) | -3.4 |  | -3.4 |  |  | 3.7 | 5.2 | 13.3 | 2.3 | 4.1 | 5.4 | 4.1 |
| SAUSA300_1671 | *-* | Hpr(Ser) kinase (EC 2.7.1.-) / phosphatase (EC 3.1.3.-) |  |  | -2.7 |  |  |  |  | 2.6 |  |  |  |  |
| SAUSA300_1672 | *nagE* | PTS system, N-acetylglucosamine-specific IIBC component (EC 2.7.1.69) | 2.6 | 2.5 | 2.4 | 2.7 | 2.9 | 2.6 | 2.2 |  | 2.9 |  |  |  |
| SAUSA300_1673 | *-* | 1-acyl-sn-glycerol-3-phosphate acyltransferase (EC 2.3.1.51) | 3.8 | 3.1 | 3.1 | 3.5 | 4.1 | 4.1 | 3.2 | 2.2 | 4.6 | 2.6 |  |  |
| SAUSA300_1674 | *-* | Endopeptidase DegP (EC 3.4.21.-) |  |  |  | 2.6 |  | 2.2 |  |  | 2.7 | 2.7 | 2.2 |  |
| SAUSA300_1675 | *tyrS* | Tyrosyl-tRNA synthetase (EC 6.1.1.1) | 4.4 | 4.5 | 5.3 | 2.2 | 3.9 | 3.1 | 2.5 |  | 2.9 | 2.3 |  |  |
| SAUSA300_1676 | *sgtA* | Penicillin-binding protein |  |  |  | 2.1 |  |  |  |  |  |  |  |  |
| SAUSA300_1677 | *-* | Fibronectin-binding protein |  |  |  |  |  |  |  | 2.6 |  |  |  |  |
| SAUSA300_1678 | *fhs* | Formate--tetrahydrofolate ligase (EC 6.3.4.3) |  |  |  |  |  | 2.4 |  |  |  |  |  |  |
| SAUSA300_1679 | *acsA* | Acetyl-coenzyme A synthetase (EC 6.2.1.1) |  |  |  | -2.1 |  |  |  |  | 3.0 | 7.8 | 7.4 | 4.1 |
| SAUSA300_1680 | *acuA* | Acetoin utilization protein acuA (EC 2.3.1.-) |  |  |  |  |  |  |  |  |  | 2.3 | 2.8 | 2.6 |
| SAUSA300_1681 | *acuC* | Acetoin utilization acuC protein | -2.3 |  |  |  |  |  |  |  |  |  | 2.1 |  |
| SAUSA300_1683 | *-* | 3-deoxy-7-phosphoheptulonate synthase (EC 2.5.1.54) / Chorismate mutase (EC 5.4.99.5) |  |  |  |  |  |  |  | 2.6 |  |  |  |  |
| SAUSA300_1684 | *-* | Hypothetical exported protein | -3.4 | -2.6 |  | -2.6 | -4.7 | -4.6 | -5.2 | -5.0 | -6.2 | -5.7 | -3.9 | -3.0 |
| SAUSA300_1685 | *-* | General stress protein |  |  |  |  | -2.2 |  | -2.1 | -2.3 | -2.0 | -2.1 |  |  |
| SAUSA300_1686 | *murC* | UDP-N-acetylmuramate--alanine ligase (EC 6.3.2.8) |  |  |  |  |  |  |  | -2.5 |  |  |  |  |
| SAUSA300_1687 | *-* | Cell division protein FtsK |  |  |  | -2.3 |  |  | -2.1 | -3.5 |  |  | -2.2 | -2.2 |
| SAUSA300_1688 | *-* | tRNA binding domain protein |  |  |  | -2.3 |  |  | -2.3 | -3.5 |  |  |  |  |
| SAUSA300_1689 | *-* | Hypothetical cytosolic protein |  |  |  | -2.6 |  |  | -2.6 | -4.4 |  | -2.0 | -2.2 |  |
| SAUSA300_1690 | *-* | Thioredoxin | -2.2 |  |  |  | -2.7 | -2.0 | -2.2 | -2.2 |  |  |  |  |
| SAUSA300_1694 | *trmB* | tRNA (m(7)G46) methyltransferase (EC 2.1.1.33) |  |  | -2.1 |  |  |  |  |  |  |  |  |  |
| SAUSA300_1697 | *-* | Xaa-His dipeptidase (EC 3.4.13.3) |  |  |  |  |  |  |  | 2.2 |  |  |  |  |
| SAUSA300_1698 | *-* | Hypothetical protein | -3.2 | -2.7 | -2.1 | -2.8 | -4.4 | -4.6 | -4.3 | -4.1 | -5.4 | -5.1 | -3.2 | -2.8 |
| SAUSA300_1702 | *-* | Extracellular matrix binding protein |  |  |  |  |  |  |  | 2.0 |  |  |  |  |
| SAUSA300_1703 | *-* | Rhodanese-related sulfurtransferases | 2.0 | 2.2 |  |  |  |  |  |  |  |  |  |  |
| SAUSA300_1704 | *leuS* | Leucyl-tRNA synthetase (EC 6.1.1.4) | 2.1 | 2.6 |  |  | 2.3 | 2.2 |  |  | 2.3 |  |  |  |
| SAUSA300_1705 | *-* | Multidrug resistance protein |  |  | -2.2 | -2.3 |  |  | -2.2 | -3.0 |  |  | -2.2 | -2.3 |
| SAUSA300_1706 | *-* | Radical SAM superfamily protein |  |  | -3.1 |  |  |  |  |  |  |  |  |  |
| SAUSA300_1707 | *-* | SAM-dependent methyltransferase (EC 2.1.-.-) |  |  | -2.0 |  |  |  |  |  | -2.3 |  |  | -2.4 |
| SAUSA300_1708 | *rot* | Staphylococcal accessory regulator |  |  |  |  |  |  |  |  | 2.3 | 2.0 |  |  |
| SAUSA300_1711 | *putA* | Proline dehydrogenase (EC 1.5.99.8) | -7.1 |  |  | -5.2 | -4.6 | -4.2 | -4.2 | -6.1 |  |  |  |  |
| SAUSA300_1712 | *ribH* | 6,7-dimethyl-8-ribityllumazine synthase (EC 2.5.1.9) | -3.4 |  | -2.6 | -2.2 |  |  |  |  |  | 3.0 | 3.1 | 2.2 |
| SAUSA300_1713 | *ribBA* | GTP cyclohydrolase II (EC 3.5.4.25) / 3,4-dihydroxy-2-butanone-4-phosphate synthase (EC 4.1.2.-) | -4.2 | -2.2 | -3.8 | -3.0 |  |  |  |  |  | 2.5 | 2.5 |  |
| SAUSA300_1714 | *ribE* | Riboflavin synthase alpha chain (EC 2.5.1.9) | -3.9 | -2.9 | -5.5 | -3.4 |  |  |  |  |  | 2.2 |  |  |
| SAUSA300_1715 | *ribD* | Diaminohydroxyphosphoribosylaminopyrimidine deaminase (EC 3.5.4.26) / 5-amino-6-(5-phosphoribosylamino)uracil reductase (EC 1.1.1.193) | -3.3 | -2.6 | -3.9 | -2.0 |  |  |  |  |  | 2.4 | 2.1 |  |
| SAUSA300_1716 | *-* | Hypothetical exported protein |  |  |  |  | -2.3 | -2.2 | -2.6 | -2.3 | -2.1 | -2.6 | -2.7 | -2.8 |
| SAUSA300_1717 | *arsR* | Arsenical resistance operon repressor | 2.8 |  |  |  | 2.5 |  |  |  |  |  |  |  |
| SAUSA300_1718 | *arsB* | Arsenical pump membrane protein | 3.0 |  |  |  | 2.6 |  |  |  |  |  |  |  |
| SAUSA300_1719 | *arsC* | Arsenate reductase (EC 1.20.4.1) | 2.3 |  |  |  | 2.0 |  |  |  |  |  |  |  |
| SAUSA300_1720 | *-* | Peptidoglycan endo-beta-N-acetylglucosaminidase (EC 3.2.1.-) | 2.7 |  |  |  |  |  |  |  |  |  |  |  |
| SAUSA300_1723 | *-* | Hypothetical protein |  |  | -2.7 |  |  |  |  |  | -2.4 |  |  |  |
| SAUSA300_1724 | *-* | Hypothetical membrane spanning protein |  | -2.6 |  |  |  | -2.1 |  |  | -2.1 | -2.3 |  |  |
| SAUSA300_1725 | *-* | Transaldolase (EC 2.2.1.2) | -3.5 |  | -2.4 |  | -3.3 |  | -2.3 | -2.5 |  |  |  |  |
| SAUSA300_1731 | *pckA* | Phosphoenolpyruvate carboxykinase [ATP] (EC 4.1.1.49) | -6.2 | -3.5 | -2.3 | -8.4 | -3.0 | -7.8 | -6.9 | -8.3 |  |  |  |  |
| SAUSA300_1738 | *-* | Hypothetical protein |  | -2.1 | -2.2 |  |  |  |  | 2.6 |  |  |  |  |
| SAUSA300_1739 | *-* | Endonuclease (EC 3.1.-.-) |  |  |  |  |  | -2.6 | -2.7 | -2.6 |  |  |  |  |
| SAUSA300_1740 | *-* | Hypothetical protein |  |  |  |  |  | -2.8 | -2.6 | -2.1 |  |  |  |  |
| SAUSA300_1741 | *-* | Hypothetical protein |  |  |  | -2.3 |  | -2.4 | -2.6 | -4.1 |  |  |  |  |
| SAUSA300_1742 | *-* | Hypothetical protein |  |  |  |  |  | -2.2 | -2.4 | -2.9 |  | -2.5 | -2.7 | -2.1 |
| SAUSA300_1743 | *-* | Hypothetical protein |  | -2.3 |  | -2.3 |  | -2.4 | -3.0 | -3.2 |  | -2.9 | -2.9 | -2.4 |
| SAUSA300_1744 | *-* | Hypothetical protein |  | -2.6 |  | -2.5 |  | -3.0 | -3.6 | -3.9 | -2.9 | -4.0 | -4.8 | -4.9 |
| SAUSA300_1750 | *-* | Hypothetical protein | 2.2 |  |  |  | 2.2 |  |  |  |  |  |  |  |
| SAUSA300_1753 | *splF* | Serine protease (EC 3.4.21.-) | -4.7 | -6.5 | -2.8 |  |  | -4.8 | -4.7 | -3.4 | -2.5 | -3.8 | -4.1 | -2.5 |
| SAUSA300_1754 | *splE* | Serine protease (EC 3.4.21.-) | -5.0 | -7.9 | -3.8 |  |  | -5.3 | -4.8 | -3.7 | -3.0 | -4.9 | -4.4 | -2.8 |
| SAUSA300_1755 | *splD* | Serine protease (EC 3.4.21.-) | -4.1 | -4.2 | -3.1 |  |  | -4.2 | -3.7 | -3.1 | -2.3 | -3.3 | -3.1 |  |
| SAUSA300_1756 | *splC* | Serine protease (EC 3.4.21.-) | -4.5 | -7.3 | -3.7 |  |  | -6.7 | -5.8 | -4.7 | -3.0 | -4.9 | -3.8 | -2.8 |
| SAUSA300_1757 | *splB* | Serine protease (EC 3.4.21.-) | -4.4 | -4.3 | -3.0 |  |  | -4.8 | -4.5 | -3.6 | -2.7 | -3.5 | -2.9 | -2.4 |
| SAUSA300_1758 | *splA* | Serine protease (EC 3.4.21.-) | -5.9 | -6.9 | -3.4 |  |  | -6.0 | -4.7 | -4.0 | -3.1 | -6.3 | -5.6 | -3.4 |
| SAUSA300_1759 | *-* | Hypothetical protein |  |  | 2.4 |  |  |  |  |  |  |  |  |  |
| SAUSA300_1760 | *epiG* | Epidermin resistance transmembrane protein | -3.9 | -2.2 |  | -2.2 | -4.9 | -4.0 | -3.5 | -3.5 | -3.0 | -3.0 |  |  |
| SAUSA300_1761 | *epiE* | Lantibiotic transport permease protein | -5.7 | -2.8 |  | -2.6 | -6.1 | -4.5 | -4.1 | -3.7 | -2.5 | -2.8 |  |  |
| SAUSA300_1762 | *epiF* | Lantibiotic transport ATP-binding protein | -7.8 | -2.8 |  | -3.5 | -8.5 | -5.6 | -5.8 | -5.6 | -3.8 | -4.6 | -2.0 |  |
| SAUSA300_1766 | *epiB* | Serine (threonine) dehydratase (lantibiotic biosynthesis) |  |  | 2.3 | 3.6 |  | 2.1 |  | 2.0 |  |  |  | 2.4 |
| SAUSA300_1767 | *epiA* | Lantibiotic gallidermin |  | -2.0 |  | 2.2 |  |  |  |  |  |  |  |  |
| SAUSA300_1768 | *lukD* | Hypothetical protein |  | -2.0 | -2.1 |  |  | -2.0 |  |  |  |  |  |  |
| SAUSA300_1769 | *lukE* | Leukocidin S subunit |  | -2.0 | -2.3 |  |  |  |  |  |  |  |  |  |
| SAUSA300_1781 | *hemG* | Protoporphyrinogen oxidase (EC 1.3.3.4) |  |  | 2.0 |  |  |  |  |  |  |  |  |  |
| SAUSA300_1787 | *-* | Adenosine 5'-monophosphoramidase |  |  |  | 2.4 |  |  |  | 2.2 |  |  |  |  |
| SAUSA300_1788 | *-* | Hypothetical protein | -2.6 |  |  |  | -3.3 | -2.4 | -2.4 |  | -3.0 | -2.4 |  |  |
| SAUSA300_1789 | *-* | Hypothetical protein |  |  | -2.7 |  |  |  |  |  |  |  |  |  |
| SAUSA300_1790 | *prsA* | Peptidyl-prolyl cis-trans isomerase (EC 5.2.1.8) | -2.1 |  |  |  |  |  |  |  |  |  |  |  |
| SAUSA300_1794 | *-* | Hypothetical protein |  |  | -2.4 |  |  |  |  |  |  |  |  |  |
| SAUSA300_1795 | *-* | Hypothetical cytosolic protein |  |  | 2.3 | 2.2 |  |  |  |  |  |  |  |  |
| SAUSA300_1796 | *-* | Hypothetical membrane associated protein |  |  | -3.7 | -4.1 |  | -3.0 | -3.6 | -3.0 | -3.7 | -3.6 | -3.1 | -3.2 |
| SAUSA300_1797 | *-* | Transcriptional regulator, PbsX family | 3.2 |  |  |  | 2.5 |  |  |  |  |  |  |  |
| SAUSA300_1799 | *-* | Two-component sensor protein YhcY (EC 2.7.3.-) |  |  |  | 2.3 |  |  |  | 2.3 |  |  |  |  |
| SAUSA300_1800 | *-* | Ribosomal large subunit pseudouridine synthase D (EC 4.2.1.70) |  |  |  |  |  |  |  |  |  |  | -2.0 |  |
| SAUSA300_1801 | *fumC* | Fumarate hydratase (EC 4.2.1.2) |  | 4.0 | 5.0 | 2.4 | 2.6 | 2.9 | 2.8 | 2.6 | 3.7 | 3.1 | 2.8 | 2.2 |
| SAUSA300_1803 | *-* | Hypothetical protein |  |  |  |  | -2.6 | -2.3 | -2.0 | -2.1 |  |  |  |  |
| SAUSA300_1804 | *-* | Hypothetical protein | -2.6 |  |  |  | -3.2 | -2.4 | -2.5 | -2.3 | -2.9 | -2.6 |  |  |
| SAUSA300_1805 | *-* | 23S rRNA methyltransferase (EC 2.1.1.-) | 2.1 |  |  |  |  |  |  |  |  |  |  |  |
| SAUSA300_1806 | *-* | iron-sulfur cluster-binding protein | 2.0 |  |  |  |  |  |  |  |  |  |  |  |
| SAUSA300_1807 | *-* | Arginine transport ATP-binding protein ArtP | 2.5 |  | 2.7 |  |  |  |  |  |  |  | 2.0 |  |
| SAUSA300_1808 | *-* | Arginine-binding protein / Arginine transport system permease protein ArtQ | 2.8 |  | 2.7 |  |  |  |  |  |  |  | 2.4 |  |
| SAUSA300_1809 | *-* | Hypothetical membrane spanning protein |  |  |  |  |  |  |  | 2.1 |  |  |  |  |
| SAUSA300_1846 | *-* | Integral membrane protein |  |  |  | 2.3 | 2.2 | 2.3 | 2.3 | 2.3 |  |  |  | 2.0 |
| SAUSA300_1847 | *-* | Multidrug/protein/lipid ABC transporter family, ATP-binding and permease protein |  | -2.8 | -4.5 | -5.8 | -2.8 | -5.4 | -7.0 | -7.5 | -4.1 | -4.7 | -5.3 | -4.7 |
| SAUSA300_1848 | *-* | Hypothetical cytosolic protein |  |  |  | -2.2 |  |  |  | -2.2 |  |  |  |  |
| SAUSA300_1849 | *mutY* | A/G-specific adenine DNA glycosylase (EC 3.2.2.-) |  |  | -2.4 |  |  |  |  |  |  |  |  |  |
| SAUSA300_1854 | *recX* | Regulatory protein RecX | -2.1 |  |  |  | -2.2 |  |  | -2.1 |  |  |  | -2.7 |
| SAUSA300_1857 | *-* | Hypothetical protein | 2.8 | 2.3 | 2.5 | 2.4 | 2.4 |  |  |  | 2.1 |  |  |  |
| SAUSA300_1858 | *-* | Thioredoxin-like oxidoreductases | 2.1 |  |  |  |  |  |  |  |  |  |  |  |
| SAUSA300_1862 | *-* | Protein tyrosine phosphatase (EC 3.1.3.48) | -2.6 |  |  |  | -3.3 | -2.4 | -2.4 | -2.2 | -2.8 | -2.9 | -2.3 | -2.3 |
| SAUSA300_1864 | *-* | Ribonuclease BN (EC 3.1.-.-) | -3.0 | -2.7 |  | -3.0 | -7.6 | -5.2 | -5.4 | -5.7 | -4.7 | -5.7 | -4.0 | -3.2 |
| SAUSA300_1865 | *vraR* | Two-component response regulator YvqC |  |  |  |  |  |  |  |  | -2.0 |  |  | -2.0 |
| SAUSA300_1869 | *map* | Methionine aminopeptidase (EC 3.4.11.18) |  |  |  | 2.0 |  |  |  |  |  |  |  |  |
| SAUSA300_1870 | *-* | Integral membrane protein | 2.2 | 2.6 | 2.3 | 2.8 | 2.6 | 2.6 | 2.7 | 3.2 | 2.4 | 2.0 |  |  |
| SAUSA300_1871 | *-* | Hypothetical protein |  |  | -2.3 |  |  |  |  |  |  |  |  |  |
| SAUSA300_1874 | *-* | Ferritin |  | -2.0 |  | 2.6 | -10.0 | -29.8 | -30.5 | -30.9 | -11.5 | -5.6 | -3.4 | -8.5 |
| SAUSA300_1875 | *-* | DNA polymerase III alpha subunit (EC 2.7.7.7) |  |  | -2.4 |  |  | -2.0 | -2.4 | -2.9 |  |  |  |  |
| SAUSA300_1876 | *-* | DNA polymerase IV (EC 2.7.7.7) |  | -2.1 |  | -2.5 |  | -2.8 | -2.8 | -2.9 |  |  |  |  |
| SAUSA300_1877 | *-* | Permease |  |  |  |  |  |  | 2.5 | 5.1 |  | 2.6 | 2.9 | 2.8 |
| SAUSA300_1878 | *rumA* | 23S rRNA m(5)U 1939 methyltransferase (EC 2.1.1.-) |  | -2.4 | -2.3 | -3.2 |  | -2.7 | -3.5 | -4.7 |  | -2.5 | -2.9 | -3.1 |
| SAUSA300_1883 | *putP* | Sodium/proline symporter |  |  |  |  | 2.5 |  |  | 2.6 |  |  |  |  |
| SAUSA300_1888 | *-* | cytosolic protein containing cobalamin binding site |  |  |  |  |  |  |  |  | 2.4 |  |  |  |
| SAUSA300_1889 | *purB* | Adenylosuccinate lyase (EC 4.3.2.2) |  |  |  |  |  | 2.9 |  |  | 3.1 | 2.1 |  |  |
| SAUSA300_1890 | *-* | Staphopain (EC 3.4.22.-) | -4.5 | -3.7 | -2.1 |  | 2.2 |  |  |  |  |  | -3.1 | -2.8 |
| SAUSA300_1893 | *nadE* | NH(3)-dependent NAD(+) synthetase (EC 6.3.5.1) |  | 2.6 | 2.7 | 3.3 |  |  |  | 2.1 |  |  |  |  |
| SAUSA300_1894 | *-* | Nicotinate phosphoribosyltransferase (EC 2.4.2.11) |  | 2.8 | 2.8 | 3.6 |  | 2.4 | 2.3 | 2.4 |  |  |  |  |
| SAUSA300_1895 | *-* | Nitric-oxide synthase (EC 1.14.13.39) |  |  | 2.6 | 3.4 |  |  | 2.2 | 2.6 |  |  |  |  |
| SAUSA300_1896 | *pheA* | Prephenate dehydratase (EC 4.2.1.51) |  |  | 2.3 | 3.6 |  |  | 2.0 | 2.5 |  |  |  |  |
| SAUSA300_1897 | *-* | Transporter, Divalent Anion:Sodium Symporter family | -2.7 |  |  |  | -2.6 | -2.7 | -2.3 |  | -2.7 |  |  |  |
| SAUSA300_1901 | *aldA2* | Aldehyde dehydrogenase (NAD(P)+) (EC 1.2.1.5) | -2.6 |  |  |  | -2.8 |  | -2.2 |  |  |  |  |  |
| SAUSA300_1905 | *-* | Choloylglycine hydrolase |  |  |  | 2.4 |  |  |  | 2.1 |  |  |  |  |
| SAUSA300_1909 | *-* | Thioredoxin |  | 2.4 | 2.6 | 3.5 |  |  |  |  |  | 2.5 | 2.5 |  |
| SAUSA300_1910 | *-* | Hypothetical protein |  |  |  |  | -2.0 | -2.1 | -2.2 | -2.0 | -2.1 |  |  | -2.3 |
| SAUSA300_1911 | *-* | ABC transporter ATP-binding protein |  |  |  |  |  |  |  |  | -2.2 |  |  | -2.3 |
| SAUSA300_1912 | *-* | Hypothetical protein |  |  |  |  |  |  |  | -2.1 |  |  |  | -2.6 |
| SAUSA300_1915 | *-* | Hypothetical protein |  |  |  |  |  |  |  |  |  |  | -2.4 | -4.0 |
| SAUSA300_1918 | *-* | Sphingomyelin phosphodiesterase (EC 3.1.4.12) |  | -2.0 |  | 2.0 |  | -2.7 | -2.3 | -2.1 |  |  |  |  |
| SAUSA300_1919 | *-* | Hypothetical protein |  | -2.9 | -2.4 |  |  |  |  |  | -2.2 |  |  |  |
| SAUSA300_1920 | *chs* | Chemotaxis-inhibiting protein CHIPS | -2.5 | -2.2 |  |  | 2.2 |  | 3.3 | 3.5 |  |  | 2.6 | 3.7 |
| SAUSA300_1921 | *-* | N-acetylmuramoyl-L-alanine amidase (EC 3.5.1.28) |  |  | -3.0 | -2.3 |  | -2.1 | -2.1 | -2.3 | -3.0 | -2.6 | -2.9 | -2.8 |
| SAUSA300_1922 | *sak* | Staphylokinase precursor |  |  | 2.4 | 4.2 |  |  |  |  |  |  |  |  |
| SAUSA300_1925 | *-* | Phage protein |  |  |  |  |  |  |  | -2.0 |  |  |  |  |
| SAUSA300_1930 | *-* | Hypothetical protein |  |  |  |  |  |  |  | -2.0 |  |  |  |  |
| SAUSA300_1931 | *-* | Hypothetical protein |  |  |  |  |  |  |  | -2.5 |  |  |  |  |
| SAUSA300_1932 | *-* | Hypothetical protein |  |  |  |  | -2.1 |  | -2.0 | -2.8 | -2.2 | -2.8 | -2.9 | -2.3 |
| SAUSA300_1933 | *-* | Hypothetical protein |  |  |  |  |  |  |  | -2.1 |  |  |  |  |
| SAUSA300_1934 | *-* | prophage pi2 protein 39 |  |  |  |  |  | -2.4 | -2.2 | -2.9 |  | -2.5 | -2.6 | -2.2 |
| SAUSA300_1935 | *-* | Hypothetical cytosolic protein |  |  |  |  | -2.2 |  | -2.6 | -3.0 | -3.4 | -3.3 | -2.8 | -3.1 |
| SAUSA300_1936 | *-* | Phage protein |  |  |  |  | -2.1 | -2.0 | -2.3 | -2.8 | -2.1 | -2.7 | -2.3 |  |
| SAUSA300_1937 | *-* | Phage protein |  |  |  |  |  |  |  | -2.2 |  | -2.2 |  |  |
| SAUSA300_1938 | *-* | Major capsid protein |  |  |  |  | -2.4 | -2.1 | -2.4 | -3.2 |  | -2.1 | -2.5 |  |
| SAUSA300_1939 | *-* | ATP-dependent endopeptidase clp proteolytic subunit ClpP (EC 3.4.21.92) |  |  |  |  | -2.0 | -2.1 | -2.3 | -2.7 | -2.3 | -2.7 | -2.7 | -2.5 |
| SAUSA300_1943 | *-* | HNH endonuclease family protein |  |  |  |  |  |  |  |  |  | -2.1 | -2.5 |  |
| SAUSA300_1945 | *-* | Phage protein |  |  |  |  |  |  |  |  | -2.1 | -2.5 |  |  |
| SAUSA300_1949 | *dut* | Deoxyuridine 5'-triphosphate nucleotidohydrolase (EC 3.6.1.23) |  |  |  |  |  |  |  |  |  | -2.4 |  |  |
| SAUSA300_1951 | *-* | Phage protein |  |  |  |  |  |  |  |  | -2.2 | -2.7 | -2.1 |  |
| SAUSA300_1952 | *-* | Hypothetical protein |  |  |  |  |  |  |  |  | -2.4 | -2.7 | -2.3 |  |
| SAUSA300_1956 | *-* | Hypothetical protein |  |  |  |  |  | -2.0 |  |  |  | -2.1 |  |  |
| SAUSA300_1958 | *-* | Single-strand DNA binding protein |  |  |  |  |  | -2.2 |  |  |  | -2.1 |  |  |
| SAUSA300_1962 | *-* | Hypothetical cytosolic protein |  |  |  |  |  |  |  |  | -2.3 | -2.3 |  |  |
| SAUSA300_1963 | *-* | Hypothetical cytosolic protein |  |  |  |  |  |  |  |  |  | -2.0 |  |  |
| SAUSA300_1964 | *-* | Hypothetical protein |  |  |  |  |  | -2.2 | -2.0 |  | -2.3 | -2.3 | -2.0 |  |
| SAUSA300_1966 | *-* | Phage antirepressor protein |  |  |  |  | -2.0 | -2.4 | -2.2 |  | -2.3 | -2.6 | -2.3 | -2.2 |
| SAUSA300_1967 | *-* | Phage protein |  |  | -2.0 |  |  | -2.9 | -3.0 | -2.5 | -2.1 | -2.6 | -2.4 | -2.3 |
| SAUSA300_1969 | *-* | Phage transcriptional repressor | 4.3 | 8.6 | 7.2 | 4.7 | 5.1 | 5.2 | 5.3 | 5.1 | 5.3 | 4.9 | 4.7 | 4.3 |
| SAUSA300_1970 | *-* | DNA polymerase III alpha subunit (EC 2.7.7.7) | 5.1 | 9.4 | 7.9 | 5.5 | 6.7 | 6.2 | 6.2 | 6.0 | 6.7 | 5.7 | 5.5 | 5.3 |
| SAUSA300_1971 | *-* | Phage protein | 3.4 | 6.0 | 5.5 | 4.4 | 3.7 | 3.4 | 3.5 | 3.6 | 3.4 | 3.0 | 3.2 | 2.7 |
| SAUSA300_1972 | *int* | DNA integration/recombination/inversion protein | 3.0 |  | 2.0 |  | 3.6 |  |  |  |  |  |  |  |
| SAUSA300_1973 | *-* | Sphingomyelin phosphodiesterase (EC 3.1.4.12) | 2.9 | 2.3 | 2.9 |  | 3.3 |  |  |  |  |  |  |  |
| SAUSA300_1974 | *lukG* | Leukocidin F subunit | -5.6 |  |  | 3.9 |  |  | 2.7 | 3.5 |  |  | 4.5 | 5.4 |
| SAUSA300_1975 | *lukH* | Leukocidin S subunit | -5.7 |  |  | 4.6 |  | 2.3 | 3.8 | 4.4 |  |  | 4.9 | 6.3 |
| SAUSA300_1976 | *-* | Succinyl-diaminopimelate desuccinylase (EC 3.5.1.18) | -2.6 |  |  |  | -3.4 | -2.5 | -2.2 |  |  |  |  |  |
| SAUSA300_1977 | *-* | Tetracenomycin polyketide synthesis O-methyltransferase tcmP (EC 2.1.1.-) | 2.1 |  |  |  | 9.4 | 13.8 | 13.3 | 14.3 | 7.4 | 7.3 | 6.0 | 7.5 |
| SAUSA300_1978 | *-* | Ferrichrome-binding protein / Ferrioxamine B binding protein | 2.2 | 2.3 |  |  | 13.5 | 22.6 | 21.6 | 21.6 | 10.5 | 9.5 | 7.0 | 8.8 |
| SAUSA300_1979 | *-* | Potassium uptake protein KtrB | -2.4 |  |  |  |  |  |  |  |  |  |  |  |
| SAUSA300_1980 | *-* | Acetyltransferase (EC 2.3.1.-) |  |  |  |  | -2.1 | -2.1 |  |  | -2.3 | -2.1 |  | -2.0 |
| SAUSA300_1981 | *-* | Phage Terminase Small Subunit |  |  |  | -2.3 |  |  | -2.0 | -2.0 |  |  |  |  |
| SAUSA300_1982 | *groEL* | 60 kDa chaperonin GroeL |  | 4.3 | 3.7 |  | 2.3 | 2.3 |  |  | 2.9 | 2.7 | 2.9 | 3.3 |
| SAUSA300_1983 | *groES* | 10 kDa chaperonin GroeS |  | 3.3 | 3.2 |  | 2.3 | 2.2 |  |  | 3.0 | 2.4 | 2.4 | 3.2 |
| SAUSA300_1984 | *-* | CAAX amino terminal protease family |  | 2.0 |  |  |  |  |  |  |  |  |  |  |
| SAUSA300_1985 | *sdrH* | Hypothetical membrane associated protein |  |  |  | 2.0 |  | 2.3 | 2.9 | 6.0 |  |  | 2.1 |  |
| SAUSA300_1986 | *-* | Nitroreductase family protein |  |  | -2.6 |  |  |  |  |  |  |  |  |  |
| SAUSA300_1987 | *-* | Beta-ureidopropionase (EC 3.5.1.6) |  |  |  |  |  |  |  |  |  | 2.1 |  |  |
| SAUSA300_1988 | *hld* | Delta-lysin | -4.0 |  |  |  | -19.9 | -16.0 | -8.6 | -4.0 | -5.6 | -7.0 | -2.6 |  |
| SAUSA300_1989 | *agrB* | Accessory gene regulator protein B AgrB |  |  |  |  | -2.4 | -3.0 | -2.4 |  |  | -2.6 | -2.2 |  |
| SAUSA300_1990 | *agrD* | Autoinducing peptide AgrD |  |  |  |  |  | -2.6 | -2.1 |  |  | -2.7 | -2.4 |  |
| SAUSA300_1991 | *agrC* | Sensory transduction histidine kinase AgrC (EC 2.7.3.-) | -2.1 |  |  |  | -2.9 | -3.8 | -2.8 |  | -2.0 | -2.7 | -2.2 |  |
| SAUSA300_1992 | *agrA* | Accessory gene regulator protein A AgrA | -2.5 |  |  |  | -4.5 | -3.2 | -2.6 |  | -2.1 | -2.5 |  |  |
| SAUSA300_1994 | *scrB* | Sucrose-6-phosphate hydrolase (EC 3.2.1.26) |  |  |  |  |  |  |  |  | -2.1 |  |  | -2.4 |
| SAUSA300_1996 | *amt* | Hypothetical protein |  |  |  |  |  | 2.0 | 2.2 | 4.8 |  |  | 2.3 | 2.5 |
| SAUSA300_1997 | *-* | Transcriptional regulatory protein | -2.7 | 2.1 |  | 3.3 | -5.9 | -7.5 | -4.3 | -2.6 | -6.6 | -5.3 | -5.4 | -5.9 |
| SAUSA300_1998 | *-* | putative transport system permease protein |  | 2.9 | 2.3 | 5.8 | -3.0 | -3.4 | -2.2 |  | -2.4 | -2.0 | -2.2 |  |
| SAUSA300_2000 | *vga* | ABC transporter ATP-binding protein | 2.5 | 2.2 | 2.1 | 2.1 | 2.7 | 2.3 | 2.0 | 2.2 |  |  |  |  |
| SAUSA300_2001 | *-* | DNA mismatch repair protein MutS |  |  | 2.0 | 2.3 | 2.5 | 3.1 | 2.2 | 2.0 | 3.6 | 2.6 |  |  |
| SAUSA300_2002 | *-* | O-sialoglycoprotein endopeptidase (EC 3.4.24.57) |  |  |  |  |  | -2.1 | -2.1 | -2.1 | -2.2 | -2.2 | -2.5 | -2.3 |
| SAUSA300_2003 | *rimI* | Ribosomal-protein-S18-alanine acetyltransferase (EC 2.3.1.128) |  |  |  |  |  |  |  |  |  |  | -2.3 | -2.2 |
| SAUSA300_2004 | *-* | Non-proteolytic protein, peptidase family M22 |  |  | -2.3 |  |  |  | -2.0 |  |  |  | -2.1 |  |
| SAUSA300_2005 | *-* | ATP/GTP hydrolase |  | -2.3 | -2.9 |  |  |  |  |  | -2.1 | -2.0 | -2.0 |  |
| SAUSA300_2006 | *ilvD* | Dihydroxy-acid dehydratase (EC 4.2.1.9) |  |  |  | -2.3 |  | 8.1 | 10.7 | 20.3 | 7.0 | 11.1 | 10.6 | 8.8 |
| SAUSA300_2007 | *ilvB* | Acetolactate synthase large subunit (EC 2.2.1.6) | -2.9 | -2.6 | -2.8 | -3.2 |  | 6.6 | 8.2 | 15.3 | 5.0 | 8.3 | 9.5 | 7.8 |
| SAUSA300_2008 | *ilvN* | Acetolactate synthase small subunit (EC 2.2.1.6) | -3.3 | -2.8 | -2.8 | -3.5 |  | 7.9 | 9.9 | 18.0 | 7.0 | 11.8 | 14.8 | 13.0 |
| SAUSA300_2009 | *ilvC* | Ketol-acid reductoisomerase (EC 1.1.1.86) / 2-dehydropantoate 2-reductase (EC 1.1.1.169) | -4.6 | -4.3 | -5.9 | -6.2 |  | 5.7 | 7.0 | 13.2 | 3.8 | 6.9 | 9.3 | 9.2 |
| SAUSA300_2010 | *leuA* | 2-isopropylmalate synthase (EC 2.3.3.13) | -5.4 | -4.1 | -5.7 | -6.3 |  | 5.2 | 7.1 | 14.9 | 4.3 | 8.2 | 12.3 | 12.4 |
| SAUSA300_2011 | *leuB* | 3-isopropylmalate dehydrogenase (EC 1.1.1.85) | -5.4 | -4.5 | -5.6 | -5.7 |  | 5.0 | 6.8 | 13.4 | 3.1 | 6.3 | 9.6 | 8.4 |
| SAUSA300_2012 | *leuC* | 3-isopropylmalate dehydratase large subunit (EC 4.2.1.33) | -5.5 | -5.4 | -6.1 | -6.1 |  | 4.0 | 5.3 | 11.2 | 2.7 | 5.8 | 8.5 | 8.1 |
| SAUSA300_2013 | *leuD* | 3-isopropylmalate dehydratase small subunit (EC 4.2.1.33) | -4.6 | -5.0 | -6.1 | -5.6 |  | 3.7 | 5.2 | 10.9 | 3.0 | 6.4 | 10.2 | 9.6 |
| SAUSA300_2014 | *ilvA* | Threonine dehydratase (EC 4.3.1.19) | -4.7 | -5.2 | -6.5 | -5.7 | -2.0 | 3.2 | 4.2 | 8.8 | 2.1 | 4.4 | 6.9 | 6.9 |
| SAUSA300_2020 | *-* | Metallopeptidase, SprT family (EC 3.4.24.-) |  |  |  |  |  |  | -2.0 | -2.2 |  |  | -2.5 | -2.5 |
| SAUSA300_2021 | *-* | Transcription accessory protein (S1 RNA binding domain) |  |  | -2.1 | -2.1 |  |  | -2.0 | -2.4 | -2.5 | -3.0 | -3.9 | -4.6 |
| SAUSA300_2022 | *rpoF* | RNA polymerase sigma-B factor | -2.8 | -2.7 | -2.7 | -3.2 | -5.2 | -4.3 | -4.3 | -3.9 | -5.5 | -5.5 | -5.0 | -4.8 |
| SAUSA300_2023 | *rsbW* | Anti-sigma B factor | -2.7 | -2.5 | -2.7 | -2.9 | -4.6 | -4.0 | -3.6 | -3.3 | -5.0 | -5.2 | -4.4 | -4.1 |
| SAUSA300_2024 | *rsbV* | Anti-sigma B factor antagonist | -2.1 | -2.3 | -2.2 | -2.1 | -3.4 | -3.1 | -2.8 | -2.8 | -3.2 | -3.6 | -2.8 | -2.4 |
| SAUSA300_2026 | *-* | PEMK-like protein |  |  |  | 2.3 |  |  |  |  |  |  |  |  |
| SAUSA300_2033 | *kdpB* | Potassium-transporting ATPase B chain (EC 3.6.3.12) |  |  |  |  |  |  |  | 2.7 |  |  |  |  |
| SAUSA300_2034 | *kdpA* | Potassium-transporting ATPase A chain (EC 3.6.3.12) |  |  |  |  |  |  |  | 3.3 |  |  |  |  |
| SAUSA300_2035 | *kdpD* | Sensor protein KdpD (EC 2.7.3.-) |  |  |  |  |  |  | 2.5 | 4.5 |  |  |  |  |
| SAUSA300_2036 | *kdpE* | Two-component response regulator kdpE | -2.1 |  |  |  |  |  |  | 3.2 |  |  |  |  |
| SAUSA300_2037 | *-* | ATP-dependent RNA helicase |  | -4.0 | -4.5 | -7.9 |  | -2.9 | -4.1 | -7.3 |  | -2.6 | -3.7 | -3.4 |
| SAUSA300_2042 | *-* | Hypothetical protein | 2.2 |  |  |  | 2.0 |  |  |  |  |  |  |  |
| SAUSA300_2043 | *-* | Hypothetical cytosolic protein | 2.4 |  |  |  | 2.2 |  |  |  |  |  |  |  |
| SAUSA300_2045 | *-* | Metal dependent hydrolase |  |  |  |  |  |  |  |  |  |  | -2.3 | -2.9 |
| SAUSA300_2046 | *oxaA* | 60 kDa inner membrane protein YidC |  |  |  |  |  |  |  | -2.1 |  |  | -2.0 |  |
| SAUSA300_2047 | *thiE* | Thiamin-phosphate pyrophosphorylase (EC 2.5.1.3) |  |  |  |  |  | 3.0 | 4.2 | 7.0 |  | 2.9 | 4.2 | 5.5 |
| SAUSA300_2048 | *thiM* | Hydroxyethylthiazole kinase (EC 2.7.1.50) |  |  |  |  |  | 2.4 | 3.2 | 4.8 |  | 2.3 | 2.9 | 3.5 |
| SAUSA300_2049 | *thiD* | Phosphomethylpyrimidine kinase (EC 2.7.4.7) / Hydroxymethylpyrimidine kinase (EC 2.7.1.49) |  |  |  |  |  | 2.8 | 4.1 | 6.2 |  | 2.8 | 3.6 | 4.2 |
| SAUSA300_2050 | *-* | Transcriptional activator tenA |  |  |  |  | 2.1 | 4.1 | 5.6 | 8.2 |  | 3.0 | 4.0 | 5.1 |
| SAUSA300_2051 | *-* | SceD precursor | 3.1 |  |  |  |  |  |  | 2.1 |  |  |  |  |
| SAUSA300_2052 | *-* | Single-strand DNA binding protein |  | -2.6 | -2.5 |  | -2.2 |  |  |  | -2.5 | -2.8 | -2.2 |  |
| SAUSA300_2053 | *-* | Hypothetical protein |  |  | 2.1 | 2.6 |  | 2.5 | 2.2 |  | 2.0 |  |  |  |
| SAUSA300_2054 | *fabZ* | (3R)-hydroxyacyl-[acyl carrier protein] dehydratase (EC 4.2.1.60) |  |  |  |  | -2.1 | -2.8 | -2.7 | -2.5 | -3.4 | -3.3 | -3.5 | -3.7 |
| SAUSA300_2055 | *murA* | UDP-N-acetylglucosamine 1-carboxyvinyltransferase (EC 2.5.1.7) | -2.0 | -2.2 | -2.3 | -2.7 | -2.4 | -3.3 | -3.3 | -3.0 | -3.1 | -3.3 | -3.2 | -3.1 |
| SAUSA300_2056 | *-* | Hypothetical membrane associated protein | -3.2 | -3.8 | -3.8 | -4.3 | -3.7 | -4.8 | -4.9 | -4.8 | -5.5 | -5.7 | -3.7 | -3.0 |
| SAUSA300_2057 | *atpC* | ATP synthase epsilon chain (EC 3.6.3.14) | 2.1 |  |  |  | 2.2 |  |  |  | 2.1 |  |  |  |
| SAUSA300_2059 | *atpG* | ATP synthase gamma chain (EC 3.6.3.14) | 2.0 |  |  |  | 2.3 |  |  |  | 2.1 |  |  |  |
| SAUSA300_2060 | *atpA* | ATP synthase alpha chain (EC 3.6.3.14) |  |  |  |  | 2.0 |  |  |  |  |  |  |  |
| SAUSA300_2064 | *atpB* | ATP synthase A chain (EC 3.6.3.14) |  |  |  |  | 2.0 |  |  |  |  |  |  |  |
| SAUSA300_2071 | *-* | Peptide release factor-glutamine N5-methyltransferase (EC 2.1.1.-) |  |  |  | -2.4 |  | -2.0 | -2.1 | -2.4 |  | -2.1 | -2.6 | -2.5 |
| SAUSA300_2072 | *prfA* | Bacterial peptide chain release factor 1 (RF-1) |  |  |  | -2.9 |  |  | -2.1 | -2.3 |  |  | -2.4 | -2.2 |
| SAUSA300_2073 | *tdk* | Thymidine kinase (EC 2.7.1.21) |  | -2.3 |  | -2.6 |  |  |  | -2.2 |  | -2.1 | -2.7 | -2.5 |
| SAUSA300_2076 | *-* | Aldehyde dehydrogenase (EC 1.2.1.3) | -4.3 |  |  |  | -8.2 | -6.1 | -5.3 | -4.1 | -4.5 | -2.9 |  |  |
| SAUSA300_2077 | *-* | Transcriptional regulator, MarR family |  |  |  |  |  | -2.1 | -2.2 |  |  |  |  |  |
| SAUSA300_2080 | *-* | Hypothetical protein |  | 2.1 |  | 2.2 | 2.4 | 2.2 |  |  |  |  |  |  |
| SAUSA300_2081 | *pyrG* | CTP synthase (EC 6.3.4.2) | 2.2 |  |  | -3.7 | 2.2 |  |  | -4.4 |  |  |  |  |
| SAUSA300_2083 | *-* | Acetyltransferase (EC 2.3.1.-) |  |  |  | -2.0 |  |  |  | -2.4 |  |  |  |  |
| SAUSA300_2084 | *coaA* | Pantothenate kinase (EC 2.7.1.33) |  |  |  |  |  |  |  | -2.4 |  |  |  |  |
| SAUSA300_2087 | *-* | Peptidase family M20/M25/M40 |  | 2.1 |  |  |  |  |  | 2.6 |  |  |  |  |
| SAUSA300_2088 | *luxS* | Hypothetical protein |  |  | 2.1 | 2.5 |  |  |  |  |  |  |  |  |
| SAUSA300_2089 | *pdp* | Thymidine phosphorylase (EC 2.4.2.4) |  |  | 3.0 |  |  |  |  |  |  | 2.0 | 2.8 | 2.3 |
| SAUSA300_2091 | *deoD* | Purine nucleoside phosphorylase (EC 2.4.2.1) |  | 2.0 | 2.3 | 2.5 |  |  |  |  | 2.1 |  |  |  |
| SAUSA300_2092 | *dps* | Non-specific DNA-binding protein Dps / Iron-binding ferritin-like antioxidant protein / Ferroxidase (EC 1.16.3.1) | -5.3 | -5.3 | -4.2 | -3.2 | -8.1 | -6.1 | -4.7 | -4.2 | -2.7 |  |  |  |
| SAUSA300_2093 | *-* | Hypothetical protein |  |  |  | 3.1 |  |  | 2.4 | 2.9 |  |  | 2.4 | 2.9 |
| SAUSA300_2096 | *manA* | Mannose-6-phosphate isomerase (EC 5.3.1.8) |  |  |  |  |  |  |  |  |  |  | -2.0 | -2.0 |
| SAUSA300_2097 | *-* | Putative NAD-dependent dehydrogenase | -2.7 |  |  |  | -14.2 | -4.8 | -3.8 | -3.9 | -4.3 | -4.8 | -2.3 |  |
| SAUSA300_2098 | *arsR* | Hypothetical protein | 3.3 | 2.9 | 2.2 | 3.8 | 3.8 | 5.7 | 5.9 | 7.8 | 2.8 | 4.6 | 4.4 | 4.4 |
| SAUSA300_2099 | *-* | Cobalt-zinc-cadmium resistance protein CzcD | 2.2 | 2.7 | 2.1 | 2.3 | 2.8 | 3.9 | 4.4 | 6.0 | 2.1 | 3.1 | 3.7 | 3.5 |
| SAUSA300_2100 | *-* | Lytic regulatory protein |  |  |  |  |  |  |  | -2.0 |  |  | -2.1 |  |
| SAUSA300_2103 | *-* | Hypothetical protein |  | -2.0 | -2.4 | -2.2 |  |  |  |  |  |  |  |  |
| SAUSA300_2104 | *glmS* | Glucosamine--fructose-6-phosphate aminotransferase [isomerizing] (EC 2.6.1.16) |  |  |  |  |  |  |  |  | -3.3 | -2.1 |  |  |
| SAUSA300_2105 | *mtlF* | PTS system, mannitol-specific IIBC component (EC 2.7.1.69) | -15.0 | -22.7 | -20.0 | -23.7 | -4.5 | -11.7 | -10.2 | -13.9 | -6.8 | -9.6 | -3.3 |  |
| SAUSA300_2106 | *-* | Transcription antiterminator, BglG family / PTS system, mannitol (Cryptic)-specific IIA component (EC 2.7.1.69) | -13.9 | -9.8 | -6.2 | -11.1 | -6.7 | -12.1 | -10.9 | -14.1 | -7.9 | -15.2 | -5.0 | -2.4 |
| SAUSA300_2107 | *mtlA* | PTS system, mannitol-specific IIA component (EC 2.7.1.69) | -8.0 | -5.3 | -3.8 | -6.6 | -7.1 | -10.5 | -9.3 | -11.2 | -6.1 | -11.3 | -5.4 | -2.9 |
| SAUSA300_2108 | *mtlD* | Mannitol-1-phosphate 5-dehydrogenase (EC 1.1.1.17) | -5.9 | -4.8 | -3.4 | -5.4 | -5.8 | -8.5 | -7.7 | -9.1 | -3.7 | -6.4 | -3.8 |  |
| SAUSA300_2109 | *fmtB* | Hypothetical protein |  |  |  |  |  |  |  | 2.3 |  |  |  |  |
| SAUSA300_2110 | *fmtB* | Methicillin resistance protein |  |  |  | 2.2 | 3.1 | 2.8 | 2.8 | 3.4 | 2.1 |  | 2.2 | 2.2 |
| SAUSA300_2114 | *rocF* | Arginase (EC 3.5.3.1) |  |  |  |  |  |  |  |  |  | 2.3 |  |  |
| SAUSA300_2126 | *-* | Multidrug resistance protein B |  | -2.4 | -2.5 | -2.2 | -3.2 | -3.9 | -3.7 | -3.2 | -3.0 | -3.1 | -2.5 | -2.5 |
| SAUSA300_2127 | *-* | Hypothetical protein |  | -2.5 | -2.5 | -2.2 | -2.6 | -3.5 | -3.9 | -3.9 | -2.8 | -2.4 | -2.7 | -2.9 |
| SAUSA300_2128 | *-* | Permease |  |  |  | -2.0 |  | -2.2 | -2.3 |  |  | -2.1 | -2.2 | -2.5 |
| SAUSA300_2129 | *-* | Conserved membrane protein (hemolysin III homolog) |  |  |  |  |  |  |  | -2.0 |  |  |  |  |
| SAUSA300_2130 | *-* | UDP-N-acetylglucosamine pyrophosphorylase (EC 2.7.7.23) |  |  |  |  |  |  |  |  |  |  | -2.0 | -2.1 |
| SAUSA300_2131 | *-* | Predicted membrane-bound metal-dependent hydrolase |  |  |  | -2.0 | -2.1 | -2.3 | -2.2 | -2.1 | -2.0 | -2.6 | -2.1 | -2.2 |
| SAUSA300_2132 | *-* | Hypothetical cytosolic protein | -2.9 |  |  |  |  | -2.7 | -2.2 | -3.7 |  |  |  | 2.2 |
| SAUSA300_2133 | *-* | Hypothetical membrane spanning protein | 7.3 | 2.5 | 4.7 |  | 5.7 |  |  |  | 2.7 | 2.0 | 2.0 |  |
| SAUSA300_2134 | *htsC* | Iron(III) dicitrate transport system permease protein |  | 2.1 |  |  | 6.4 | 6.3 | 6.9 | 9.3 | 5.1 | 5.7 | 4.7 | 5.1 |
| SAUSA300_2135 | *htsB* | Ferrichrome transport system permease protein | 2.2 | 3.0 |  |  | 12.1 | 12.5 | 12.5 | 16.2 | 8.8 | 10.0 | 7.4 | 7.1 |
| SAUSA300_2136 | *htsA* | Iron(III) dicitrate-binding protein | 4.1 | 5.3 | 3.1 |  | 18.7 | 22.7 | 21.7 | 21.3 | 24.6 | 22.0 | 15.6 | 16.0 |
| SAUSA300_2137 | *-* | Hypothetical cytosolic protein |  |  |  |  | 2.3 | 4.4 | 5.4 | 6.6 |  | 3.0 | 3.4 | 4.6 |
| SAUSA300_2138 | *-* | Amino-acid citrate synthetase (EC 6.-.-.-) |  |  |  |  | 2.5 | 4.8 | 6.0 | 7.8 |  | 3.2 | 3.6 | 4.7 |
| SAUSA300_2139 | *-* | Macrolide-efflux protein | 2.1 | 2.3 |  |  | 15.2 | 32.2 | 38.9 | 49.5 | 10.7 | 19.9 | 19.7 | 27.2 |
| SAUSA300_2140 | *-* | Siderophore synthase (EC 6.-.-.-) |  | 2.1 |  |  | 2.7 | 4.7 | 5.6 | 7.5 |  | 2.8 | 2.6 | 2.8 |
| SAUSA300_2142 | *asp23* | Alkaline shock protein | -2.2 |  |  |  | -24.9 | -4.0 | -3.1 | -2.5 | -2.2 |  |  |  |
| SAUSA300_2143 | *-* | Small integral membrane protein | -2.3 |  |  |  | -29.5 | -5.0 | -3.6 | -2.6 |  |  |  |  |
| SAUSA300_2144 | *-* | Hypothetical protein | -3.7 | -2.0 |  |  | -45.1 | -7.7 | -5.3 | -4.2 | -2.8 | -2.5 |  |  |
| SAUSA300_2145 | *-* | Glycine betaine transporter | -17.5 | -9.8 | -11.6 | -12.0 | -105.1 | -58.7 | -50.1 | -37.0 | -43.4 | -31.7 | -14.3 | -12.3 |
| SAUSA300_2147 | *-* | Quinone oxidoreductase (EC 1.6.5.5) | -2.3 |  |  |  | -2.7 |  | -2.1 | -2.2 |  |  |  |  |
| SAUSA300_2148 | *-* | putative acyltransferases and hydrolases with the alpha/beta hydrolase fold | -2.5 |  |  |  | -2.5 | -2.2 | -2.3 | -2.4 |  |  |  |  |
| SAUSA300_2152 | *lacD* | Tagatose-bisphosphate aldolase (EC 4.1.2.40) |  |  |  |  |  |  |  |  |  |  |  | 2.1 |
| SAUSA300_2153 | *lacC* | Tagatose-6-phosphate kinase (EC 2.7.1.144) |  |  |  |  |  |  |  |  |  |  |  | 2.2 |
| SAUSA300_2157 | *-* | Sir2 family protein |  |  |  |  |  |  |  |  | 2.0 | 2.1 |  |  |
| SAUSA300_2158 | *-* | Hypothetical protein |  |  |  |  |  |  |  |  | -2.0 |  |  |  |
| SAUSA300_2159 | *-* | Morphine 6-dehydrogenase (EC 1.1.1.218) | -2.5 |  | -2.1 |  | -3.7 |  | -2.0 |  |  |  |  |  |
| SAUSA300_2160 | *-* | Transcriptional regulator, MerR family | -2.2 |  | -3.3 | -2.1 | -3.2 | -2.2 | -2.4 | -2.7 |  |  |  |  |
| SAUSA300_2162 | *-* | Peptidoglycan-specific endopeptidase, M23 family |  | 2.4 | 3.0 | 2.4 |  | 2.2 |  |  |  |  |  |  |
| SAUSA300_2163 | *-* | Hydrolase (HAD superfamily) |  | 2.6 | 3.3 | 2.7 |  | 2.8 | 2.4 |  | 2.0 |  |  |  |
| SAUSA300_2164 | *-* | Outer membrane protein | -10.8 | -6.1 | -2.4 | -3.9 | -35.1 | -12.5 | -9.8 | -7.4 | -2.4 | -2.8 |  |  |
| SAUSA300_2165 | *budA* | Alpha-acetolactate decarboxylase (EC 4.1.1.5) |  | 2.1 | 3.3 | 5.4 |  | -2.2 | -2.6 | -2.7 | -2.6 | -3.1 | -3.1 |  |
| SAUSA300_2166 | *alsS* | Acetolactate synthase (EC 2.2.1.6) |  |  | 2.5 | 5.7 |  | -2.6 | -2.7 | -3.0 | -2.1 | -2.8 |  |  |
| SAUSA300_2171 | *rpsI* | Ssu ribosomal protein S9P |  |  |  | -2.0 |  |  |  | -2.3 |  |  |  |  |
| SAUSA300_2172 | *rplM* | Lsu ribosomal protein L13P |  |  |  | -2.0 |  |  |  | -2.1 |  |  |  |  |
| SAUSA300_2176 | *cbiO* | Cobalt transport ATP-binding protein cbiO |  |  | -2.2 |  |  |  |  |  |  |  |  |  |
| SAUSA300_2177 | *rplQ* | Lsu ribosomal protein L17P |  |  |  |  | 2.0 |  |  |  |  |  |  |  |
| SAUSA300_2180 | *rpsM* | Ssu ribosomal protein S13P |  |  |  |  | 2.1 |  |  |  |  |  |  |  |
| SAUSA300_2181 | *rpmJ* | Lsu ribosomal protein L36P | 2.3 |  |  |  | 2.6 |  |  |  | 2.5 |  |  |  |
| SAUSA300_2182 | *infA* | Bacterial protein translation initiation factor 1 (IF-1) | 2.1 |  |  |  | 2.1 |  |  |  |  |  |  |  |
| SAUSA300_2185 | *rplO* | Lsu ribosomal protein L15P | 2.0 |  |  |  |  |  |  |  |  |  |  |  |
| SAUSA300_2187 | *rpsE* | Ssu ribosomal protein S5P | 2.1 |  |  |  | 2.1 |  |  |  |  |  |  |  |
| SAUSA300_2188 | *rplR* | Lsu ribosomal protein L18P | 2.2 |  |  |  | 2.2 |  |  |  |  |  |  |  |
| SAUSA300_2190 | *rpsH* | Ssu ribosomal protein S8P |  |  |  | -2.1 |  |  |  |  |  |  |  |  |
| SAUSA300_2191 | *rpsN* | Ssu ribosomal protein S14P |  |  |  | -2.2 |  |  |  |  |  |  |  |  |
| SAUSA300_2192 | *rplE* | Lsu ribosomal protein L5P |  |  |  | -2.1 |  |  |  |  |  |  |  |  |
| SAUSA300_2193 | *rplX* | Lsu ribosomal protein L24P |  |  |  | -2.2 |  |  |  |  |  |  |  |  |
| SAUSA300_2194 | *rplN* | Lsu ribosomal protein L14P |  |  |  | -2.2 |  |  |  |  |  |  |  |  |
| SAUSA300_2195 | *rpsQ* | Ssu ribosomal protein S17P |  |  |  | -2.1 |  |  |  |  |  |  |  |  |
| SAUSA300_2196 | *rpmC* | Lsu ribosomal protein L29P |  |  |  | -2.4 |  |  |  | -2.2 |  |  |  |  |
| SAUSA300_2197 | *rplP* | Lsu ribosomal protein L16P |  |  |  | -2.7 |  |  |  | -2.4 |  |  |  |  |
| SAUSA300_2198 | *rpsC* | Ssu ribosomal protein S3P |  |  |  | -2.3 |  |  |  | -2.1 |  |  |  |  |
| SAUSA300_2199 | *rplV* | Lsu ribosomal protein L22P |  |  |  | -2.9 |  |  |  | -2.9 |  |  |  | -2.2 |
| SAUSA300_2200 | *rpsS* | Ssu ribosomal protein S19P |  |  |  | -2.4 |  |  |  | -2.4 |  |  |  |  |
| SAUSA300_2202 | *rplW* | Lsu ribosomal protein L23P |  |  |  | -2.3 |  |  |  | -2.6 |  |  |  |  |
| SAUSA300_2203 | *rplD* | Lsu ribosomal protein L1E (= L4P) |  |  |  | -2.9 |  |  |  | -2.3 |  |  |  |  |
| SAUSA300_2204 | *rplC* | Lsu ribosomal protein L3P |  |  |  | -3.4 |  |  |  | -2.7 |  |  |  |  |
| SAUSA300_2205 | *rpsJ* | Ssu ribosomal protein S10P |  |  |  | -2.9 |  |  |  | -3.0 |  |  |  |  |
| SAUSA300_2206 | *-* | Hypothetical protein |  |  |  |  |  |  |  |  |  | 2.2 |  |  |
| SAUSA300_2207 | *-* | Guanine-hypoxanthine permease | 2.8 | 2.5 |  |  |  | 4.5 | 3.0 | 2.3 | 4.5 | 4.0 |  |  |
| SAUSA300_2209 | *-* | Hypothetical protein |  | 2.0 | 2.1 | 2.5 |  |  | 2.3 | 2.4 |  |  |  |  |
| SAUSA300_2213 | *-* | Acriflavin resistance plasma membrane protein |  |  |  |  |  | -2.4 | -3.2 | -3.7 | -3.2 | -3.7 | -4.3 | -4.0 |
| SAUSA300_2215 | *-* | Hypothetical cytosolic protein |  |  |  | 2.4 |  |  |  |  |  |  |  |  |
| SAUSA300_2217 | *-* | Transporter, MFS superfamily | 2.1 |  |  |  |  |  |  | -2.3 |  |  |  |  |
| SAUSA300_2225 | *moaC* | Molybdenum cofactor biosynthesis protein C |  |  |  | 2.3 |  |  |  | 2.2 |  |  |  |  |
| SAUSA300_2227 | *moeB* | Molybdopterin biosynthesis MoeB protein |  |  |  | 2.1 |  |  |  |  |  |  |  |  |
| SAUSA300_2228 | *modC* | Molybdenum transport ATP-binding protein modC |  |  |  | 2.4 |  |  |  |  |  |  |  |  |
| SAUSA300_2229 | *modB* | Molybdenum transport system permease protein modB |  |  | 2.0 | 2.8 |  |  | 2.0 | 2.1 |  |  |  |  |
| SAUSA300_2230 | *modA* | Molybdate-binding protein |  | 2.0 | 2.8 | 3.4 |  | 2.5 | 2.5 | 2.0 |  |  |  |  |
| SAUSA300_2231 | *fdhD* | FdhD protein (FdsC) |  |  | -2.5 |  | -3.1 | -3.4 | -4.6 | -5.0 |  |  |  |  |
| SAUSA300_2232 | *-* | Acetyltransferase (EC 2.3.1.-) |  |  |  |  | 2.6 | 5.6 | 7.7 | 7.9 |  | 4.3 | 5.0 | 5.0 |
| SAUSA300_2233 | *-* | BioY protein |  |  |  |  | 2.4 | 4.7 | 6.7 | 6.8 |  | 2.9 | 3.6 | 3.7 |
| SAUSA300_2234 | *-* | Inosine-uridine preferring nucleoside hydrolase (EC 3.2.2.1) | 2.4 | 2.1 |  |  | 3.3 | 2.5 | 2.2 | 2.2 |  |  |  |  |
| SAUSA300_2235 | *-* | Ferrichrome-binding protein / Ferrioxamine B binding protein / Aerobactin binding protein / Coprogen binding protein | 4.3 | 2.4 |  |  | 6.9 | 5.8 | 5.5 | 4.5 | 5.5 | 5.1 | 3.4 | 3.7 |
| SAUSA300_2236 | *-* | Acyl-CoA dehydrogenase, short-chain specific (EC 1.3.99.2) |  | 3.4 | 4.8 | 8.8 | -2.7 |  |  |  |  |  |  |  |
| SAUSA300_2247 | *-* | Staphylococcal accessory regulator | -3.2 | -3.0 | -3.7 | -3.8 | -2.9 | -3.6 | -3.2 | -3.0 | -3.6 | -3.7 | -4.3 | -3.6 |
| SAUSA300_2248 | *-* | Transcriptional regulator, AraC family | -3.9 | -4.2 | -6.0 | -4.8 | -2.9 | -4.2 | -4.2 | -3.7 | -5.5 | -5.5 | -5.7 | -5.1 |
| SAUSA300_2249 | *ssaA* | Secretory antigen precursor SsaA | 31.3 | 9.4 | 9.1 | 5.3 | 27.2 | 14.5 | 13.8 | 8.8 | 14.7 | 16.4 | 13.9 | 14.6 |
| SAUSA300_2250 | *nhaC* | Na+/H+ antiporter nhaC |  |  |  |  |  | -2.5 |  |  |  |  |  |  |
| SAUSA300_2252 | *-* | Hypothetical protein |  |  |  | 2.1 |  |  |  |  |  |  |  |  |
| SAUSA300_2253 | *ssaA* | Secretory antigen precursor SsaA | 5.6 | 2.1 |  | 6.4 | 2.7 | 10.3 | 13.9 | 22.6 | 5.0 | 10.8 | 14.3 | 16.0 |
| SAUSA300_2254 | *-* | Glyoxylate reductase (NADP+) (EC 1.1.1.79) / Glyoxylate reductase (NAD+) (EC 1.1.1.26) / Hydroxypyruvate reductase (EC 1.1.1.81) |  |  | 2.2 |  |  |  |  |  |  |  | 2.6 | 2.1 |
| SAUSA300_2256 | *-* | Peptidoglycan endo-beta-N-acetylglucosaminidase (EC 3.2.1.-) | 3.4 | 2.0 |  |  | 2.3 |  |  |  |  |  |  |  |
| SAUSA300_2257 | *-* | Hypothetical protein |  |  |  |  | -5.5 | -5.2 | -4.4 | -3.2 | -3.5 | -3.4 | -2.1 |  |
| SAUSA300_2258 | *-* | Formate dehydrogenase alpha chain (EC 1.2.1.2) | -2.7 |  |  |  | -6.2 | -5.8 | -4.9 | -3.7 | -3.5 | -3.6 |  |  |
| SAUSA300_2259 | *-* | Transcriptional regulator, LytR family |  |  |  |  |  | -2.3 | -2.5 | -2.3 |  |  |  |  |
| SAUSA300_2260 | *-* | Myo-inositol-1(or 4)-monophosphatase (EC 3.1.3.25) | -2.9 |  |  |  | -3.4 | -2.8 | -3.3 | -2.9 | -3.3 | -2.8 |  |  |
| SAUSA300_2261 | *-* | Transcriptional regulator, DeoR family | -2.1 |  |  |  | -2.1 |  |  |  |  |  |  |  |
| SAUSA300_2263 | *-* | Transposase |  |  |  |  |  |  |  |  | 2.8 |  |  | 2.8 |
| SAUSA300_2264 | *-* | Transcriptional regulator, RpiR family |  |  |  |  |  | -2.0 | -2.0 | -2.1 |  |  | -2.0 |  |
| SAUSA300_2267 | *-* | Phosphoglycolate phosphatase (EC 3.1.3.18) |  |  |  |  |  | 2.0 | 3.0 | 6.0 |  |  | 2.2 |  |
| SAUSA300_2268 | *-* | Transporter, Sodium/bile acid symporter family |  |  |  |  |  | 3.0 | 4.7 | 12.8 |  | 3.1 | 4.4 | 4.6 |
| SAUSA300_2269 | *-* | Hypothetical protein | 2.5 |  | 2.0 |  | 2.2 |  |  |  | 2.4 | 2.1 |  |  |
| SAUSA300_2271 | *-* | Transcriptional regulator, RpiR family |  |  |  |  |  |  |  | 2.4 |  |  |  |  |
| SAUSA300_2273 | *-* | Na+/H+ antiporter NhaC |  | 2.1 |  | 2.4 |  |  |  | 2.4 |  |  |  |  |
| SAUSA300_2274 | *-* | Hypothetical membrane spanning protein |  |  |  | 2.2 |  |  |  |  |  |  |  |  |
| SAUSA300_2275 | *-* | Hypothetical protein | -5.5 | -2.4 |  |  | -9.3 | -3.8 | -2.7 |  | -4.2 | -3.2 |  |  |
| SAUSA300_2276 | *-* | N-acyl-L-amino acid amidohydrolase (EC 3.5.1.14) |  |  |  |  |  | 2.2 | 2.5 | 4.2 |  | 2.2 | 2.0 | 2.1 |
| SAUSA300_2277 | *hutI* | Imidazolonepropionase (EC 3.5.2.7) |  |  |  |  |  |  |  |  |  |  | 2.6 | 2.9 |
| SAUSA300_2278 | *hutU* | Urocanate hydratase (EC 4.2.1.49) |  |  |  |  |  |  |  |  |  |  | 3.7 | 3.3 |
| SAUSA300_2279 | *-* | Transcriptional regulators, LysR family |  | 2.2 |  |  |  |  |  |  |  |  |  |  |
| SAUSA300_2281 | *hutG* | Formiminoglutamase (EC 3.5.3.8) | -4.4 |  |  |  | -7.1 | -3.3 | -3.2 | -3.1 | -2.0 | -2.3 |  |  |
| SAUSA300_2282 | *-* | Hypothetical protein | 3.6 | 2.2 |  | 2.5 | 2.9 | 2.8 | 3.2 | 5.0 |  |  | 2.1 | 2.3 |
| SAUSA300_2283 | *rpiA* | Ribose 5-phosphate isomerase (EC 5.3.1.6) | 2.2 | 2.3 | 3.0 | 3.4 |  | 2.5 | 2.6 | 2.2 |  |  |  |  |
| SAUSA300_2285 | *galM* | Aldose 1-epimerase (EC 5.1.3.3) | 2.4 |  |  | 2.4 | 2.3 | 3.1 | 3.1 | 2.9 | 2.6 | 2.1 |  |  |
| SAUSA300_2286 | *-* | Hypothetical membrane associated protein | 2.7 |  | 2.0 | 3.0 | 2.9 | 3.6 | 3.8 | 3.4 | 3.3 | 2.6 | 2.0 | 2.2 |
| SAUSA300_2287 | *-* | Sodium export permease protein |  |  |  |  |  |  |  |  |  |  |  | -2.1 |
| SAUSA300_2289 | *-* | Integral membrane protein |  |  |  |  |  |  |  | -2.4 |  |  | -2.3 | -2.8 |
| SAUSA300_2291 | *gltS* | Sodium/glutamate symport carrier protein | 2.8 |  |  |  | 4.4 |  |  |  |  |  |  |  |
| SAUSA300_2295 | *-* | Hypothetical protein |  |  |  | 2.2 |  |  |  |  |  |  |  |  |
| SAUSA300_2296 | *-* | Esterase (EC 3.1.1.-) |  | 2.8 | 2.3 | 3.5 |  | 2.4 | 2.4 | 2.6 |  |  |  |  |
| SAUSA300_2298 | *-* | Multidrug resistance protein B | 2.1 |  | -2.9 | -5.0 | -4.5 | -7.7 | -8.0 | -8.0 | -5.9 | -6.7 | -7.0 | -4.6 |
| SAUSA300_2299 | *-* | Multidrug resistance protein A | 2.2 | -2.4 | -4.8 | -6.6 | -5.7 | -9.0 | -13.2 | -14.2 | -9.8 | -12.5 | -9.7 | -5.4 |
| SAUSA300_2303 | *tcaR* | TcaR transcription regulator |  |  |  | -2.1 |  | -2.0 |  |  |  |  |  |  |
| SAUSA300_2305 | *-* | Transposase |  |  | -2.0 |  |  |  |  |  |  |  |  |  |
| SAUSA300_2306 | *-* | ABC transporter ATP-binding protein |  | -2.3 | -2.6 | -2.4 | -2.2 | -2.5 | -2.8 | -2.9 |  |  |  | 2.1 |
| SAUSA300_2307 | *-* | ABC transporter permease protein | -2.2 | -2.7 | -3.2 | -3.0 | -2.8 | -3.4 | -3.7 | -3.6 | -2.4 | -2.2 |  | 3.0 |
| SAUSA300_2308 | *-* | Two-component response regulator |  |  |  | 2.1 |  |  |  |  |  |  |  |  |
| SAUSA300_2310 | *-* | Transcriptional regulator |  |  |  |  |  | -2.1 |  |  | -2.9 | -2.5 |  |  |
| SAUSA300_2311 | *-* | Hypothetical membrane associated protein |  |  |  |  |  | -2.0 |  |  | -2.1 |  |  |  |
| SAUSA300_2313 | *-* | L-lactate permease | -4.3 |  | 2.1 | 2.2 | -3.1 |  | 2.0 | 2.0 | -2.8 | -2.8 |  |  |
| SAUSA300_2315 | *-* | membrane lipoprotein | -2.8 |  |  |  | -3.0 | -2.1 |  |  | -2.3 | -2.4 |  |  |
| SAUSA300_2316 | *-* | Protease synthase and sporulation negative regulatory protein PAI 1 |  | 3.1 | 2.4 | 5.8 |  | 2.7 | 2.9 | 2.9 |  |  |  |  |
| SAUSA300_2317 | *-* | Alcohol dehydrogenase (EC 1.1.1.1) |  |  | 2.0 |  |  |  |  |  |  |  |  |  |
| SAUSA300_2318 | *-* | Acetyltransferase, GNAT family |  |  |  | -2.7 |  | 2.8 | 2.7 |  |  | 2.4 |  |  |
| SAUSA300_2319 | *-* | Thioredoxin reductase (EC 1.8.1.9) |  |  |  | -2.6 |  | 3.1 | 3.1 | 2.3 | 2.3 | 3.0 | 2.1 | 2.5 |
| SAUSA300_2320 | *-* | Hypothetical protein |  |  | 2.5 | 3.1 |  |  | 2.2 | 2.0 |  |  |  |  |
| SAUSA300_2321 | *-* | Phage infection protein |  | -2.9 | -5.1 | -5.0 | -3.1 | -5.1 | -4.7 | -4.0 | -6.5 | -6.6 | -5.5 | -4.1 |
| SAUSA300_2322 | *-* | Transcriptional regulator, TetR family |  |  |  |  |  |  |  |  |  |  | -2.3 | -2.0 |
| SAUSA300_2324 | *-* | PTS system, sucrose-specific IIBC component (EC 2.7.1.69) | -2.2 |  |  | -2.3 | -5.5 | -13.5 | -8.1 | -8.9 | -7.4 | -7.6 | -5.7 | -4.6 |
| SAUSA300_2325 | *-* | Hypothetical protein |  |  |  | -2.6 |  | -4.7 | -4.4 | -5.0 | -3.0 |  |  |  |
| SAUSA300_2326 | *-* | Transcriptional regulator, AraC family |  |  | -2.2 |  |  |  |  |  |  |  |  |  |
| SAUSA300_2327 | *-* | General stress protein 26 | -3.1 |  |  |  | -5.4 | -3.8 | -3.4 | -2.6 | -2.7 | -2.6 |  |  |
| SAUSA300_2328 | *-* | Hypothetical protein | 2.3 | 2.0 | 2.3 | 3.2 | 2.5 | 2.5 | 2.4 |  |  |  |  |  |
| SAUSA300_2330 | *-* | Hypothetical protein |  |  |  | 2.5 |  |  |  |  |  |  |  |  |
| SAUSA300_2333 | *narK* | Nitrite extrusion protein | -2.4 | 4.0 | 2.2 |  | -4.3 |  |  |  | -5.6 | -7.9 |  |  |
| SAUSA300_2337 | *-* | Two-component response regulator | -3.0 |  |  | -2.2 | -4.0 | -3.0 | -2.4 | -2.7 | -6.1 | -6.3 | -2.7 | -2.5 |
| SAUSA300_2338 | *-* | Two component system histidine kinase (EC 2.7.3.-) | -2.9 |  |  |  | -3.4 | -2.3 |  | -2.0 | -3.9 | -4.1 |  |  |
| SAUSA300_2339 | *-* | Transcriptional regulator | -2.7 |  |  |  | -2.9 |  |  |  | -2.7 | -3.0 |  |  |
| SAUSA300_2340 | *narI* | Respiratory nitrate reductase gamma chain (EC 1.7.99.4) | -3.5 |  |  | -2.3 | -4.2 | -2.6 | -2.1 | -2.6 | -4.1 | -5.1 |  |  |
| SAUSA300_2341 | *narJ* | Respiratory nitrate reductase delta chain (EC 1.7.99.4) | -6.4 |  |  | -4.3 | -10.2 | -4.9 | -3.5 | -5.7 | -11.6 | -17.7 | -2.3 |  |
| SAUSA300_2342 | *narH* | Respiratory nitrate reductase beta chain (EC 1.7.99.4) | -7.0 |  |  | -3.9 | -11.2 | -4.6 | -3.2 | -4.7 | -8.6 | -16.9 |  |  |
| SAUSA300_2343 | *-* | Respiratory nitrate reductase alpha chain (EC 1.7.99.4) | -3.9 | 4.6 |  |  | -8.3 |  |  | -2.9 | -5.9 | -9.4 |  |  |
| SAUSA300_2344 | *-* | Uroporphyrin-III C-methyltransferase (EC 2.1.1.107) | -3.0 | 2.2 |  |  | -4.7 |  |  |  | -4.5 | -5.2 |  |  |
| SAUSA300_2345 | *nirD* | Nitrite reductase [NAD(P)H] small subunit (EC 1.7.1.4) | -2.7 | 3.2 | 2.3 |  | -3.7 |  |  |  | -3.1 | -4.3 |  |  |
| SAUSA300_2346 | *nirB* | Nitrite reductase [NAD(P)H] large subunit (EC 1.7.1.4) |  | 9.0 | 4.2 | 2.4 | -2.7 |  | 2.2 |  | -2.4 | -3.1 | 2.1 |  |
| SAUSA300_2347 | *nirR* | Sirohydrochlorin cobaltochelatase (EC 4.99.1.3) |  | 29.1 | 8.6 | 6.0 |  | 5.4 | 6.5 | 2.2 |  | -2.6 | 2.1 |  |
| SAUSA300_2349 | *-* | Nitrite transporter |  |  |  | 2.6 |  |  | 2.6 | 2.7 |  |  |  |  |
| SAUSA300_2350 | *-* | Hypothetical cytosolic protein |  |  |  |  |  |  |  | 2.1 |  |  |  |  |
| SAUSA300_2351 | *-* | Hypothetical protein | 5.5 |  |  |  | 4.9 |  |  |  | 2.4 |  |  |  |
| SAUSA300_2354 | *-* | Thiol:disulfide interchange protein DsbA | 2.4 | 2.1 |  |  |  | 2.0 |  | 2.3 |  |  |  |  |
| SAUSA300_2355 | *-* | Hypothetical protein | 2.7 | 2.2 |  | 2.2 |  | 2.3 | 2.2 | 2.4 |  |  |  |  |
| SAUSA300_2356 | *fmhA* | UDP-N-acetylmuramoylheptapeptide-glycine L-seryltransferase (EC 2.3.2.-) |  |  |  |  |  | 2.3 |  | 2.6 |  |  |  |  |
| SAUSA300_2357 | *-* | Cystine transport ATP-binding protein |  |  |  |  | -4.9 | -5.7 | -4.3 | -3.9 | -3.1 | -2.2 | -2.8 | -3.4 |
| SAUSA300_2358 | *-* | Cystine transport system permease protein |  |  |  |  | -4.1 | -4.7 | -3.7 | -3.6 | -2.5 | -2.0 | -2.6 | -3.1 |
| SAUSA300_2359 | *-* | Cystine-binding protein |  |  |  | 2.1 | -3.7 | -5.0 | -4.0 | -3.7 | -3.2 | -2.8 | -3.6 | -3.6 |
| SAUSA300_2360 | *-* | Multidrug resistance protein B | -3.0 | -2.3 | -3.7 | -3.5 | -3.2 | -4.1 | -4.1 | -4.1 | -4.6 | -5.0 | -3.0 | -2.7 |
| SAUSA300_2361 | *-* | Hypothetical protein | 3.1 |  |  |  | 2.3 |  |  |  |  |  | 2.2 | 3.6 |
| SAUSA300_2362 | *gpmA* | Phosphoglycerate mutase (EC 5.4.2.1) | -9.4 | -2.8 |  |  |  |  |  |  |  |  |  |  |
| SAUSA300_2364 | *sbi* | IgG-binding protein Sbi |  |  |  | 2.2 | 2.5 | 3.0 | 3.9 | 3.5 |  | 2.8 | 3.4 | 3.4 |
| SAUSA300_2365 | *hlgA* | Leukocidin S subunit |  |  | 2.5 |  |  |  | 3.0 | 3.4 | 78.5 | 95.1 | 144.9 | 136.3 |
| SAUSA300_2366 | *hlgC* | Leukocidin S subunit | -3.7 | -3.0 |  | 3.5 |  | -3.8 | -2.6 |  | 15.8 | 18.7 | 34.4 | 31.7 |
| SAUSA300_2367 | *hlgB* | Leukocidin F subunit | -2.5 | -2.2 |  | 2.8 |  | -2.4 |  |  | 14.5 | 17.5 | 33.8 | 37.1 |
| SAUSA300_2368 | *-* | BioX protein |  |  |  |  |  | 11.6 | 29.0 | 31.7 | 2.0 | 10.0 | 22.9 | 24.2 |
| SAUSA300_2369 | *-* | 6-carboxyhexanoate--CoA ligase (EC 6.2.1.14) |  |  |  |  |  | 19.2 | 51.0 | 59.3 | 2.1 | 13.8 | 33.9 | 33.5 |
| SAUSA300_2370 | *-* | 8-amino-7-oxononanoate synthase (EC 2.3.1.47) |  |  |  |  |  | 20.5 | 46.8 | 51.2 | 2.1 | 14.8 | 33.5 | 35.5 |
| SAUSA300_2371 | *bioB* | Biotin synthase (EC 2.8.1.6) |  |  |  |  |  | 26.2 | 56.3 | 63.5 | 2.6 | 14.0 | 30.4 | 35.6 |
| SAUSA300_2372 | *bioA* | Adenosylmethionine-8-amino-7-oxononanoate aminotransferase (EC 2.6.1.62) |  |  |  |  | 2.2 | 40.2 | 87.8 | 106.8 | 4.4 | 24.8 | 53.5 | 55.6 |
| SAUSA300_2373 | *bioD* | Dethiobiotin synthetase (EC 6.3.3.3) |  |  |  |  |  | 32.8 | 78.1 | 85.6 | 2.9 | 19.0 | 40.9 | 45.0 |
| SAUSA300_2374 | *-* | ABC transporter ATP-binding and permease protein |  |  |  |  |  | 3.4 | 5.9 | 9.9 |  |  |  | 2.6 |
| SAUSA300_2375 | *-* | ABC transporter ATP-binding and permease protein |  | -2.1 |  | -2.5 |  | 4.9 | 8.5 | 14.6 |  | 2.3 | 2.7 | 4.0 |
| SAUSA300_2376 | *-* | Monosaccharide translocase (flippase type) | -3.2 | -2.3 | -2.1 | -3.2 | -4.6 | -5.5 | -4.7 | -4.4 | -5.5 | -5.0 | -2.8 | -2.3 |
| SAUSA300_2379 | *-* | Hypothetical protein |  |  |  |  |  |  |  |  | -2.0 |  |  |  |
| SAUSA300_2380 | *-* | Transcriptional activator AarP |  | -2.2 |  | -2.6 | -2.1 | -2.6 | -2.6 | -2.4 | -3.5 | -3.1 | -2.8 | -2.3 |
| SAUSA300_2381 | *-* | Hypothetical protein | -2.3 |  |  |  | -2.6 |  |  | -2.5 |  |  |  |  |
| SAUSA300_2382 | *-* | Phosphoesterase |  |  | -2.2 |  | -2.4 | -2.5 | -2.3 |  | -2.3 |  |  |  |
| SAUSA300_2383 | *-* | D-serine/D-alanine/glycine transporter |  |  |  |  |  |  |  |  |  | 2.2 | 3.0 |  |
| SAUSA300_2384 | *-* | Na+/H+ antiporter NhaP |  |  |  | 2.3 |  |  |  | 2.3 |  |  |  |  |
| SAUSA300_2385 | *-* | Amino acid permease |  |  |  | 3.0 |  |  |  | 2.3 |  |  |  |  |
| SAUSA300_2386 | *-* | Beta-lactamase family protein |  |  |  |  |  |  |  |  |  |  |  |  |
| SAUSA300_2387 | *-* | dTDP-glucose 4,6-dehydratase (EC 4.2.1.46) |  |  | -3.2 | -2.3 |  | -2.3 | -2.5 | -3.2 |  |  | -2.2 | -2.3 |
| SAUSA300_2388 | *panE* | 2-dehydropantoate 2-reductase (EC 1.1.1.169) |  |  |  |  |  |  |  |  |  |  |  | -2.1 |
| SAUSA300_2389 | *-* | Multidrug resistance protein B |  |  | -2.3 |  |  |  |  |  |  |  |  |  |
| SAUSA300_2390 | *opuCd* | Glycine betaine transport system permease protein |  |  | 2.0 | 4.5 |  |  |  |  |  |  | 2.4 | 2.6 |
| SAUSA300_2391 | *opuCc* | Glycine betaine/carnitine/choline-binding protein |  |  | 2.2 | 5.5 |  |  |  |  |  |  | 2.2 |  |
| SAUSA300_2392 | *opuCb* | Glycine betaine/carnitine/choline transport system permease protein OpuCB |  |  | 2.7 | 7.3 |  |  |  | 2.3 |  |  | 2.9 | 2.6 |
| SAUSA300_2393 | *opuCa* | Glycine betaine transport ATP-binding protein |  |  | 3.2 | 9.6 |  |  |  | 3.0 |  | 2.1 | 3.6 | 3.1 |
| SAUSA300_2394 | *-* | 60 kDa chaperonin GroeL |  |  |  |  |  |  |  |  |  |  | -2.1 | -2.7 |
| SAUSA300_2395 | *-* | Amino acid permease |  |  | -3.1 |  | -2.8 | -4.0 | -3.5 | -2.9 | -3.2 | -3.7 | -2.9 | -2.8 |
| SAUSA300_2396 | *pnbA* | Para-nitrobenzyl esterase (EC 3.1.1.-) | -4.1 | -2.2 |  |  | -4.8 | -2.1 |  |  | -2.6 | -3.1 |  |  |
| SAUSA300_2397 | *-* | Chloramphenicol resistance protein |  | -2.1 | -2.2 | -2.8 |  | -4.2 | -3.8 | -2.8 | -4.7 | -4.8 | -3.2 | -3.0 |
| SAUSA300_2398 | *-* | ABC transporter permease protein | -5.6 | -2.6 |  | -3.3 | -22.6 | -11.3 | -8.6 | -7.6 | -11.4 | -10.6 | -4.4 | -3.4 |
| SAUSA300_2399 | *-* | ABC transporter ATP-binding protein | -7.9 | -2.8 |  | -4.3 | -26.0 | -13.7 | -10.3 | -10.2 | -11.0 | -12.5 | -4.7 | -3.9 |
| SAUSA300_2401 | *-* | RelE protein |  |  |  |  |  |  | 2.0 | 2.6 |  |  |  |  |
| SAUSA300_2402 | *-* | RelB protein | 2.6 | 2.5 |  | 2.2 | 2.7 |  | 2.3 | 2.9 |  | 2.1 |  |  |
| SAUSA300_2403 | *-* | Hypothetical protein |  | 2.2 | 2.2 | 2.4 |  |  |  |  |  |  |  |  |
| SAUSA300_2406 | *-* | Bicyclomycin resistance protein |  |  | 2.1 |  | 7.5 | 7.5 | 8.9 | 9.4 | 4.0 | 5.4 | 5.3 | 7.6 |
| SAUSA300_2407 | *-* | Nickel transport ATP-binding protein NikE |  |  | 2.1 |  | 10.4 | 10.3 | 11.8 | 12.2 | 6.1 | 8.2 | 7.5 | 11.5 |
| SAUSA300_2408 | *-* | Nickel transport ATP-binding protein NikD |  |  |  |  | 9.7 | 9.2 | 11.2 | 12.5 | 6.0 | 8.0 | 7.3 | 11.8 |
| SAUSA300_2409 | *-* | Nickel transport system permease protein NikC |  |  |  |  | 8.9 | 9.3 | 10.6 | 12.8 | 6.2 | 7.6 | 7.5 | 12.1 |
| SAUSA300_2410 | *-* | Nickel transport system permease protein NikB |  |  | 2.8 |  | 8.6 | 10.6 | 13.1 | 16.6 | 5.4 | 6.7 | 6.8 | 9.8 |
| SAUSA300_2411 | *opp-1A* | Nickel-binding protein |  |  | 2.7 |  | 11.2 | 12.8 | 15.5 | 17.6 | 8.2 | 9.4 | 9.1 | 13.6 |
| SAUSA300_2412 | *-* | Hypothetical protein |  |  |  | -2.2 | 6.1 | 3.6 | 3.9 | 7.1 | 3.6 | 5.2 | 5.4 | 10.1 |
| SAUSA300_2413 | *-* | Methyltransferase (EC 2.1.1.-) |  | -2.2 |  | -3.2 | 5.7 | 2.9 | 3.5 | 7.1 | 2.9 | 3.9 | 4.5 | 7.7 |
| SAUSA300_2414 | *-* | Diaminopimelate epimerase (EC 5.1.1.7) |  | -2.9 | -2.5 | -3.5 | 5.1 | 2.7 | 3.2 | 6.4 | 2.7 | 4.0 | 4.3 | 7.7 |
| SAUSA300_2415 | *-* | Hypothetical protein | -2.3 |  |  |  | -4.5 | -3.2 | -2.6 | -2.6 | -2.5 | -2.7 |  | -2.2 |
| SAUSA300_2416 | *-* | Hypothetical protein | -5.1 | -2.0 |  | -2.4 | -14.4 | -7.1 | -5.5 | -5.1 | -5.8 | -5.8 | -3.4 | -2.8 |
| SAUSA300_2417 | *-* | Aminobenzoyl-glutamate transport protein | -3.5 |  | -4.5 |  |  |  |  |  |  |  |  |  |
| SAUSA300_2418 | *-* | Transposase | -3.4 |  |  |  | -18.3 | -8.8 | -10.1 | -10.2 | -3.4 | -5.0 | -4.2 | -4.7 |
| SAUSA300_2421 | *-* | Hypothetical protein | -2.0 |  |  |  | -2.4 |  |  |  |  |  |  |  |
| SAUSA300_2422 | *-* | Short chain dehydrogenase | -3.2 |  |  |  | -4.0 | -2.3 |  |  | -2.1 |  |  |  |
| SAUSA300_2423 | *-* | Hypothetical protein | -2.8 |  | 2.4 | 2.8 | -2.2 |  | 2.2 | 2.2 | -2.0 | -2.2 |  | 2.9 |
| SAUSA300_2429 | *-* | Hypothetical protein |  |  |  | 2.2 |  |  |  |  |  |  |  |  |
| SAUSA300_2431 | *-* | DNA/RNA helicase (DEAD/DEAH box family) | 2.1 | 2.6 | 2.4 | 2.6 | 2.4 | 2.5 | 2.1 | 2.0 | 2.2 |  |  |  |
| SAUSA300_2432 | *-* | 7,8-dihydro-8-oxoguanine-triphosphatase (EC 3.6.1.-) |  | 2.1 | 2.0 | 2.4 | 2.1 | 2.5 | 2.2 |  | 2.5 |  |  |  |
| SAUSA300_2433 | *-* | Phosphoglucomutase (EC 5.4.2.2) / Phosphomannomutase (EC 5.4.2.8) |  | 2.6 | 2.7 | 2.9 |  | 2.5 | 2.6 | 2.3 | 2.3 | 2.3 |  | 2.0 |
| SAUSA300_2435 | *-* | Beta-N-acetylhexosaminidase (EC 3.2.1.52) |  |  |  |  |  |  |  | 2.1 | -2.4 |  |  |  |
| SAUSA300_2436 | *-* | Beta-N-acetylhexosaminidase (EC 3.2.1.52) | -2.1 |  |  |  |  |  |  |  |  |  |  |  |
| SAUSA300_2439 | *galU* | UTP--glucose-1-phosphate uridylyltransferase (EC 2.7.7.9) | 2.9 | 3.8 | 4.0 | 4.1 | 2.9 | 3.6 | 3.3 | 2.8 | 2.1 |  |  |  |
| SAUSA300_2440 | *fnbB* | Fibronectin-binding protein | 6.5 |  |  |  | 10.5 | -2.4 |  |  | -2.5 |  | 4.5 | 4.7 |
| SAUSA300_2441 | *fnbA* | Fibronectin-binding protein | 5.3 | 2.4 |  | 2.6 | 5.8 |  | 2.8 | 5.3 |  | 4.7 | 12.3 | 10.2 |
| SAUSA300_2442 | *gntP* | Gluconate permease | -4.6 | -2.5 |  | -5.0 | -3.8 | -4.9 | -3.7 | -2.7 | -2.5 |  |  |  |
| SAUSA300_2443 | *gntK* | Gluconokinase (EC 2.7.1.12) | -3.2 |  | 2.0 | -3.4 |  | -2.8 |  | -2.1 |  |  | 2.1 |  |
| SAUSA300_2444 | *gntR* | Gluconate operon transcriptional repressor | -3.4 | 2.4 | 2.9 | -3.4 | -2.6 | -3.0 |  | -2.5 |  |  |  |  |
| SAUSA300_2447 | *-* | Hypothetical protein | -6.7 | -11.3 | -9.4 | -5.7 | -22.3 | -20.9 | -12.2 | -4.5 | -18.3 | -10.5 | -3.8 | -4.0 |
| SAUSA300_2448 | *-* | Integral membrane protein | -6.5 | -13.7 | -9.0 | -8.8 | -7.5 | -20.3 | -25.2 | -29.1 | -9.1 | -12.6 | -13.9 | -12.1 |
| SAUSA300_2449 | *-* | Transporter, MFS superfamily |  |  |  | -3.6 |  | -2.6 | -2.5 | -3.1 |  |  |  |  |
| SAUSA300_2450 | *-* | DedA family protein |  |  |  |  |  |  |  |  | -3.2 | -2.2 |  |  |
| SAUSA300_2453 | *-* | Lantibiotic transport ATP-binding protein |  | 3.6 | 3.2 | 6.4 | 8.2 | 13.1 | 14.4 | 14.9 | 7.2 | 11.1 | 8.5 | 7.3 |
| SAUSA300_2454 | *-* | ABC transporter ATP-binding protein |  | 2.3 | 2.6 | 4.4 | 5.0 | 6.7 | 7.8 | 8.5 | 5.1 | 6.8 | 6.2 | 5.7 |
| SAUSA300_2455 | *-* | Fructose-1,6-bisphosphatase (EC 3.1.3.11) | -3.8 |  |  | -2.3 | -2.5 | -3.8 | -3.1 | -3.6 |  | 3.2 | 2.2 |  |
| SAUSA300_2457 | *-* | Carboxylesterase (EC 3.1.1.1) |  |  |  |  |  |  |  |  | 10.5 | 17.7 | 11.9 | 8.9 |
| SAUSA300_2458 | *-* | Glyoxalase family protein |  |  |  |  |  |  |  |  | 10.4 | 18.2 | 9.0 | 6.0 |
| SAUSA300_2459 | *-* | Transcriptional regulator, MarR family | 3.2 |  |  |  | 2.5 |  |  |  | 5.6 | 7.6 | 4.8 | 2.6 |
| SAUSA300_2460 | *-* | Acetyltransferase (EC 2.3.1.-) | -2.1 |  |  |  | -2.9 |  |  |  | -3.3 | -4.0 | -2.2 |  |
| SAUSA300_2461 | *-* | Glyoxalase family protein |  |  |  |  | -2.1 |  |  |  |  | 2.8 |  |  |
| SAUSA300_2462 | *frp* | NAD(P)H-dependent quinone reductase (EC 1.-.-.-) |  |  |  |  |  |  |  |  | 2.0 | 3.2 | 2.1 |  |
| SAUSA300_2463 | *ddh* | D-2-hydroxyacid dehydrogenase (EC 1.1.1.-) | -2.2 | 3.1 | 3.8 | 3.6 | -2.4 |  |  |  |  |  |  |  |
| SAUSA300_2467 | *srtA* | Sortase |  |  | -2.1 |  |  |  |  |  | -2.3 | -2.0 |  |  |
| SAUSA300_2468 | *-* | Phosphinothricin N-acetyltransferase (EC 2.3.1.-) | 5.6 | 2.3 | 2.8 |  |  |  |  |  |  |  |  |  |
| SAUSA300_2469 | *sdaAA* | L-serine dehydratase alpha subunit (EC 4.3.1.17) |  | -2.2 |  | -2.4 |  | -3.2 | -2.8 | -2.8 |  | -2.2 |  |  |
| SAUSA300_2470 | *sdaAB* | L-serine dehydratase beta subunit (EC 4.3.1.17) |  | -3.2 |  | -5.6 |  | -5.5 | -4.8 | -5.0 | -3.7 | -4.1 | -3.7 | -3.5 |
| SAUSA300_2471 | *-* | Transcriptional regulator pfoR |  | -4.5 |  | -7.6 |  | -6.5 | -5.3 | -5.5 | -4.4 | -4.9 | -4.4 | -3.9 |
| SAUSA300_2472 | *-* | Transporter, drug/metabolite exporter family | 2.1 |  |  |  |  |  |  | 2.6 |  |  |  |  |
| SAUSA300_2473 | *-* | Esterase (EC 3.1.1.-) |  |  |  | 2.5 |  |  |  |  |  |  |  |  |
| SAUSA300_2474 | *-* | Thioredoxin |  |  |  | 2.8 |  |  |  |  | 2.1 | 3.5 | 3.0 |  |
| SAUSA300_2475 | *-* | Thioesterase (EC 3.1.2.-) |  |  |  | 3.1 |  |  |  |  | 2.4 | 3.9 | 2.3 |  |
| SAUSA300_2476 | *ptsG* | PTS system, glucose-specific IIABC component (EC 2.7.1.69) |  |  | 2.6 |  | 2.0 |  |  |  |  |  |  |  |
| SAUSA300_2477 | *cidC* | Pyruvate dehydrogenase [cytochrome] (EC 1.2.2.2) | -4.6 | -2.4 |  | -2.2 | -19.7 | -8.1 | -5.7 | -4.3 | -4.2 | -4.2 |  |  |
| SAUSA300_2478 | *cidB* | Murein hydrolase export regulator | -5.9 | -2.9 | -2.1 | -2.4 | -21.7 | -10.1 | -7.0 | -6.3 | -6.6 | -7.4 | -2.8 |  |
| SAUSA300_2479 | *cidA* | Murein hydrolase exporter |  |  |  | 3.6 | -2.6 | -2.6 |  |  |  | -2.1 |  |  |
| SAUSA300_2480 | *-* | Hypothetical protein |  |  |  |  |  | -2.5 | -2.5 | -2.2 | -2.1 |  | -2.1 | -2.1 |
| SAUSA300_2482 | *-* | SceB precursor | 10.8 |  |  | 2.9 | 5.9 | 8.4 | 8.4 | 10.2 | 8.7 | 10.1 | 8.5 | 10.7 |
| SAUSA300_2484 | *-* | Hydroxymethylglutaryl-CoA synthase (EC 2.3.3.10) |  |  |  |  |  |  |  |  | 2.0 |  |  |  |
| SAUSA300_2486 | *-* | ATP-dependent endopeptidase clp ATP-binding subunit ClpL | -2.1 | 2.1 | 6.2 | 7.5 | -3.5 |  | 4.2 | 5.7 |  |  | 8.4 | 11.2 |
| SAUSA300_2488 | *feoA* | Ferrous iron transport protein A |  |  |  | -2.7 |  | -2.3 | -2.1 | -2.2 |  | -2.7 | -2.5 |  |
| SAUSA300_2490 | *-* | Transcriptional regulator, TetR family | 2.3 |  |  |  | 2.8 | 2.4 |  | 2.0 | 2.8 | 2.1 |  |  |
| SAUSA300_2491 | *-* | Delta-1-pyrroline-5-carboxylate dehydrogenase (EC 1.5.1.12) | -4.8 |  | 2.2 |  | -2.9 | -2.6 | -2.1 | -2.7 |  | 2.1 | 2.2 |  |
| SAUSA300_2492 | *-* | O-acetyltransferase (cell wall biosynthesis) (EC 2.3.1.-) |  | 2.3 | 2.0 | 3.7 |  |  |  |  |  | 2.9 | 2.2 |  |
| SAUSA300_2493 | *-* | Hypothetical protein |  |  | 2.1 |  |  |  |  |  | 6.9 | 12.9 | 12.7 | 6.3 |
| SAUSA300_2494 | *-* | Copper-exporting ATPase (EC 3.6.3.4) |  |  |  |  |  |  |  |  | 3.9 | 4.4 | 3.3 | 2.6 |
| SAUSA300_2495 | *-* | Hypothetical protein |  |  | 2.5 | 2.0 |  |  |  |  | 2.4 | 3.0 | 3.2 | 2.4 |
| SAUSA300_2496 | *-* | D-2-hydroxyacid dehydrogenase (EC 1.1.1.-) | -2.3 |  |  |  |  | 3.8 | 4.7 | 7.2 |  | 2.7 | 3.4 | 2.7 |
| SAUSA300_2497 | *-* | Aspartate aminotransferase (EC 2.6.1.1) | -2.7 |  |  |  |  | 3.9 | 4.5 | 6.6 | 2.4 | 3.1 | 3.6 | 2.9 |
| SAUSA300_2498 | *crtN* | Dehydrosqualene desaturase (EC 1.3.99.-) | -4.6 | -2.9 |  | -3.4 | -10.9 | -8.3 | -6.6 | -5.7 | -7.5 | -8.0 | -4.8 | -4.0 |
| SAUSA300_2499 | *crtM* | dehydrosqualene synthase (EC 2.5.1.-) | -7.3 | -4.3 | -2.0 | -5.1 | -22.2 | -12.4 | -9.8 | -8.9 | -12.3 | -11.0 | -6.7 | -5.0 |
| SAUSA300_2500 | *-* | HpnB protein | -10.6 | -5.3 | -2.6 | -6.9 | -34.2 | -17.1 | -14.6 | -11.8 | -13.4 | -13.8 | -6.6 | -4.5 |
| SAUSA300_2501 | *-* | Phytoene desaturase (EC 1.14.99.-) | -11.3 | -3.5 | -2.3 | -5.7 | -32.7 | -15.0 | -12.8 | -10.4 | -9.4 | -9.0 | -4.6 | -3.9 |
| SAUSA300_2502 | *-* | Hypothetical protein | -11.0 | -3.1 | -2.1 | -5.7 | -29.7 | -17.5 | -13.0 | -13.8 | -11.2 | -10.4 | -4.9 | -4.2 |
| SAUSA300_2503 | *-* | Secretory antigen precursor SsaA | 2.9 |  | -2.2 | -2.3 |  |  |  |  |  |  |  | 2.5 |
| SAUSA300_2505 | *-* | Acetyltransferase, GNAT family | 12.2 | 6.3 | 6.7 | 4.7 | 10.6 | 12.0 | 11.4 | 7.8 | 7.8 | 9.4 | 6.5 | 6.6 |
| SAUSA300_2506 | *isaA* | Hypothetical protein | 6.9 | 3.7 | 3.7 | 4.5 | 5.6 | 7.3 | 6.5 | 5.3 | 5.1 | 5.9 | 4.8 | 5.1 |
| SAUSA300_2507 | *-* | Hypothetical membrane spanning protein | 2.4 |  |  |  | 2.0 | 6.6 | 17.1 | 71.5 | 3.6 | 9.0 | 18.7 | 15.6 |
| SAUSA300_2509 | *-* | Transcriptional regulator, TetR family | 3.5 | 2.9 |  | 3.1 | 2.8 | 3.2 | 2.9 | 3.7 |  | 2.7 | 2.3 |  |
| SAUSA300_2510 | *-* | Hypothetical protein |  |  |  |  |  |  |  |  |  |  | 2.0 | 2.0 |
| SAUSA300_2511 | *-* | Hypothetical cytosolic protein |  | 2.3 |  |  |  |  |  | 3.1 | 2.5 | 4.1 | 4.6 | 3.5 |
| SAUSA300_2512 | *-* | Glyoxalase family protein | 2.3 | 2.3 |  |  |  | 2.0 |  |  |  |  |  |  |
| SAUSA300_2513 | *-* | putative nucleoside-diphosphate-sugar epimerases | 2.8 | 2.5 |  |  |  | 2.8 |  |  |  |  |  |  |
| SAUSA300_2514 | *-* | Hypothetical protein | 3.1 | 2.1 |  |  | 2.0 | 2.9 | 2.0 |  |  |  |  |  |
| SAUSA300_2515 | *-* | Transcriptional regulator, TetR family |  | 2.1 |  |  |  |  |  |  |  |  |  |  |
| SAUSA300_2516 | *-* | Short chain dehydrogenase |  | 2.7 |  |  |  |  |  |  | 2.3 | 2.5 | 2.1 |  |
| SAUSA300_2517 | *-* | 5-carboxyvanillic acid decarboxylase (EC 4.1.1.-) |  |  |  |  | -2.0 |  |  |  |  |  |  |  |
| SAUSA300_2518 | *-* | Esterase/Lipase (EC 3.1.-.-) |  |  | 2.0 |  | -3.3 |  |  |  |  |  |  |  |
| SAUSA300_2522 | *-* | Hypothetical cytosolic protein | 2.3 |  | 2.3 |  | 2.4 |  |  | -2.3 |  |  | -2.0 | -2.3 |
| SAUSA300_2523 | *-* | Hypothetical protein | 2.4 |  | 2.1 |  | 2.7 |  |  | -2.7 |  |  | -2.3 | -2.5 |
| SAUSA300_2524 | *-* | Hypothetical protein | 2.3 |  | 2.2 |  | 2.8 |  |  | -2.1 |  |  | -2.2 | -2.9 |
| SAUSA300_2525 | *-* | Fructosamine kinase family protein | -2.2 |  | 3.2 |  | -3.7 | -2.2 |  |  |  |  |  | 2.5 |
| SAUSA300_2526 | *pyrD* | Dihydroorotate dehydrogenase (EC 1.3.3.1) |  |  | 2.9 | 2.7 | 2.8 |  |  |  |  |  |  |  |
| SAUSA300_2527 | *-* | Hypothetical protein |  |  | 3.5 | 2.4 | 2.4 |  |  |  |  |  |  |  |
| SAUSA300_2529 | *-* | PhnB protein |  |  |  |  |  |  |  | -3.0 |  |  |  |  |
| SAUSA300_2532 | *panD* | Aspartate 1-decarboxylase (EC 4.1.1.11) |  | 2.4 |  | 2.5 |  |  |  |  |  |  |  |  |
| SAUSA300_2533 | *panC* | Pantoate--beta-alanine ligase (EC 6.3.2.1) | 2.4 | 3.1 | 2.1 | 3.2 | 2.5 |  | 2.3 | 3.1 |  |  |  |  |
| SAUSA300_2534 | *panB* | 3-methyl-2-oxobutanoate hydroxymethyltransferase (EC 2.1.2.11) | 3.2 | 3.2 |  | 3.7 | 3.1 | 2.6 | 2.8 | 3.7 |  | 2.1 |  |  |
| SAUSA300_2536 | *budA* | Alpha-acetolactate decarboxylase (EC 4.1.1.5) | 2.0 | 2.3 | 2.8 | 3.5 | 2.3 | 3.2 | 4.3 | 4.5 | 2.3 | 2.3 | 2.3 | 3.1 |
| SAUSA300_2537 | *-* | L-lactate dehydrogenase (EC 1.1.1.27) |  | 2.7 | 4.9 | 7.4 |  | 5.3 | 7.3 | 8.0 | 2.1 | 3.0 | 4.9 | 6.3 |
| SAUSA300_2538 | *-* | Amino acid permease | -2.0 |  | -3.7 | -2.0 |  | 3.4 | 5.3 | 12.2 |  | 2.7 | 3.8 | 3.3 |
| SAUSA300_2539 | *-* | 4-aminobutyrate aminotransferase (EC 2.6.1.19) |  |  |  |  |  | 12.0 | 19.2 | 55.3 | 4.4 | 9.6 | 16.4 | 11.8 |
| SAUSA300_2540 | *-* | Fructose-bisphosphate aldolase (EC 4.1.2.13) | -3.0 |  |  |  |  | 2.5 | 2.4 |  |  |  | 2.2 | 2.3 |
| SAUSA300_2543 | *-* | Signal transduction protein TRAP | -2.4 |  |  | -2.8 | -3.1 | -5.2 | -4.9 | -4.7 | -3.8 | -3.8 |  |  |
| SAUSA300_2546 | *betB* | Betaine aldehyde dehydrogenase (EC 1.2.1.8) |  | 2.0 |  |  |  |  |  |  |  |  |  |  |
| SAUSA300_2547 | *-* | Transcriptional regulator, ArsR family | 2.6 |  |  |  | 2.1 |  |  |  |  |  |  |  |
| SAUSA300_2548 | *-* | Zinc metallohydrolase, glyoxalase ii family | -2.8 | -2.3 | -3.3 | -2.3 | -4.4 | -4.5 | -4.6 | -4.1 | -3.3 | -3.7 | -2.5 |  |
| SAUSA300_2549 | *bccT* | Choline transport protein |  |  |  |  |  |  |  |  |  |  |  |  |
| SAUSA300_2550 | *nrdG* | Anaerobic ribonucleoside-triphosphate reductase activating protein (EC 1.97.1.4) | -2.4 |  | 3.4 | 19.7 |  | 3.2 | 8.3 | 17.6 | -2.0 |  | 3.7 | 8.9 |
| SAUSA300_2551 | *nrdD* | Anaerobic ribonucleoside-triphosphate reductase (EC 1.17.4.2) |  | 2.2 | 4.0 | 30.4 |  | 5.6 | 13.1 | 30.7 |  |  | 4.8 | 10.3 |
| SAUSA300_2552 | *-* | Hypothetical protein |  |  |  | 2.1 |  |  |  | 3.0 |  |  |  |  |
| SAUSA300_2553 | *-* | Precorrin-2 dehydrogenase (EC 1.3.1.76) / Sirohydrochlorin ferrochelatase (EC 4.99.1.4) |  |  |  |  |  |  |  | 2.7 |  |  |  |  |
| SAUSA300_2554 | *-* | Sulfite reductase [NADPH] flavoprotein alpha-component (EC 1.8.1.2) | 2.2 | 2.4 |  | 2.3 |  | 2.4 | 2.6 | 5.0 |  | 2.4 |  |  |
| SAUSA300_2555 | *-* | Glutathione peroxidase (EC 1.11.1.9) |  |  |  |  |  |  |  |  |  | 2.2 |  |  |
| SAUSA300_2561 | *phoB* | Alkaline phosphatase (EC 3.1.3.1) |  |  |  |  |  |  |  |  |  |  |  | 2.0 |
| SAUSA300_2564 | *estA* | Acetyl esterase (EC 3.1.1.-) |  | 2.5 | 2.0 | 2.5 |  | 2.3 | 2.5 | 2.8 |  |  |  |  |
| SAUSA300_2565 | *clfB* | Clumping factor |  | -2.6 | -7.2 | -5.2 |  | -3.7 | -3.8 | -3.1 | -3.5 | -3.7 | -3.9 | -3.5 |
| SAUSA300_2566 | *arcR* | Transcription regulator, Crp family |  |  |  |  |  |  |  |  | -2.0 |  |  |  |
| SAUSA300_2567 | *arcC* | Carbamate kinase (EC 2.7.2.2) |  |  |  |  |  |  |  | -2.0 |  |  |  |  |
| SAUSA300_2569 | *arcB* | Ornithine carbamoyltransferase (EC 2.1.3.3) |  |  | 2.4 |  | 2.4 |  |  | 2.0 |  |  | 2.6 |  |
| SAUSA300_2570 | *arcA* | Arginine deiminase (EC 3.5.3.6) |  |  |  |  | 2.3 |  |  |  | 2.5 | 2.7 | 3.0 | 3.7 |
| SAUSA300_2572 | *aur* | Zinc metalloproteinase aureolysin (EC 3.4.24.29) | -2.3 | -4.0 | -2.9 |  |  |  |  |  |  |  |  |  |
| SAUSA300_2573 | *isaB* | Hypothetical protein | -2.5 |  |  | 2.1 |  |  |  |  |  |  | 2.7 | 3.8 |
| SAUSA300_2574 | *-* | Hypothetical exported protein |  |  |  | 2.1 |  |  |  |  |  |  |  |  |
| SAUSA300_2575 | *-* | Transcription antiterminator, BglG family |  | 2.2 | 3.0 | 2.4 |  |  |  | 2.4 |  |  |  |  |
| SAUSA300_2576 | *-* | PTS system, mannose-specific IIAB component (EC 2.7.1.69) / PTS system, mannose-specific IIC component (EC 2.7.1.69) | -3.3 | -2.7 | -2.2 | -2.1 | -2.3 | -3.2 |  |  |  |  |  |  |
| SAUSA300_2577 | *manA* | Mannose-6-phosphate isomerase (EC 5.3.1.8) | -3.0 |  | -2.1 |  | -2.3 | -2.9 |  |  |  |  |  |  |
| SAUSA300_2580 | *-* | N-carbamoylsarcosine amidase (EC 3.5.1.59) |  |  |  |  | -2.2 |  |  |  |  |  |  |  |
| SAUSA300_2582 | *-* | surface protein Pls |  |  |  |  | -2.9 | -2.7 | -2.6 | -2.2 | -3.4 | -3.1 | -2.6 | -3.1 |
| SAUSA300_2583 | *-* | Poly(Glycerol-phosphate) alpha-glucosyltransferase (EC 2.4.1.52) |  |  |  |  | -2.3 | -2.1 |  |  | -2.2 | -2.2 |  |  |
| SAUSA300_2584 | *-* | Protein translocase subunit SecA | -2.6 | -2.1 |  |  | -3.4 | -3.1 | -2.9 | -2.4 | -3.2 | -3.4 | -2.1 |  |
| SAUSA300_2585 | *-* | Hypothetical cytosolic protein | -2.8 | -3.0 | -2.9 |  | -2.7 | -3.4 | -4.4 | -4.3 | -2.6 | -3.3 | -3.3 | -2.5 |
| SAUSA300_2586 | *-* | Hypothetical cytosolic protein | -2.7 | -3.1 | -2.7 |  | -2.7 | -3.9 | -4.0 | -3.4 | -2.4 | -3.5 | -3.2 | -2.1 |
| SAUSA300_2587 | *-* | Hypothetical protein | -2.8 | -3.1 | -2.8 |  | -3.0 | -4.5 | -4.9 | -3.9 | -2.8 | -4.5 | -4.2 | -4.4 |
| SAUSA300_2588 | *secY* | Protein translocase subunit SecY | -2.3 | -3.3 | -2.4 |  | -2.2 | -3.6 | -3.7 | -3.3 | -2.8 | -3.3 | -3.5 | -3.1 |
| SAUSA300_2589 | *-* | Cell surface protein | -2.6 | -3.4 |  |  | -2.5 | -3.9 | -4.2 | -3.5 | -2.3 | -4.5 | -4.7 | -4.2 |
| SAUSA300_2590 | *-* | FAD-dependent oxidoreductase (EC 1.-.-.-) |  |  |  |  | -2.4 | -2.2 |  |  | -2.2 |  |  |  |
| SAUSA300_2592 | *-* | Hypothetical protein | -4.3 | -3.3 | -2.2 | -3.0 | -13.6 | -10.5 | -9.0 | -9.3 | -8.8 | -8.9 | -4.2 | -3.8 |
| SAUSA300_2593 | *-* | FAD-dependent oxidoreductase (EC 1.-.-.-) | -2.3 |  |  |  | -3.3 | -2.6 | -2.6 | -2.2 | -3.4 | -3.2 | -2.6 | -2.3 |
| SAUSA300_2596 | *cap1C* | Phosphotyrosine-protein phosphatase (capsular polysaccharide biosynthesis) (EC 3.1.3.48) |  |  |  |  |  |  |  |  |  | -2.0 |  |  |
| SAUSA300_2598 | *cap1A* | Chain length regulator (capsular polysaccharide biosynthesis) |  | -2.4 | -2.8 | -2.3 |  |  |  |  |  | -2.1 | -2.5 | -2.2 |
| SAUSA300_2599 | *tetR* | Transcriptional regulator IcaR |  |  |  |  |  |  |  | -3.5 |  |  |  | -2.3 |
| SAUSA300_2601 | *icaB* | Polysaccharide deacetylase |  |  |  |  |  |  |  |  |  |  |  | 2.3 |
| SAUSA300_2603 | *lip* | Lipase (EC 3.1.1.3) | -12.9 | -3.2 | -2.7 | -8.1 | -7.6 | -21.5 | -17.0 | -17.2 |  |  |  | -2.4 |
| SAUSA300_2605 | *hisIE* | Phosphoribosyl-AMP cyclohydrolase (EC 3.5.4.19) / Phosphoribosyl-ATP diphosphatase (EC 3.6.1.31) | -2.1 |  |  | -2.6 |  |  |  | 2.2 |  |  |  |  |
| SAUSA300_2606 | *hisF* | Imidazole glycerol phosphate synthase, cyclase subunit (EC 4.1.3.-) |  |  |  | -2.1 |  |  |  | 2.3 |  |  |  | 2.2 |
| SAUSA300_2607 | *hisA* | 1-(5-phosphoribosyl)-5-[(5-phosphoribosylamino)methylideneamino] imidazole-4-carboxamide isomerase (EC 5.3.1.16) |  |  |  | -2.0 |  |  | 2.1 | 3.2 |  |  | 2.1 | 2.1 |
| SAUSA300_2608 | *hisH* | Imidazole glycerol phosphate synthase, glutamine amidotransferase subunit (EC 2.4.2.-) |  |  |  |  |  |  | 2.1 | 2.9 |  |  | 2.6 | 2.5 |
| SAUSA300_2609 | *hisB* | Imidazoleglycerol-phosphate dehydratase (EC 4.2.1.19) |  |  |  |  |  |  | 2.5 | 4.1 |  | 2.0 | 2.9 | 2.7 |
| SAUSA300_2610 | *hisC* | Histidinol-phosphate aminotransferase (EC 2.6.1.9) |  |  |  |  |  |  | 2.9 | 4.3 |  | 2.3 | 3.4 | 2.9 |
| SAUSA300_2611 | *hisD* | Histidinol dehydrogenase (EC 1.1.1.23) |  |  |  |  |  |  | 3.5 | 5.4 |  | 2.4 | 3.3 | 3.1 |
| SAUSA300_2612 | *hisG* | ATP phosphoribosyltransferase (EC 2.4.2.17) |  |  |  |  |  | 2.0 | 3.8 | 5.1 | 2.0 | 2.3 | 3.3 | 3.5 |
| SAUSA300_2613 | *hisZ* | Hypothetical cytosolic protein |  |  |  |  |  |  | 3.6 | 4.3 | 2.3 | 2.7 | 4.5 | 3.6 |
| SAUSA300_2614 | *-* | Hypothetical protein |  |  |  |  |  |  | 3.1 | 6.1 |  | 2.2 | 2.2 |  |
| SAUSA300_2615 | *-* | Ribosomal-protein-alanine acetyltransferase (EC 2.3.1.128) |  |  |  |  |  | 2.3 | 3.8 | 7.2 |  |  |  |  |
| SAUSA300_2616 | *-* | Cobalt transport protein cbiQ | -2.1 |  | -2.0 |  |  | 2.1 | 3.6 | 7.2 |  | 3.1 | 3.3 | 2.1 |
| SAUSA300_2617 | *-* | Cobalt transport ATP-binding protein cbiO | -2.1 |  |  |  |  | 3.3 | 5.1 | 11.7 |  | 3.3 | 3.4 | 2.1 |
| SAUSA300_2618 | *-* | Hypothetical protein | -2.1 |  | -2.1 |  |  | 3.7 | 6.1 | 13.1 | 2.3 | 4.8 | 5.0 | 3.9 |
| SAUSA300_2619 | *-* | Hypothetical cytosolic protein |  |  |  |  |  | 5.4 | 8.2 | 18.4 | 3.7 | 8.1 | 6.8 | 5.5 |
| SAUSA300_2620 | *-* | Hypothetical exported protein |  |  |  | -2.6 | -3.8 | -3.7 | -3.2 | -3.4 | 2.6 | 4.7 | 2.7 |  |
| SAUSA300_2621 | *-* | Antibiotic-induced protein, Drp35 |  | 2.1 | 3.9 |  |  |  |  |  | 3.2 | 5.1 | 4.2 | 3.2 |
| SAUSA300_2622 | *-* | Rhodanese-related sulfurtransferases | 2.3 |  | -2.0 | -2.3 |  |  |  |  |  |  |  |  |
| SAUSA300_2623 | *pcp* | Pyrrolidone-carboxylate peptidase (EC 3.4.19.3) |  |  |  | 2.2 |  |  |  | 2.5 |  |  |  |  |
| SAUSA300_2624 | *-* | Hypothetical protein | 2.6 | 3.9 | 3.2 | 3.1 | 2.9 | 3.3 | 3.5 | 4.5 | 2.8 | 4.0 | 4.0 | 3.5 |
| SAUSA300_2625 | *-* | Transcriptional regulator, PadR family | 3.8 | 4.5 | 3.0 | 4.5 | 4.6 | 6.0 | 5.5 | 6.8 | 3.9 | 5.4 | 4.4 | 3.6 |
| SAUSA300_2626 | *-* | Hypothetical protein | -7.9 | -5.4 | -3.9 | -7.6 | -32.8 | -21.1 | -17.1 | -23.6 | -10.4 | -11.9 | -6.1 | -5.3 |
| SAUSA300_2627 | *-* | 2-oxoglutarate/malate translocator |  |  | 2.6 | 2.3 |  |  | 2.0 |  | 2.2 |  |  |  |
| SAUSA300_2629 | *-* | Prolyl-tRNA synthetase | -3.9 |  |  |  | -6.8 | -4.9 | -4.3 | -3.7 | -3.7 | -3.4 | -2.2 |  |
| SAUSA300_2630 | *nixA* | High-affinity nickel transport protein | -2.4 |  |  |  | -6.0 | -5.2 | -4.1 | -3.4 | -5.9 | -3.5 | -2.4 | -2.7 |
| SAUSA300_2631 | *-* | Hypothetical protein |  |  |  |  |  |  |  |  |  | 2.1 |  |  |
| SAUSA300_2632 | *-* | Hypothetical membrane spanning protein | -3.9 | -2.5 |  | -2.4 | -8.6 | -7.4 | -5.2 | -5.0 | -2.9 | -3.1 |  |  |
| SAUSA300_2635 | *-* | Hypothetical protein |  |  |  |  |  | -2.0 |  |  | -2.0 |  |  |  |
| SAUSA300_2639 | *-* | Cold shock protein |  | -3.3 | -4.9 | -2.8 | -3.4 | -3.3 | -2.7 | -2.4 | -3.6 | -3.4 | -2.2 |  |
| SAUSA300_2640 | *-* | Transcriptional regulator |  |  | 3.7 | 7.4 |  | 2.5 | 4.3 | 5.6 |  |  |  |  |
| SAUSA300_2641 | *-* | Permease |  | 2.2 | 4.1 | 8.7 |  | 3.7 | 6.9 | 8.8 |  |  |  | 2.9 |
| SAUSA300_2642 | *-* | Permease | 2.0 | 3.5 | 8.3 | 17.8 |  | 6.9 | 13.2 | 15.2 |  |  | 2.5 | 3.4 |
| SAUSA300_2643 | *-* | Chromosome partitioning protein ParB | 3.4 |  |  |  | 2.4 |  |  |  |  |  |  |  |
| SAUSA300_2644 | *gidB* | Methyltransferase GidB (EC 2.1.-.-) | 3.0 |  |  |  | 2.2 |  |  |  |  |  |  |  |
| SAUSA300_2645 | *gidA* | Glucose inhibited division protein A | 2.3 |  |  | -2.4 |  |  |  | -2.1 |  |  |  | -2.3 |
| SAUSA300_2646 | *trmE* | tRNA (5-carboxymethylaminomethyl-2-thiouridylate) synthase |  | -2.0 |  | -3.1 |  |  |  | -2.4 |  | -2.0 | -2.2 | -2.3 |
| SAUSA300_2647 | *rnpA* | Ribonuclease P protein component (EC 3.1.26.5) |  | -2.8 |  | -3.1 |  |  | -2.4 | -3.7 |  |  |  |  |
| SAUSA300_2648 | *rpmH* | LSU ribosomal protein L34P |  |  |  |  |  |  |  | -2.4 |  |  |  |  |
| SAUSA300_pUSA010002 | *-* | Hypothetical protein |  |  |  |  |  |  |  |  | 2.3 | 2.2 | 2.3 | 2.2 |
| SAUSA300_pUSA010002 | *-* | Hypothetical protein |  |  |  |  |  |  |  |  | 2.4 | 2.2 | 2.3 | 2.2 |
| SAUSA300_pUSA010003 | *-* | Hypothetical protein | 2.3 |  | 2.1 | 2.2 | 2.3 | 2.0 |  |  | 2.7 | 2.1 |  |  |
| SAUSA300_pUSA010003 | *-* | Hypothetical protein | 2.3 |  | 2.2 | 2.3 | 2.4 | 2.1 |  |  | 2.7 | 2.3 |  |  |
| SAUSA300_pUSA010004 | *-* | Hypothetical protein |  |  | 2.2 | 2.0 |  | 2.5 | 2.1 |  | 2.8 | 2.6 | 2.0 |  |
| SAUSA300_pUSA020001 | *tet* | Tetracycline resistance protein TetA(l)/TetK |  |  |  |  |  |  |  |  |  |  |  | 2.1 |
| SAUSA300_pUSA020003 | *-* | Replication initiation protein, RepA family |  |  |  |  |  |  |  |  | 2.2 |  |  | 2.7 |
| SAUSA300_pUSA030006 | *-* | Replication and maintenance protein |  |  |  |  |  |  |  |  |  |  |  | 2.7 |
| SAUSA300_pUSA030007 | *-* | rRNA adenine N-6-methyltransferase (EC 2.1.1.48) |  |  |  |  |  |  |  |  |  |  |  | 2.5 |
| SAUSA300_pUSA030009 | *-* | Hypothetical protein |  |  |  |  |  |  |  |  |  |  |  | 2.5 |
| SAUSA300_pUSA030010 | *-* | Hypothetical protein |  |  |  |  |  |  |  |  | 2.1 |  |  | 2.5 |
| SAUSA300_pUSA030012 | *-* | Hypothetical protein |  |  |  |  |  |  |  |  |  |  |  | 2.3 |
| SAUSA300_pUSA030014 | *-* | TrsE protein |  |  |  |  |  |  |  |  |  |  |  | 2.3 |
| SAUSA300_pUSA030015 | *-* | Hypothetical protein |  |  |  |  |  |  |  |  |  |  |  | 2.2 |
| SAUSA300_pUSA030016 | *-* | Secretory antigen precursor SsaA |  |  |  |  |  |  |  |  | 2.2 |  |  | 3.0 |
| SAUSA300_pUSA030018 | *-* | DNA topoisomerase III (EC 5.99.1.2) |  |  |  |  |  |  |  |  | 2.1 |  |  | 2.5 |
| SAUSA300_pUSA030020 | *-* | TraG/TraD family |  |  |  |  |  |  |  |  | 2.7 |  |  | 3.6 |
| SAUSA300_pUSA030021 | *-* | Hypothetical protein |  |  |  |  |  |  |  |  |  |  |  | 2.7 |
| SAUSA300_pUSA030023 | *-* | Hypothetical protein |  |  |  |  |  |  |  |  |  |  |  | 2.3 |
| SAUSA300_pUSA030027 | *-* | Hypothetical protein |  |  |  |  |  |  |  |  |  |  |  | 2.7 |
| SAUSA300_pUSA030029 | *-* | Hypothetical protein |  |  |  |  |  |  |  |  |  |  |  | 2.1 |
| SAUSA300_pUSA030030 | *-* | Hypothetical protein |  |  |  |  |  |  |  |  |  |  |  | 3.1 |
| SAUSA300_pUSA030034 | *-* | Hypothetical protein |  |  |  |  |  |  |  |  |  |  |  | 2.3 |
| SAUSA300_pUSA030035 | *-* | Hypothetical protein |  |  |  |  |  |  |  |  | 2.0 |  |  | 3.3 |
| SAUSA300_pUSA030036 | *-* | Hypothetical protein |  |  |  |  |  |  |  |  |  |  |  | 2.4 |

Microarray results are presented as the mean fold-change of six separate experiments. Data that met criteria for differentially-expressed genes are included in the Table (*P* value <0.05; >2-fold change). Fold-change for all conditions tested is expressed as transcript level relative to that at time = 0 in trypticase soy broth (TSB).
